# An HBV susceptibility variant of *KNG1* modulates the therapeutic effects of interferons α and λ1 in HBV infection by promoting MAVS lysosomal degradation

**DOI:** 10.1016/j.ebiom.2023.104694

**Published:** 2023-07-11

**Authors:** Bin Zhang, Haijun Han, Xinyi Zhao, Andria N. Li, Yan Wang, Wenji Yuan, Zhongli Yang, Ming D. Li

**Affiliations:** a State Key Laboratory for Diagnosis and Treatment of Infectious Diseases, National Clinical Research Center for Infectious Diseases, Collaborative Innovation Center for Diagnosis and Treatment of Infectious Diseases, The First Affiliated Hospital, Zhejiang University School of Medicine, Hangzhou, China; b Vanderbilt University School of Medicine, Nashville, TN, USA; c Research Center for Air Pollution and Health, Zhejiang University, Hangzhou, China

**Keywords:** *KNG1*, Functional SNP, HBV infection, Interferon, Treatment

## Abstract

**Background:**

Hepatitis B virus (HBV) infection is one of the main causes of hepatocellular carcinoma (HCC). The relationship between HBV infection and the host genome as well as their underlying mechanisms remain largely unknown.

**Methods:**

In this study, we performed a whole-genome exon sequencing analysis of 300 sib-pairs of Chinese HBV-infected families with the goal of identifying variants and genes involved in HBV infection. A site-direct mutant plasmid was used to investigate the function of SNP rs76438938 in *KNG1*. The functional and mechanical studies of *KNG1* were conducted with *in vitro* liver cell lines and a hydrodynamic injection model *in vivo*. The impact of *KNG1* on HBV infection therapy was determined in hepatocytes treated with IFN-α/λ1.

**Findings:**

Our whole-exon association study of 300 families with hepatitis B infection found that SNP rs76438938 in *KNG1* significantly increased the risk for HBV infection, and the rs76438938-T allele was found to promote HBV replication by increasing the stability of *KNG1* mRNA. By competitively binding HSP90A with MAVS, KNG1 can inhibit the expression of types I and III IFNs by promoting MAVS lysosomal degradation. Such suppression of IFN expression and promotion of HBV replication by *Kng1* were further demonstrated with an animal model *in vivo*. Lastly, we showed that the rs76438938-C allele can improve the therapeutic effect of IFN-α and -λ1 in HBV infection.

**Interpretation:**

This study identified a SNP, rs76438938, in a newly discovered host gene, *KNG1*, for its involvement in HBV infection and treatment effect through modulating the cellular antiviral process.

**Funding:**

This study was supported in part by the Independent Task of 10.13039/501100011441State Key Laboratory for Diagnosis and Treatment of Infectious Diseases of the First Affiliated Hospital of 10.13039/501100004835Zhejiang University, the China Precision Medicine Initiative (2016YFC0906300), and the Research Center for Air Pollution and Health of 10.13039/501100004835Zhejiang University.

**Research in context**

**Evidence before this study**

Host genetics significantly affects HBV infection, but the mechanism underlying this is largely unknown. *KNG1* is thought to play a role in the inflammatory response, but its function and mechanism in the immune response have never been addressed.

**Added value of this study**

The exome-wide association analysis identifies some HBV infection-associated variations, which include rs76438938 located in *KNG1*. SNP rs76438938-T promotes HBV replication and increases *KNG1* mRNA stability. By promoting lysosomal degradation of MAVS, KNG1 inhibits the expression of types I and III IFNs, which leads to increased HBV replication. Moreover, we found that SNP rs76438938-C can yield a better therapeutic effect of IFN-α and IFN-λ1 in HBV infection.

**Implications of all the available evidence**

We provide new insights into how *KNG1* plays a role in the cellular antiviral process. Our data demonstrate that rs76438938 is a functional variant affecting hepatitis B susceptibility and treatment. This study suggests a potential precise treatment strategy for patients carrying the rs76438938-T allele.

## Introduction

Hepatitis B virus (HBV) infection, one of the world's major public health problems, can lead to liver cirrhosis (LC) and hepatocellular carcinoma (HCC).^1^^,^^2^ There are about 300 million HBV infections worldwide, with a prevalence rate of 3.9%.^3^ The infection and treatment of hepatitis B are related to the genetic background of the host.^4^, ^5^, ^6^, ^7^ Several Asian genome-wide association (GWAS) studies have shown that SNPs in HLA genes are associated with chronic HBV infection, including HLA-DP (rs9277535, rs3077, rs9366816, rs9277542) and HLA-DQ (rs2856718, rs7453920, rs9276370, rs9276516, and rs7453920).^8^, ^9^, ^10^ Clinical studies identified a significant correlation between IL-18-607C/A and -137G/C (rs1946519 and rs187238) polymorphisms and the risk of HBV infection.^11^ Huang et al. found that the TT genotype of TLR3 rs3775290 was closely related to the reduction of Chronic hepatitis B (CHB) risk.^12^ A meta-analysis revealed the importance of TLR3 rs3775291 in HBV-related diseases.^13^ However, genetic factors that contribute to HBV infection have not yet been fully revealed, and many more such susceptibility genetic variations remain to be identified.^14^

HBV was previously considered a “stealth virus” because the expression of IFNs and other inflammatory factors was not observed in liver biopsies of chimpanzees infected with HBV.^15^ However, these results might reflect the fact that the IFN response is suppressed by HBV.^16^^,^^17^ A strong and specific innate immune response was detected in differentiated HepaRG cells transduced with recombinant baculovirus encoding the HBV genome, including IFN-β and other IFN-stimulating genes.^18^ A recent study showed that primary human hepatocytes (PHH) and fibroblasts (MPCC) respond to HBV infection and activate types I and III IFN responses.^19^ Sato et al. reported that the pre-genomic RNA (pgRNA) produced by HBV replication was recognized by the pattern recognition receptor (PRR) retinoic acid-inducible gene I (RIG-I) to trigger type III (rather than type I) IFN responses in hepatocytes and human liver chimeric mice.^20^

The type III IFN family is composed of three members: IFNL1 (IL-29), IFNL2 (IL-28A), and IFNL3 (IL-28B).^21^ Although type III IFN exerts biological functions by interacting with different cell surface receptors, it is regulated by a mechanism common to type I IFN.^22^ Pattern recognition receptors such as Toll-like receptors (TLR) and RIG-I-like receptors recognize viruses or other pathogens and bind to and transmit signals to mitochondrial antiviral-signaling (MAVS) sites (also known as Cardif, IPS-1, and VISA).^23^ Activated MAVS recruits and phosphorylates TBK1 and IRF3, leading to the production of types I and III IFNs.^24^

In this study, to identify new functional HBV susceptibility genes and genetic variants, we performed a genetic association analysis on an exome sequencing dataset of 300 sib-pairs HBV-infected family samples with similar genetic backgrounds and found that SNP rs76438938 and its host gene *KNG1* were significantly associated with HBV infection. We further conducted a series of *in vitro* and *in vivo* experiments, including gene silencing and hydrodynamic injection, to explore its function and potential mechanism. We revealed the important role of *KNG1* in HBV infection and demonstrated a new mechanism indicating that KNG1 induces MAVS lysosome degradation through competitive binding to HSP90A to inhibit the expression of types I and III IFNs. Additionally, the risk allele rs76438938-T increases the stability of *KNG1* mRNA, leading to increased susceptibility to HBV infection. Finally, we showed that SNP rs76438938 could modulate the therapeutic effect of IFN-α and -λ1 in HBV infection.

## Methods

### Participants

The three hundred sib-pairs regardless of gender used in the study were recruited at the First Affiliated Hospital of Zhejiang University School of Medicine and other neighboring hospitals or medical centers.^25^ Among these siblings, 300 with chronic hepatitis B virus infection (CHBVI) were identified as seropositive for either hepatitis B surface antigen (HBsAg) or HBV-DNA, whereas the corresponding sib-controls were negative for both (Supplementary Table S1). More detailed descriptions of the demographic and phenotypic characteristics of these subjects have been reported previously.^26^ All participants were of Chinese Han ethnicity. Informed written consent was obtained from each participant, and demographic and clinical data were collected by structured questionnaires. The project was approved by the Ethical Committee of the First Affiliated Hospital of Zhejiang University School of Medicine (Ref No. 2017-921).

### DNA isolation, library preparation, and whole-exome sequencing

Genomic DNA (gDNA) was extracted from whole blood using the Gentra Puregene Blood Kit (#158845, Qiagen) and stored at −80 °C until needed. One μg of DNA per sample was used as input material for sample preparation. Sequencing libraries were generated using NEBNext® Ultra™ DNA Library Prep Kit for Illumina (#E7103L, NEB, USA) according to the manufacturer’s recommendations, and index codes were used to attribute sequences to each sample. DNA fragments were end-polished, A-tailed, and ligated with the full-length adaptor for Illumina sequencing. PCR products were purified with the AMPure XP system, and libraries were analyzed for size distribution by Agilent 2100 Bioanalyzer and quantified with real-time PCR. The libraries were sequenced on an Illumina HiSeq2000 platform, and 150-bp paired-end reads were generated.

### Genome-wide association analysis

DNA-seq reads of each sample were aligned with the reference genome hg38 using a Burrows-Wheeler Aligner (BWA) under the default setting.^27^ After removal of PCR duplicates by Picard tools 1.119 (http://broadinstitute.github.io/picard/), single nucleotide variations (SNVs) were identified by the Genome Analysis Toolkit (GATK v.3.5). The statistics of each variant, including allele balance, depth of sequencing coverage, strand balance, and multiple quality metrics, were annotated using the GATK Variant Annotator.^28^^,^^29^ These statistics were used in an adaptive error model to estimate the probability that each SNV was a true one using the GATK Variant Quality Score Recalibration (VQSR).^28^^,^^29^

Then, SNVs were filtered at a VQSR truth sensitivity (TS) of 99.9% for further analysis. Functional variants were annotated using the ANNOVAR^30^; both individual and genetic variant quality controls were examined using PLINK (v.1.9). The QC steps were conducted under the following criteria: genotype call rate >95%, minor allele frequency (MAF) >1%, and *P* value of Hardy–Weinberg equilibrium (HWE) >1 × 10^−6^. After these QC and filtering steps, a total of 191,924 SNPs were retained for further analysis. Genome-wide association analysis was carried out under two approaches. The first approach was a logistic regression under an additive genetic model adjusted by age and sex as covariates. The second approach was the liberalization of the sibling transmission/disequilibrium test (sTDT) model adjusted for age, sex, and the first five principal components (PCs),^31^ which was realized with the family-based association for disease traits (DFAM) procedure implemented in PLINK.^32^ Only those overlapped SNPs with a *P* value <0.05 (Pearson Correlation Analysis) for both genetic models were retained for the investigation.

### Cell culturing

The HepG2 (RRID: CVCL_0027), HepG2.2.15 (RRID: CVCL_L855), and Huh7 (RRID: CVCL_0336) cells were maintained in 37% Dulbecco's Modified Eagle medium (#SH30022.01, HyClone, Logan, UT, USA) containing 10% fetal bovine serum (#10100147, GIBCO, Waltham, MA USA), penicillin G 100 U/ml, and streptomycin 100 μg/ml (#10378016, GIBCO) and incubated at 37 °C in a humidified incubator with 5% CO_2_. HepG2.2.15 cells were supplemented with G418 sulfate 400 μg/ml (#10131027, GIBCO). The L02 (RRID: CVCL_6926) was maintained in RPMI 1640 medium (#SH30027.01, HyClone, Logan, UT, USA), with the other conditions being the same. All cell lines were purchased from the China Center for Type Culture Collection (CCTCC) and tested negative for mycoplasma throughout the study. The short tandem repeat (STR) profiling was used to verify the identity of each cell line.

### Quantitative RT-PCR (qRT-PCR)

Total RNA was extracted from cell lines using TRIzol reagent (#15596018, Invitrogen, Carlsbad, CA, USA) according to the manufacturer’s instructions. Reverse-transcribed cDNA was synthesized using the iScript™ cDNA Synthesis Kit (#1708890, Bio-Rad, Shanghai) following the manufacturer’s protocol. Real-time PCR was performed with SYBR Premix Ex *Taq* (#RR420, TaKaRa). Glyceraldehyde 3-phosphate dehydrogenase (GAPDH) was used as the internal control for the amplification of mRNA. The comparative CT method was applied to calculate the relative mRNA expression. Primer sequences are displayed in Supplementary Table S2.

### Transfection

HepG2.2.15, L02, Huh7, and HepG2 cells (∼8 × 10^5^) were seeded at an appropriate equal number in 6-well plates. In each well, cells were transfected with the recommended concentration of siRNA or plasmids using Lipo3000 (#L3000015, Thermo Fisher) according to the manufacturer's instructions and harvested after 48–72 h for assays. All siRNAs were purchased from Genepharma (Genepharma, Shanghai, China). The siRNA sequences used in the study are displayed in Supplementary Table S3.

### Immunofluorescence assay

The PCAGGS-*HSP90A*-His and pcDNA3.1-*KNG1*-3 × Flag or pcDNA3.1-*MAVS*-Flag plasmids were transfected into HepG2.2.15 cells, which were grown on coverslips for 48 h. HepG2.2.15 cells were washed three times with PBS before fixation with 4% formaldehyde for 20 min. The fixed cells were permeabilized with 0.1% Triton X-100 for 10 min and then blocked with 5% bovine serum albumin for 1 h. Cells were incubated with the corresponding antibodies for 1 h at room temperature. After washing three times with TBST, cells were incubated for 1 h at room temperature with Alexa Fluor® 488 conjugated anti-rabbit IgG (#ab150077, Abcam) and Alexa Fluor® 647 anti-mouse IgG (#ab150115, Abcam). DAPI (#P0131, Beyotime) was used for nucleus staining. The intracellular localization was analyzed by a confocal microscope (Nikon, Tokyo, Japan).

### Generation of nucleotide base-editing cell lines

The BE4-Gam plasmid (Addgene plasmid #100806) was a gift from Dr. David Liu. A specific oligonucleotide targeting rs76438938 was designed according to the experimental methods.^33^ The BE4-Gam plasmid and oligonucleotides were cotransfected into L02 cells by Lipo3000. After 48 h of transfection, cells were placed in a 96-well plate to obtain a single clone. Gene-edited cell lines were obtained from these monoclonal cells and verified by Sanger sequencing.

### Western blotting

The transfected cells were lysed by RIPA buffer containing 1 mM phenylmethylsulfonyl fluoride (PMSF, #ST506, Beyotime) and quantified using the BCA protein assay (#P0010, Beyotime). Protein samples were diluted with 5× sample loading buffer and boiled at 100 °C for 10 min. Next, 30 μg of protein was separated by 8% sodium dodecyl sulfate-polyacrylamide gel electrophoresis and transferred to PVDF membranes (#IPVH00010, Millipore, St. Louis, USA). Membranes were blocked with 5% skim milk at room temperature for 1 h and then incubated with the appropriate primary antibody at 4 °C overnight. Membranes were washed with TBST followed by 1 h of incubation with secondary antibody at room temperature. Antibody-bound proteins were visualized by ECL Hyper film kit in a ChemiDoc MP imaging system (BioRad, Hercules, CA). For quantification, Image J (National Institutes of Health) was used to measure the densitometry of each band, which was then normalized to ACTB or GAPDH. Primary antibodies used for Western blotting were anti-KNG1 (11926-1-AP, RRID: AB_2133403), anti-RIG-I (20566-1-AP, RRID: AB_10700006), anti-MAVS (14341-1-AP, RRID: AB_10548408), anti-Flag (20543-1-AP, RRID: AB_11232216 and 66008-4-Ig, RRID: AB_2918475), anti-ACTB (20536-1-AP, RRID: AB_10700003), anti-HSP90A (A5006, RRID: AB_2863411), anti-His (66005-1-Ig, RRID: AB_11232599), and anti-GAPDH (AC033, RRID: AB_2769570). The secondary antibodies were goat anti-rabbit IgG (SA00001-2, RRID: AB_2722564) and goat anti-mouse IgG (AS003, RRID: AB_2769851). All these antibodies were purchased from Proteintech or Abclonal.

### Co-immunoprecipitation

After extracting cellular protein with the same procedures as used for Western blotting, 450 μl of lysate and 1 μg of specific antibody or IgG control of the corresponding species were incubated overnight at 4 °C with rotation. Then, 30 μl Protein A + G agarose gel beads (#P2019, Beyotime) were added to the mixture, and the incubation was continued for 3 more hours at 4 °C with rotation. After centrifugation at 2500 rpm for 5 min, the agarose was washed three times with 1 ml of PBS, and 30 μl of 1 × SDS sample buffer was added to the mixture and incubated at 100 °C for 10 min to elute the protein. Finally, the supernatant liquid was centrifuged to collect material for subsequent immunoblotting experiments.

### HepG2-NTCP cell line establishment and HBV infection

Lentivirus encoding sodium taurocholate co-transporting polypeptide (NTCP) was purchased from OBiO Technology (Shanghai, China). The virus was used to infect HepG2 cells in the presence of polybrene (5 mg/ml). After infection for 48 h, the cells were cultured in DEME medium containing 5 mg/ml puromycin (#1780151, GIBCO) for 7 days to generate a HepG2-NTCP cell line. The stable HepG2-NTCP cell line was confirmed by qRT-PCR and Western blotting. HBV infection was conducted as described previously.^34^ The HBV used in this study was derived from HepG2.2.15 cells and was quantified by qRT-PCR. HepG2-NTCP cells were preincubated for 24 h with 3% DMSO (#D2650, Sigma) and then infected with HBV in the presence of 4% PEG8000 (#A600433, Sangon Biotech) and 3% DMSO for 16 h.

### Animal studies

Hydrodynamic injection was used to transfect gene expression plasmids. Male C57BL/6 mice (6–8 weeks old, RRID: IMSR_JAX:000664) were purchased from Zhejiang Center of Laboratory Animals and randomized into two groups which were received hydrodynamically injected (HDI) intravenously over 5–8 s with pHBV (10 μg) along with vector (20 μg) or *Kng1*-Flag (20 μg) plasmids. The mixture of plasmids was suspended in a volume of saline equivalent to 10% of body weight. Each mouse was cared for under standard conditions according to institutional guidelines and was euthanized randomly under anesthesia after successful injection at a specific time point, and serum and liver samples were collected for further analysis. The experimental procedures used in the study were approved by the Institutional Animal Care and Use Committee of Zhejiang University (ZJCLA-IACUC-20030050).

### Detection of HBV-DNA, HBsAg, and HBeAg

After 48 h of transfection, the supernatant liquid was collected by centrifugation at 3000 rpm for 10 min at 4 °C. The HBV DNA content was measured by an HBV-DNA fluorescent quantitative PCR detection kit (Sansure Biotech, Hunan, China). Using the ARCHITECT kit (Abbott, Chicago, IL, USA), the concentrations of HBsAg and HBeAg were quantified by chemiluminescent particle immunoassay. All measurements were carried out at least three times according to the manufacturer's instructions.

### Statistical analysis

All experiments were repeated at least three times, and the data are shown as Mean ± Standard error of the mean (SEM). We used two-tailed unpaired *t*-tests to compare the two groups. *P* < 0.05 was considered statistically significant.

### Role of funders

The funders played no role in the study design, data collection, data analysis, data interpretation, and paper writing.

## Results

### Characterization of a functional variant rs76438938 in *KNG1* for affecting HBV infection

To identify susceptibility variants and genes for HBV infection, we performed a whole-exome sequencing analysis on 300 paired family samples. Because of a relatively small sample size, we did not find any SNP that met the significance threshold at the genome-wide level (*P* < 1 × 10^−8^, Pearson Correlation Analysis). Considering the purpose of this study was to find the variants that are more likely to be involved in HBV infection, the liberalization of the sibling transmission/disequilibrium test (sTDT) was also adopted for the study, which is commonly used for genetic association analysis of family study design (Supplementary Fig. S1a). Based on two association analysis methods, 639 SNPs across 534 genes were found to be associated with hepatitis B infection (*P* < 0.05, Pearson Correlation Analysis), which includes 3 nonsense variations and 180 missense variations (data not shown).

**Fig. 1:**
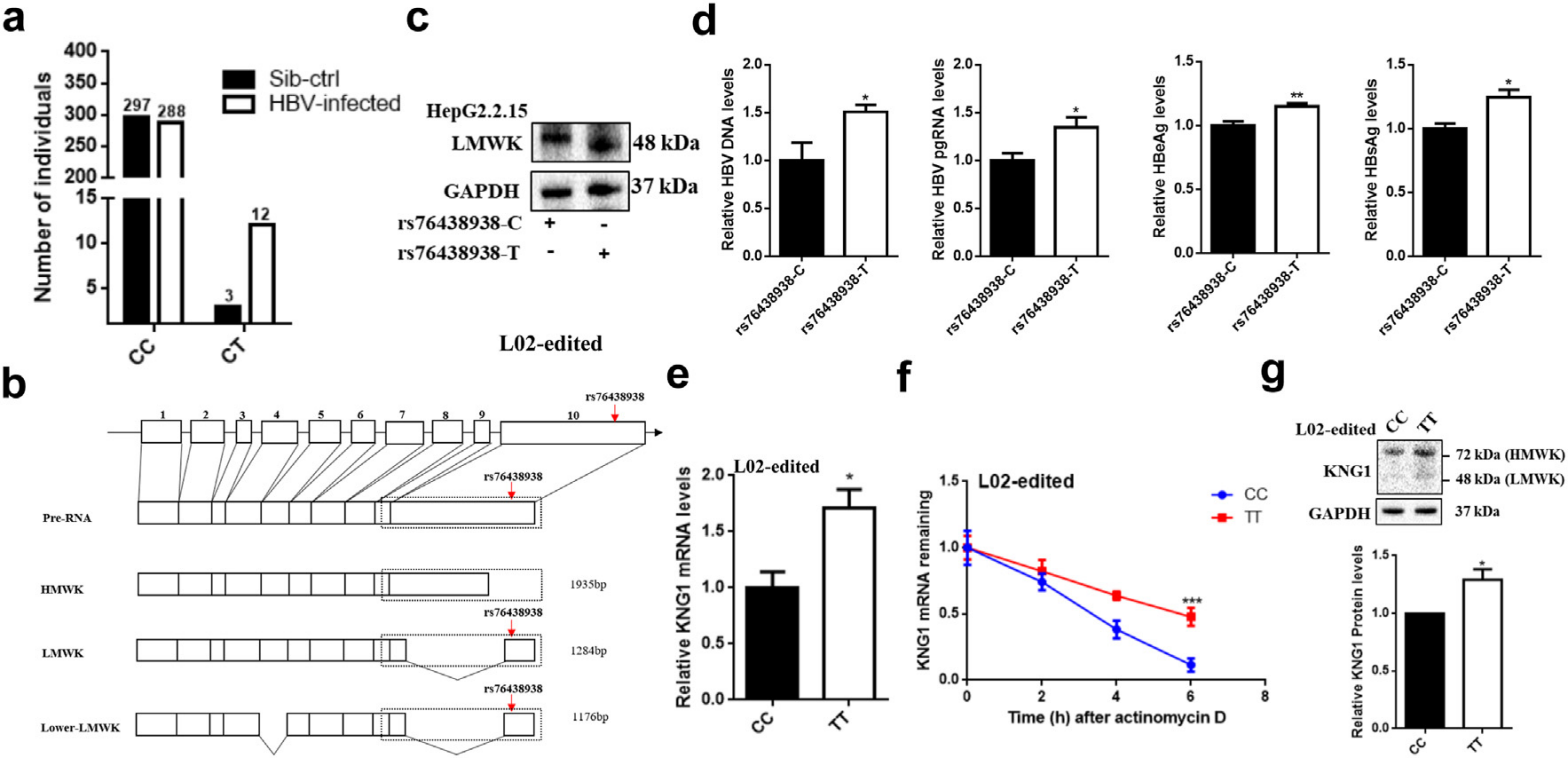
**Characterization of a functional variant rs76438938 in *KNG1* affecting HBV infection.** (a) Participants carrying the T allele and their sibs in family samples. (b) Schematic diagram of *KNG1* alternative splicing. The red arrow marks rs76438938. (c) Both wild-type and mutant *LMWK* were overexpressed in HepG2.2.15 cells. (d) Levels of HBV DNA, HBV pgRNA, HBeAg, and HBsAg in HepG2.2.15 cells transfected with wild-type or mutant *LMWK* plasmid. (e) qRT-PCR analysis of *KNG1* in L02 cells with CC and TT genotypes. (f) Effect of rs76438938 on the *KNG1* mRNA stability. Actinomycin D (5 μg/ml) was used to treat cells, which were collected at 0, 2, 4 and 6 h after treatment. The mRNA was detected by qRT-PCR normalized to *GAPDH*. (g) Western blotting analysis of *KNG1* in L02 cells with CC and TT genotypes. Error bars indicate SEM. *P*-value was determined using a two-tailed unpaired t-test. ∗*P* < 0.05; ∗∗*P* < 0.01; ∗∗∗*P* < 0.001.

To discover functional variants that are more likely to affect HBV infection, we focused on the three nonsense variants, which are more likely to have a large biological effect on their host gene (Supplementary Table S4). Examination of clinical data showed that 80% of individuals carrying the rs76438938-T allele had developed chronic hepatitis B and liver cirrhosis (OR = 4.6660, *P* = 0.0209, Pearson Correlation Analysis; Fig. 1a; Supplementary Table S5), indicating that SNP rs76438938-T in *KNG1* significantly enhances the susceptibility of carriers to HBV infection.

Gene annotation of the NCBI database revealed that *KNG1* is located on chromosome 3, and it is expressed mainly in the liver and kidneys. *KNG1* contains 10 exons and encodes two proteins through alternative splicing: high molecular weight kininogen (HMWK) and low molecular weight kininogen (LMWK) (Fig. 1b). The SNP rs76438938 is located in the 3′UTR of *HMWK* and the 10th exon of *LMWK*, which results in a truncation of 16 amino acids from the C-terminus of the LMWK.

To investigate the function of rs76438938 as a nonsense mutation in HBV infection, we constructed *LMWK* expression plasmids carrying either the C (i.e., full-length or wild-type LMWK) or T allele (i.e., truncated or mutant LMWK) and transfected them into HepG2.2.15 cells and then detected their effect on intracellular HBV replication level. As shown in Fig. 1c, the expression plasmids carrying either the rs76438938 C or T allele were successfully expressed in cells and the rs76438938-T allele resulted in a truncated LMWK protein. Compared with the HepG2.2.15 cells expressing full-length LMWK (i.e., C allele), qRT-PCR analysis showed significantly increased HBV DNA and HBV pgRNA in the cells expressing mutant LMWK, along with the higher levels of HBeAg and HBsAg detected by ELISA (Fig. 1d). These results suggest that SNP rs76438938-T promotes HBV replication in HepG2.2.15 cells.

Surprisingly, endogenous LMWK protein was not detected in HepG2.2.15 cells. Compared with the control group where HepG2 cells were transfected with the *LMWK* overexpression plasmid, the LMWK protein was not detected by Western blotting in normal HepG2 and HepG2.2.15 cells (Supplementary Fig. S1b). In addition, the LMWK protein was not also observed in mouse liver samples (Supplementary Fig. S1c). These data imply that HMWK is the main protein product of *KNG1*, while LMWK may only have a potential effect on HBV replication.

To further evaluate the effect of rs76438938 on *KNG1*, we generated a mutant cell line carrying the risk allele rs76438938-T by using the CRISPR-BE4-Gam system in L02 cells (Supplementary Fig. S1d). We found that rs76438938-T significantly increased the expression of *KNG1* (Fig. 1e). Actinomycin D treatment assays showed that the *KNG1* mRNA in mutant cells decayed slower relative to the wild-type cells, indicating that rs76438938-T is capable of increasing *KNG1* mRNA stability (Fig. 1f). At the protein level, a significant increase of the long form of KNG1 protein (i.e., HMWK) was detected in L02 cells carrying the rs76438938-T allele (Fig. 1g), and no short form of the protein (i.e., LMWK) was detected in this cell line. These results indicate that rs76438938 executes its biological function mainly by modulating *KNG1* expression during HBV infection. Since HMWK is the main protein of KNG1, we will use HMWK level to represent the protein level of KNG1 in the following description of this paper, unless stated otherwise.

### *KNG1* promotes HBV replication

**Fig. 2:**
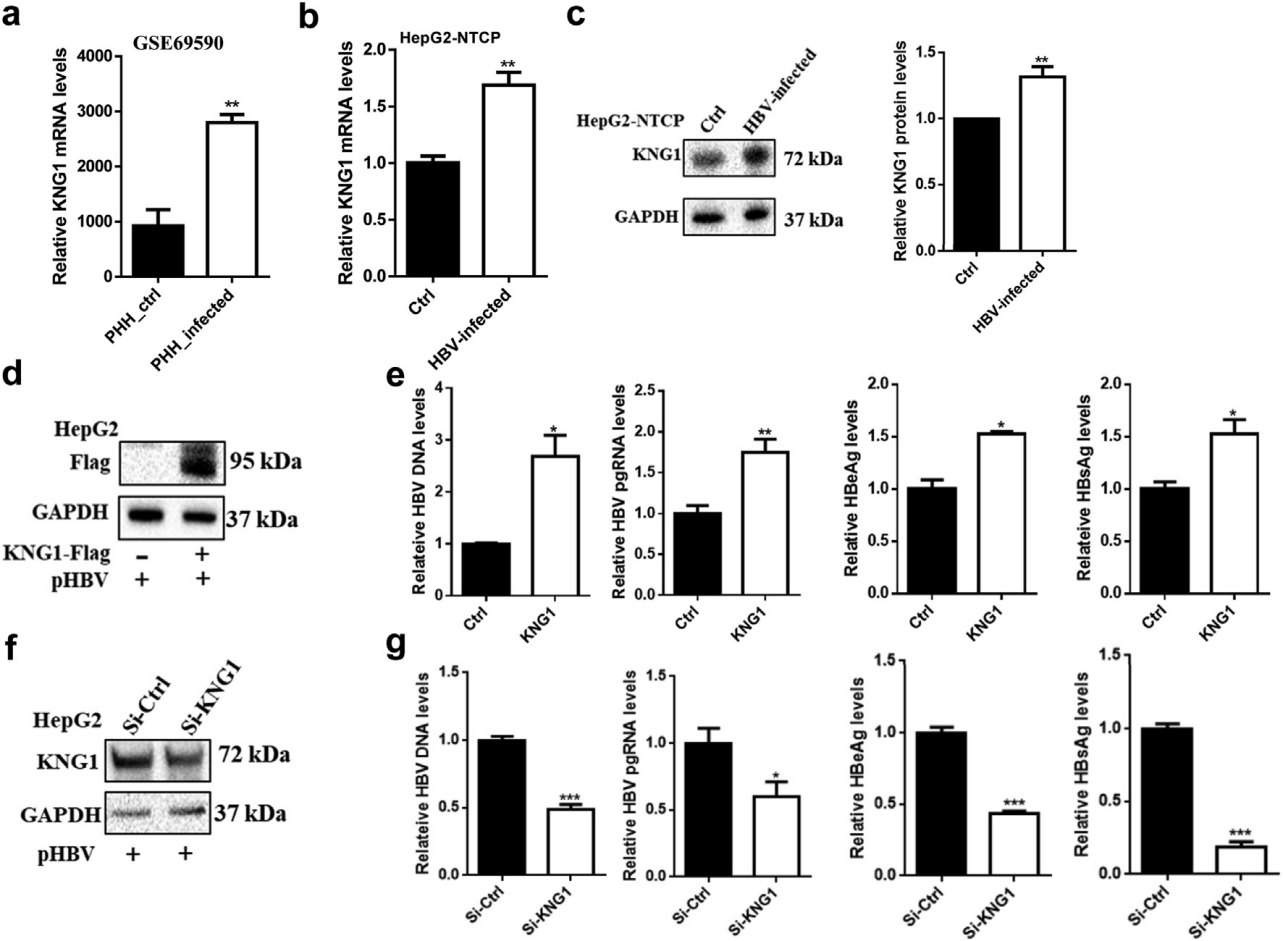
**Determination of *KNG1* in promoting HBV replication.** (a) The difference of *KNG1* expression level in PHHs before and after HBV infection for 40 h. The expression data was obtained from the GEO database (GSE69580). (b and c) Changes of *KNG1* mRNA and protein levels in HepG2-NTCP cells before and after HBV infection for 48 h. (d) Western blotting analysis of *KNG1* overexpression in HepG2 cells. Cells were transfected with pHBV and *KNG1*-Flag or vector plasmids for 48 h. (e) Levels of HBV DNA, HBV pgRNA, HBeAg, and HBsAg in HepG2 cells with *KNG1* overexpression. (f) *KNG1* knockdown in HepG2 cells transfected with pHBV plasmid and si-CTRL or si-*KNG1* for 48 h. (g) Levels of HBV DNA, HBV pgRNA, HBeAg, and HBsAg in HepG2 cells with *KNG1* knockdown. Error bars indicate SEM. *P*-value was determined using a two-tailed unpaired t-test. ∗*P* < 0.05; ∗∗*P* < 0.01; ∗∗∗*P* < 0.001.

To investigate the functions of *KNG1* in HBV infection, we first explored whether *KNG1* responds to HBV infection. By analyzing the gene expression data of primary human hepatocytes (PHHs) from GSE69590 before and after HBV infection, we found that *KNG1* was significantly upregulated after HBV infection compared with the PHH controls (Fig. 2a). In addition, we detected *KNG1* expression in HepG2-NTCP cells before and after HBV infection. Both qRT-PCR and Western blotting analysis results showed that HBV infection resulted in significantly increased *KNG1* expression (Fig. 2b and c).

We further studied the effect of *KNG1* on intracellular HBV replication in different liver cell lines. When comparing the cells transfected with the pHBV plasmid and control vector, *KNG1* overexpression significantly promoted the production of HBV in HepG2 cells (Fig. 2d and e). Such increased HBV replication levels were also observed in HepG2.2.15 and Huh7 cells transfected with *KNG1* overexpression plasmid (Supplementary Fig. S2a–S2d). Next, we down-regulated *KNG1* expression by siRNA targeting all transcripts of *KNG1*. There was a significant reduction in the amounts of various HBV markers, including HBV DNA, HBV pgRNA, HBeAg, and HBsAg in HepG2 cells with *KNG1* knock-down (Fig. 2f and g). To determine whether the role of *KNG1* in promoting HBV replication was universal, we performed the same experiments in the HepG2.2.15 and Huh7 cells and found that down-regulated *KNG1* expression effectively inhibited all of the above HBV markers (Supplementary Fig. S3a–S3d). Together, these results indicate that *KNG1* plays a key role in promoting HBV replication.

### Downregulation of IFNs by *KNG1* through MAVS inhibition

**Fig. 3:**
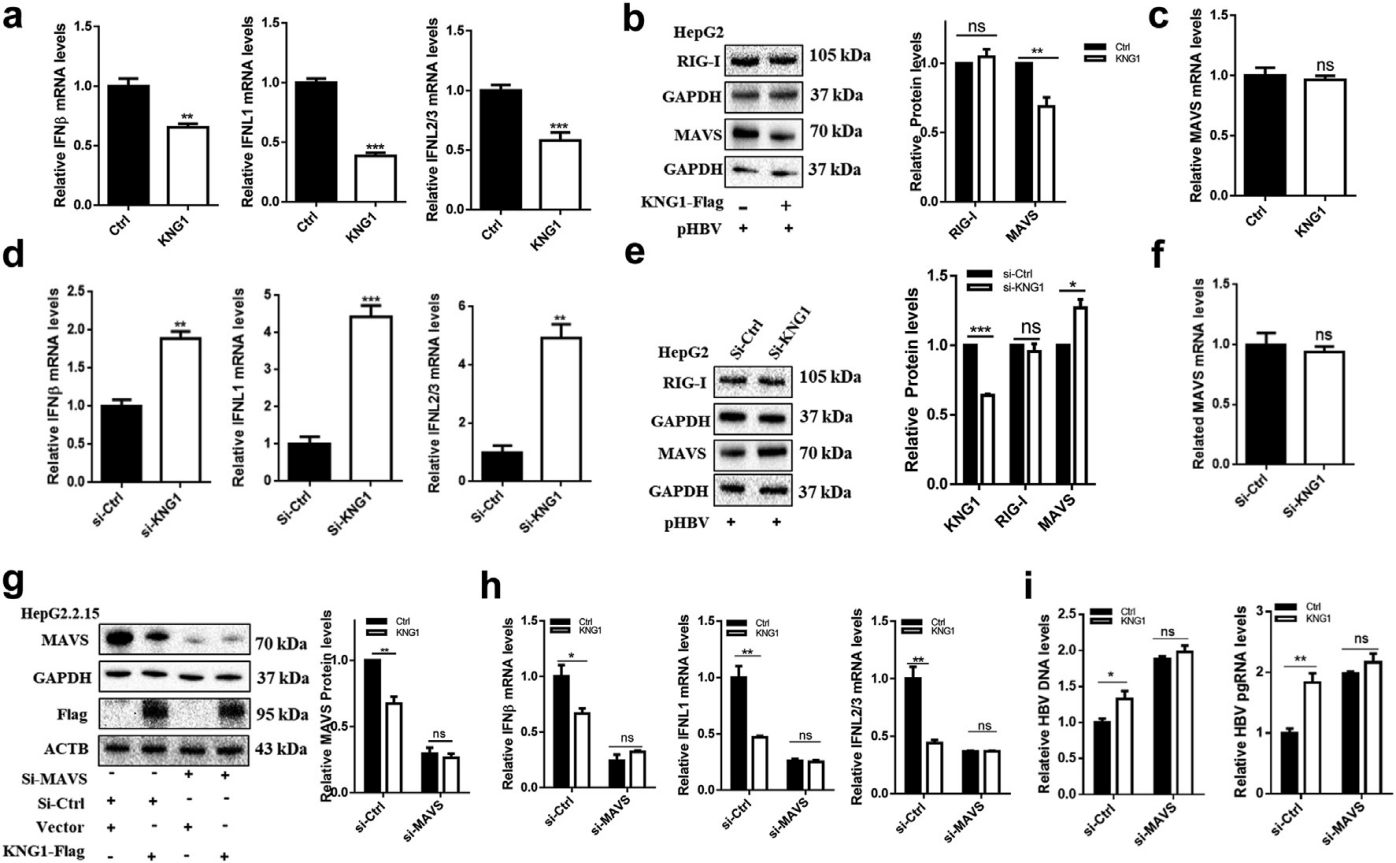
**Downregulation of IFNs by *KNG1* through MAVS inhibition.** (a) Effect of *KNG1* overexpression on types I and III IFNs in HepG2 cells. (b and c) Effect of *KNG1* overexpression on *MAVS* in HepG2 cells. HepG2 cells were transfected with pHBV and *KNG1*-Flag or vector plasmids for 48 h. The *MAVS* mRNA and protein levels were determined by qRT-PCR and Western blotting, respectively. (d) Effect of *KNG1* knockdown on types I and III IFNs in HepG2 cells. (e and f) Effect of *KNG1* knockdown on *MAVS* in HepG2 cells. HepG2 cells were transfected with pHBV plasmid and si-Ctrl or si-*KNG1* for 48 h. The *MAVS* mRNA and protein levels were determined by qRT-PCR and Western blotting. (g and h) Role of *MAVS* in *KNG1* regulating types I and III IFNs in HepG2.2.15 cells. HepG2.2.15 cells were transfected with control or *MAVS*-specific siRNAs together with KNG1 overexpression. (i) Levels of HBV DNA and HBV pgRNA in HepG2.2.15 cells. The mRNA and protein levels were determined by qRT-PCR and Western blotting. Error bars indicate SEM. *P*-value was determined using a two-tailed unpaired t-test. ∗*P* < 0.05; ∗∗*P* < 0.01; ∗∗∗*P* < 0.001.

Considering it is well known that interferons can inhibit HBV replication under different physiological conditions,^20^^,^^35^, ^36^, ^37^, ^38^ we next sought to determine whether *KNG1* affects the expression of IFNs as well. We found that overexpression of *KNG1* significantly reduced the expression of types I (*IFN-β*) and III IFNs (i.e., *IFNL1*, *IFNL2/3*) in HepG2 cells (Fig. 3a). To explore the potential inhibitory mechanism of *KNG1* on the expression of IFNs, two key upstream genes in the HBV-activated IFN pathway, *RIG-I* and *MAVS*,^20^^,^^23^ were measured by qRT-PCR and Western blotting. The results showed that *KNG1* had no significant effect on *RIG-I*, but it significantly down-regulated MAVS protein without affecting mRNA expression (Fig. 3b and c), which is consistent with the findings in HepG2 cells transfected with si-*KNG1* (Fig. 3d–f). Moreover, significant inhibitory effects of *KNG1* on MAVS and IFNs were also observed in HepG2.2.15 and Huh7 cells (Supplementary Figs. S4 and S5).

We then investigated whether *KNG1* inhibited types I and III IFNs via a MAVS-dependent pathway and found that in HepG2.2.15 cells with suppressed *MAVS* expression through siRNA, the ability of *KNG1* to inhibit the expression of IFNs and promote HBV replication was impaired (Fig. 3g–i). These results indicate that *KNG1* promotes HBV replication by affecting the post-transcriptional regulation of MAVS to inhibit IFNs expression.

### *KNG1* decreases IFNs through MAVS inhibition in an HSP90A-dependent manner

**Fig. 4:**
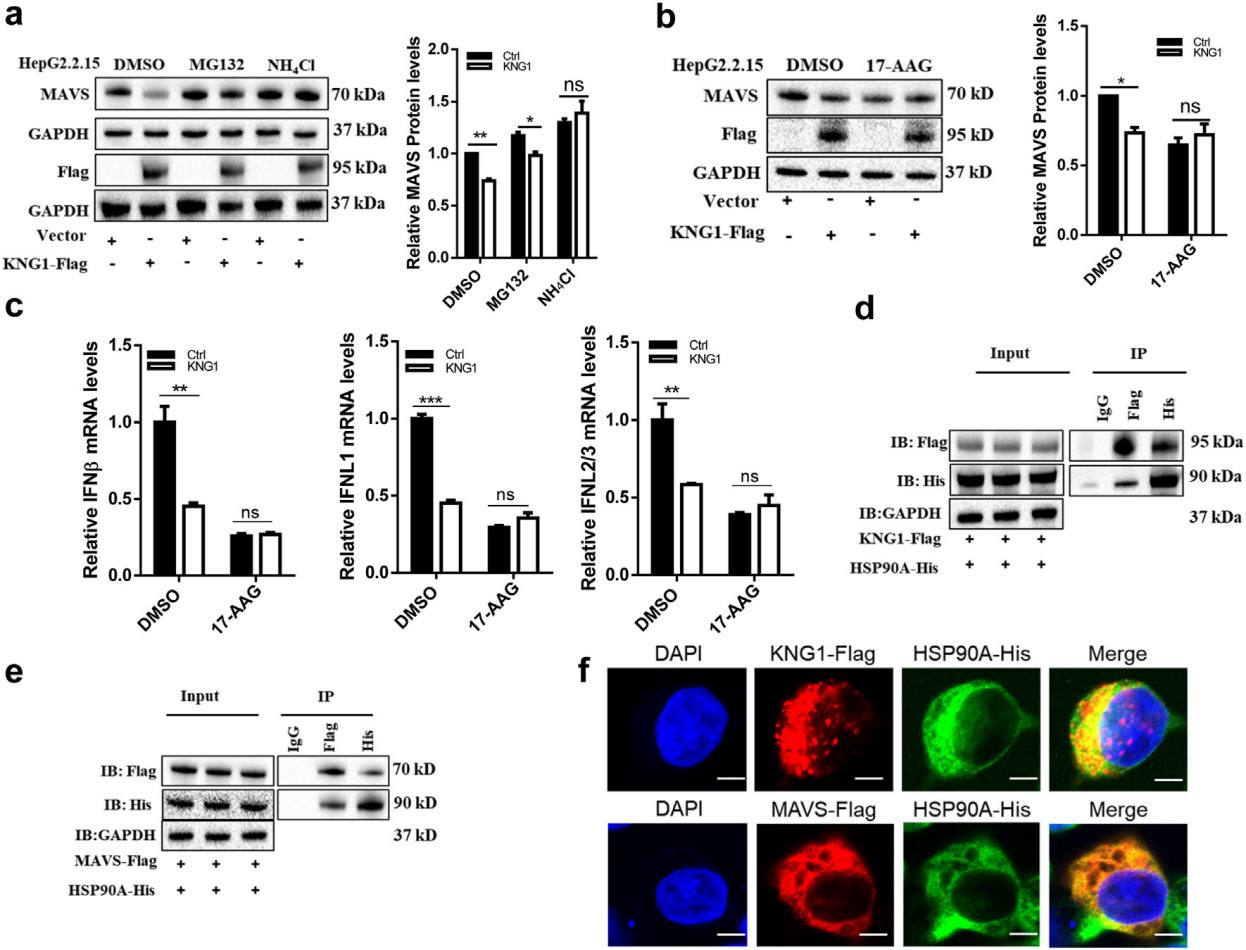
***KNG1* decreases IFNs through MAVS inhibition in HSP90A-dependent manner.** (a) Effect of *KNG1* on MAVS lysosomal degradation in HepG2.2.15 cells. HepG2.2.15 cells were treated with 20 mM NH_4_Cl or 10 μM MG132 for 6 h after KNG1-Flag plasmids were transfected. (b) Effect of 17-AAG on the regulation of *KNG1* on MAVS protein. HepG2.2.15 cells were treated with 0.5 μM 17-AAG for 24 h after *KNG1* overexpression. (c) Effect of 17-AAG on the regulation of *KNG1* on types I and III IFNs. Levels of IFNs were determined by qRT-PCR normalized to *GAPDH*. (d and e) KNG1 and MAVS interact with HSP90A in HepG2.2.15 cells. Cells were co-transfected with *HSP90A*-His along with *KNG1*-Flag (d) or *MAVS*-Flag plasmids (e), then immunoprecipitated and immunoblotted with indicated antibodies. (f) Colocalization of KNG1, MAVS, and HSP90A in HepG2.2.15 cells. Cells were co-transfected with *HSP90A*-His along with *KNG1*-Flag or *MAVS*-Flag plasmids. Nuclei were stained with DAPI. Scale bar = 5 μm. Error bars indicate SEM. *P*-value was determined using a two-tailed unpaired t-test. ∗*P* < 0.05; ∗∗*P* < 0.01; ∗∗∗*P* < 0.001.

There are two main protein degradation pathways in cells: the ubiquitin-proteasome pathway and the lysosome pathway. To determine which pathway was necessary for downregulating the MAVS protein by *KNG1*, lysosomal inhibitor NH_4_Cl or proteasome inhibitor MG132 was used to treat HepG2.2.15 cells transfected with a *KNG1*-expressed plasmid. Western blotting analysis showed that the lysosomal inhibitor NH_4_Cl but not proteasome inhibitor MG132 was capable of restoring the downregulation of MAVS protein by *KNG1* (Fig. 4a), suggesting that *KNG1* promotes the lysosomal degradation of MAVS.

Using co-immunoprecipitation experiments, we found that KNG1 interacted with MAVS (Supplementary Fig. S6a), however, the mechanism of how KNG1 affects MAVS remained unclear. To further determine how KNG1 was involved in MAVS degradation, we collected protein mixtures containing KNG1 and MAVS and then identified them by LC/MS. This led to the identification of four potential binding proteins: HSP90A, CKAP4, HSPA5, and EEF1G (Supplementary Fig. S6b). We decided to focus on heat shock protein 90A (HSP90A) because it is a well-known regulator of protein homeostasis. Indeed, after treating HepG2.2.15 cells with 17-AAG (a selective inhibitor of HSP90A), the inhibitory effect of *KNG1* on MAVS and types I and III IFNs was blocked (Fig. 4b and c), indicating that *KNG1* suppresses MAVS and types I and III IFNs in an HSP90A-dependent manner.

We then validated the interaction between KNG1 and HSP90A by Western blotting after co-immunoprecipitation of both proteins (Fig. 4d and e). In addition, an immunofluorescence assay confirmed that KNG1, MAVS, and HSP90A were partially colocalized together (Fig. 4f). Thus, we conclude that both KNG1 and MAVS can interact with HSP90A, but how *KNG1* decreases MAVS-mediated IFNs through HSP90A remains to be seen through further investigation.

### *KNG1* competitively blocks the binding ability of MAVS to HSP90A

**Fig. 5:**
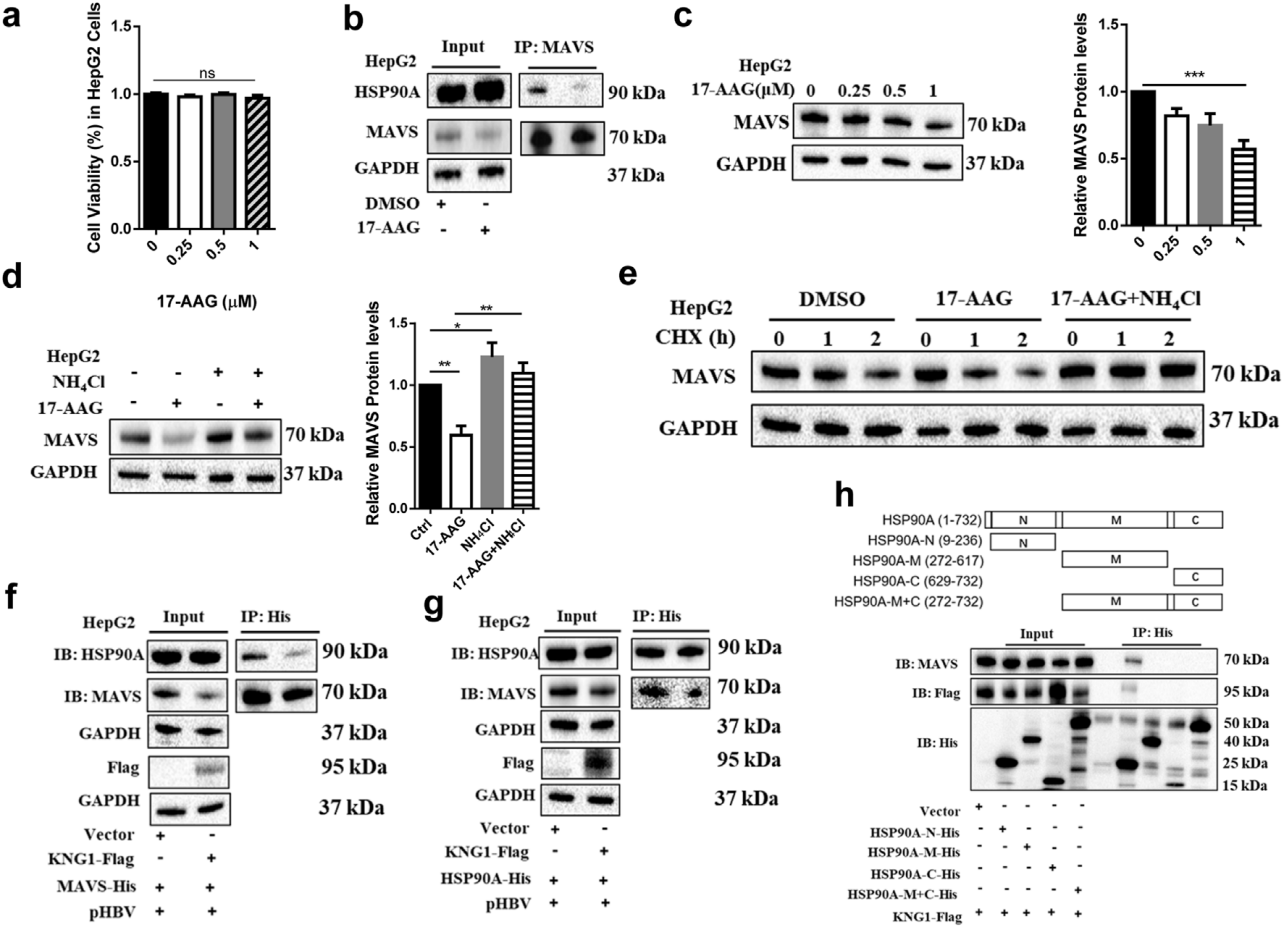
***KNG1* competitively blocks the binding of MAVS to HSP90A.** (a) Effect of 17-AAG on viability in HepG2 cells. Cells were treated with different concentrations of 17-AAG for 24 h, and viability assays were performed by Cell Counting Kit-8. (b) Effect of 17-AAG on the binding of MAVS to HSP90A in HepG2 cells. Cells were treated with 0.5 μM 17-AAG for 24 h before immunoprecipitation of MAVS. (c) Effect of 17-AAG on MAVS protein in HepG2 cells. Cells were treated with increased concentrations of 17-AAG for 24 h. (d) Effect of 17-AAG on MAVS lysosomal degradation in HepG2 cells. Cells were treated with or without NH_4_Cl (20 mM) before the addition of 17-AAG (0.5 μM). (e) Comparison of MAVS protein stability in HepG2 cells in cycloheximide (CHX) chase assay. Cells were treated with CHX (100 μg/μl) for increasing times (0–2 h) in the presence or absence of 17-AAG (0.5 μM). (f and g) Effect of *KNG1* on the binding of MAVS and HSP90A in HepG2 cells. Vector or *KNG1*-Flag plasmids were co-transfected into HepG2 cells with pHBV and *MAVS*-His or *HSP90A*-His plasmids. Cell lysates were incubated with anti-His antibody and Protein A + G agarose. (h) Identification of HSP90A domain responsible for binding to MAVS and KNG1. HepG2 cells were transfected with HSP90A truncation mutants (top). Cell lysates were incubated with anti-His agarose, and immunoprecipitated proteins were immunoblotted with indicated antibodies. Error bars indicate SEM. *P*-value was determined using a two-tailed unpaired t-test. ∗*P* < 0.05; ∗∗*P* < 0.01; ∗∗∗*P* < 0.001.

After excluding the toxic effects of 17-AAG in HepG2 cells, we found that 17-AAG prevented HSP90A from binding to MAVS in liver cells, which led to reduced levels of MAVS protein level in a dose-dependent manner (Fig. 5a–c). Given that *KNG1* promoted the lysosomal degradation of MAVS, we investigated whether HSP90A affects the lysosomal degradation of MAVS. The results showed that 17-AAG decreased MAVS protein in HepG2 cells, but the lysosomal inhibitor NH_4_Cl restored the level of MAVS (Fig. 5d). The stabilizing effect of HSP90A on MAVS protein was also observed in HepG2.2.15 and Huh7 cells (Supplementary Fig. S7a–S7h). In addition, we assessed MAVS protein stability in the presence or absence of 17-AAG by using the cycloheximide (CHX) chase assay and found that 17-AAG treatment significantly promoted the degradation of MAVS protein, whereas NH_4_Cl treatment prevented MAVS degradation (Fig. 5e; Supplementary Fig. S7i). These results suggest that functional abrogation of HSP90A triggers lysosome-mediated MAVS degradation.

We then investigated whether *KNG1* affects the lysosomal degradation of MAVS by regulating the binding of MAVS to HSP90A. The co-immunoprecipitation results showed that *KNG1* prevented the binding of MAVS to HSP90A (Fig. 5f). On the other hand, the binding abundance of HSP90A to MAVS was also significantly reduced after *KNG1* overexpression (Fig. 5g). Similarly, we carried out the same experiments in HepG2.2.15 and Huh7 cells and obtained the same results, i.e., *KNG1* inhibited the interaction of MAVS with HSP90A (Supplementary Fig. S8a–S8d).

The HSP90A protein contains three domains, of which the N-terminus is the ATP-binding domain, while the client protein tends to bind to the M-intermediate domain and the C-terminus domain.^39^ To identify the specific part corresponding to the combination of HSP90A with KNG1 and MAVS, we conducted co-immunoprecipitation assays using a series of truncated HSP90A structures and found that both MAVS and KNG1 were bound to the N-terminal domain of HSP90A (Fig. 5h). These results suggest that *KNG1* inhibits the binding ability of MAVS to HSP90A, resulting in MAVS degradation.

### *Kng1* promotes HBV replication by inhibiting *Mavs*-mediated IFN expression *in vivo*

**Fig. 6:**
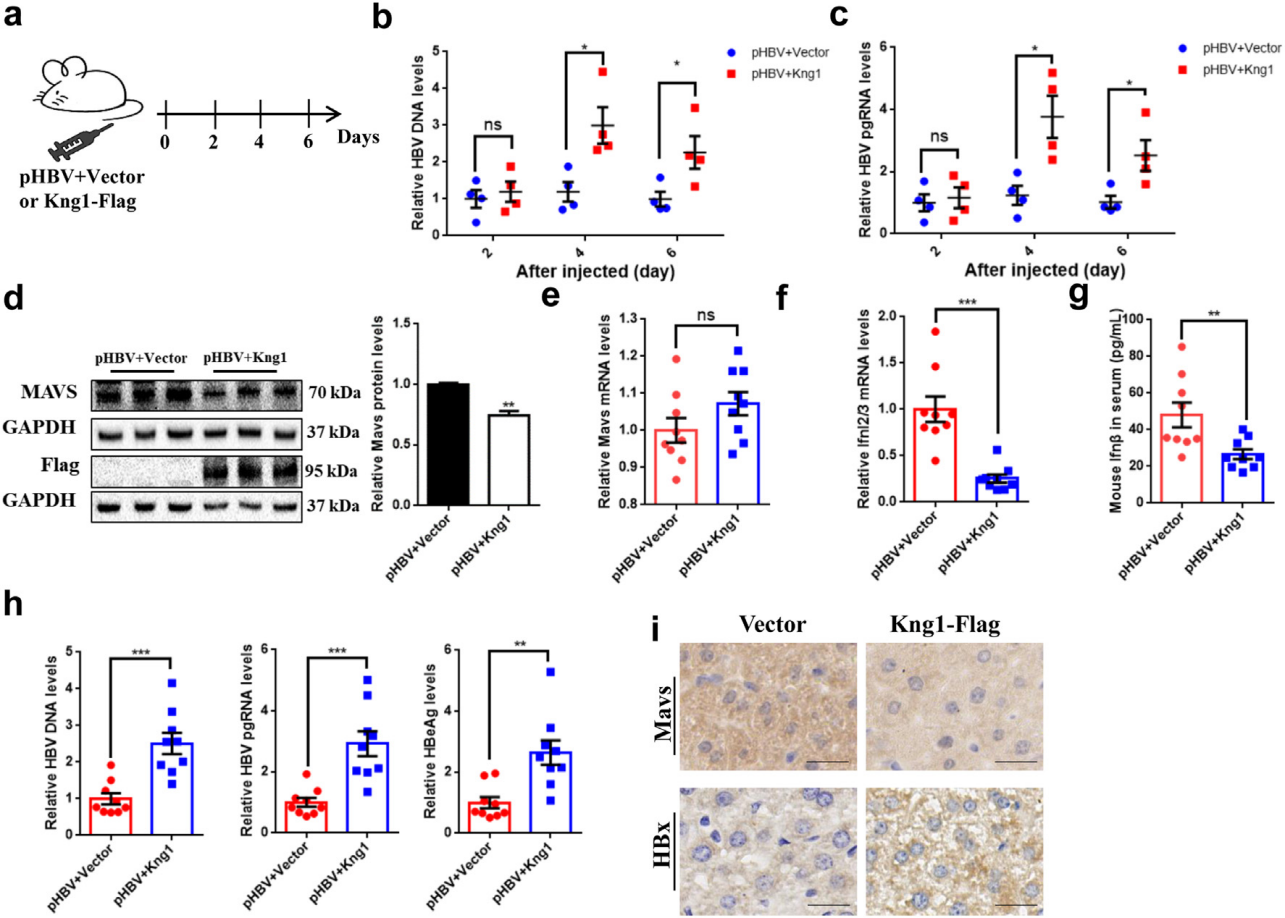
***Kng1* promotes HBV replication by inhibiting Mavs-mediated IFNs expression *in vivo*.** (a) C57BL/6 mice (n = 4) received HDI with pHBV (10 μg) along with vector (20 μg) or *Kng1*-Flag (20 μg) plasmids for 2, 4 and 6 days. (b and c) Levels of HBV DNA and HBV pgRNA in mice samples. (d and e) Western blotting analysis and qRT-PCR analysis of *Mavs* in mice liver samples. C57BL/6 mice (n = 9) received HDI with pHBV (10 μg) along with vector (20 μg) or *Kng1*-Flag (20 μg) plasmids for 4 days. (f) qRT-PCR analysis of *Ifnl2/3* expression in mice liver samples. (g) ELISA assay analysis of IFN-β in mice serum samples. (h) Levels of HBV DNA, HBV pgRNA, and HBeAg in mice liver samples. (i) The immunohistochemical staining of Mavs and HBx in mice liver. The data were the average of three independent experiments. Scale bar = 50 μm. Error bars indicate SEM. *P*-value was determined using a two-tailed unpaired t-test. ∗*P* < 0.05; ∗∗*P* < 0.01; ∗∗∗*P* < 0.001.

*In vitro* results showed that the *KNG1* promotes HBV replication by inhibiting types I and III IFNs expression, so we then evaluated *KNG1*-mediated IFNs inhibition in an animal model. The pHBV plasmid was hydrodynamically injected into the tail vein of mice together with the mouse homologous *Kng1* overexpression plasmid or its control vector. We then investigated their effects on days 2, 4, or 6 after injection (Fig. 6a). The results showed that compared with the control mice (n = 4), the HBV replication level in the *Kng1* group was significantly enhanced day 4 and 6 post-injection, and the replication level appeared to peak on day 4 (Fig. 6b and c). We then increased the number of experimental animals and studied *Kng1* function on day 4 after injection (n = 9). Western blotting analysis showed a higher expression of *Kng1* protein level in the livers of *Kng1*-overexpression mice compared with the control mice, which decreased Mavs protein level but did not affect its mRNA (Fig. 6d and e). Consistent with *in vitro* results, the expression of *Ifnl2/3* was significantly inhibited in the liver transfected with the *Kng1* plasmid, and ELISA assays also detected a significant down-regulation of IFN-β abundance in mice serum (Fig. 6f and g). Meanwhile, *Kng1* significantly promoted HBV replication (Fig. 6h). Immunohistochemical (IHC) staining yielded similar results and confirmed that *Kng1* significantly inhibited MAVS levels and increased HBx levels in the liver (Fig. 6i). Together, these data indicate that *Kng1* promotes HBV replication by suppressing IFN expression *in vivo*.

### Both *KNG1* overexpression and rs76438938-T reduce the therapeutic effects of IFN-α and IFN-λ1 in HBV infection

Both abovementioned *in vitro* and *in vivo* experiments have shown a significant inhibitory effect of *KNG1* on IFN expression. To determine the effect of KNG1 on the therapeutic effect of interferons, we assayed the expression level of classical IFN-stimulated genes (ISGs) in the cells treated with either IFN-α or IFN-λ1. After comparing the toxicities and antivirus effects of these two drugs under different concentrations, we finally decided to use 10 ng/ml of IFN-α and 100 ng/ml of IFN-λ1 for the following experiments (Supplementary Fig. S9a–S9d).

**Fig. 7:**
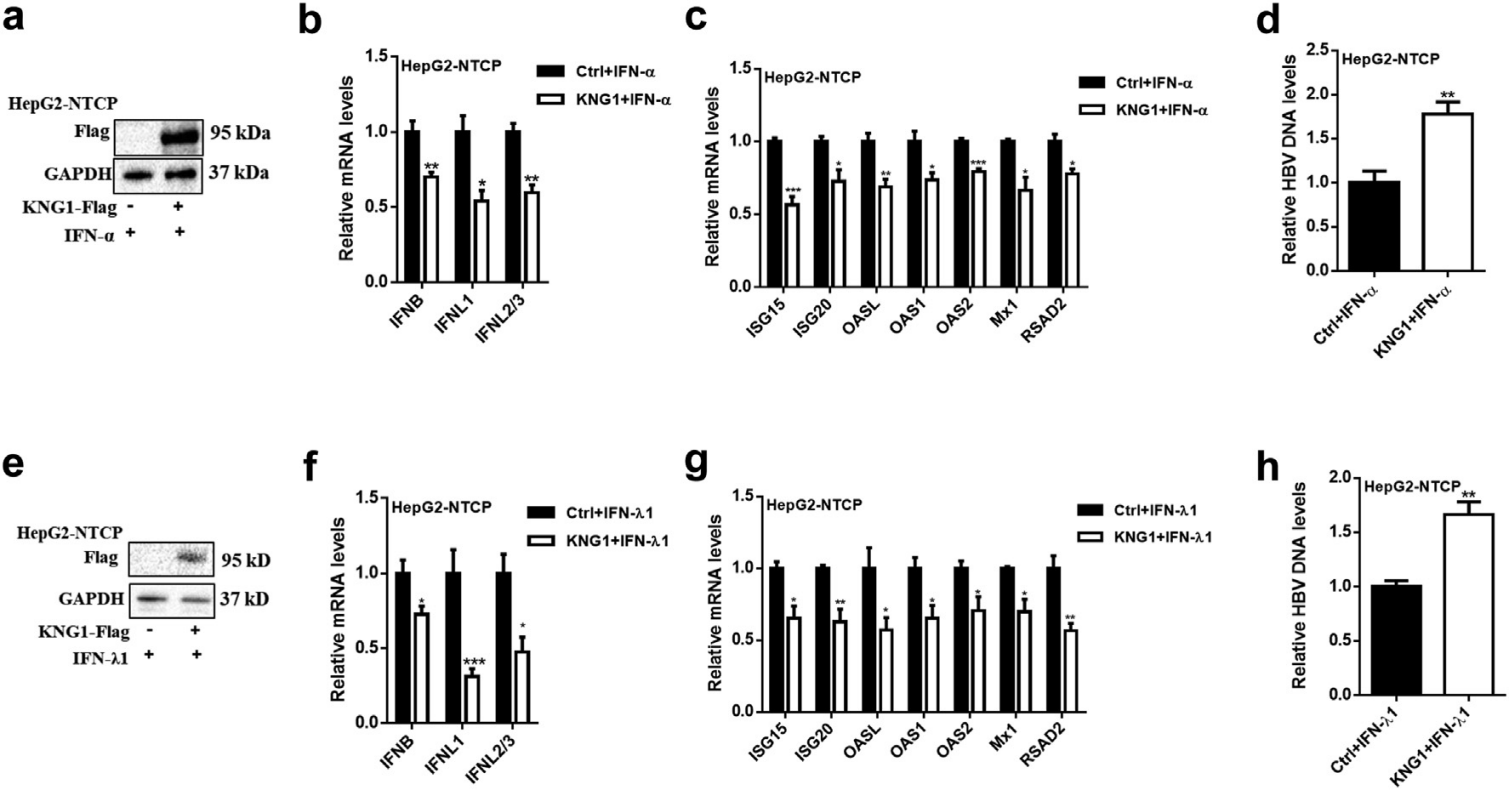
***KNG1* overexpression reduces the therapeutic effect of IFN-α and IFN-λ1 in HBV infection.** (a) *KNG1* overexpression in HepG2-NTCP cells under IFN-α treatment. HepG2-NTCP cells were transfected with vector or *KNG1*-Flag plasmid for 24 h before HBV infection and then treated with 10 ng/ml IFN-α for 48 h after infection. (b–d) qRT-PCR analysis of IFNs, ISGs and HBV DNA in HepG2-NTCP cells after IFN-α treatment. (e) *KNG1* overexpression in HepG2-NTCP cells under IFN-λ1 treatment. HepG2-NTCP cells were transfected with vector or *KNG1*-Flag plasmid for 24 h before HBV infection and then treated with 100 ng/ml IFN-λ1 for 48 h after infection. (f–h) qRT-PCR analysis of IFNs, ISGs and HBV DNA in HepG2-NTCP cells after IFN-λ1 treatment. Error bars indicate SEM. *P*-value was determined using a two-tailed unpaired t-test. ∗*P* < 0.05; ∗∗*P* < 0.01; ∗∗∗*P* < 0.001.

We first studied the role of *KNG1* regarding the treatment effect of IFN-α on HBV infection. The results showed that compared with the control HepG2-NTCP cells, *KNG1* overexpression significantly reduced the expression levels of IFN-α-activated endogenous IFNs and other typical ISGs (e.g., *ISG15*, *ISG20*, *OASL*, *OAS1*, *OAS2*, *Mx1*, and *RSAD2*), which eventually led to an increased HBV DNA level (Fig. 7a–d). Similarly, we obtained the same results in both HepG2.2.15 and Huh7 cell lines (Supplementary Figs. S10a–S10d and S11a–S11d). Further, we investigated the function of *KNG1* on the IFN-λ1 treatment and found that *KNG1* significantly inhibited the expression of IFN-λ1-activated interferons and multiple ISGs, leading to increased HBV replication levels (Fig. 7e–h, Supplementary Figs. S10e–S10h and S11e–S11h).

Since *LMWK* (i.e., the short form of *KNG1*) has a potential effect on HBV replication in cells, we explored the impact of wild-type (full-length) and mutant (truncated) LMWK on the therapeutic effect of IFN-α and IFN-λ1. Compared with the cells transfected with wild-type *LMWK* plasmid, we found weaker responses to both IFN-α and -λ1 treatment and significantly increased HBV DNA levels in HepG2-NTCP, HepG2.2.15, and Huh7 cells transfected with mutant *LMWK* plasmid (Supplementary Figs. S12–S14). Together, these results indicate that *KNG1* overexpression and risk allele rs76438938-T reduce the therapeutic effects of IFN-α and -λ1 in HBV infection.

## Discussion

**Fig. 8:**
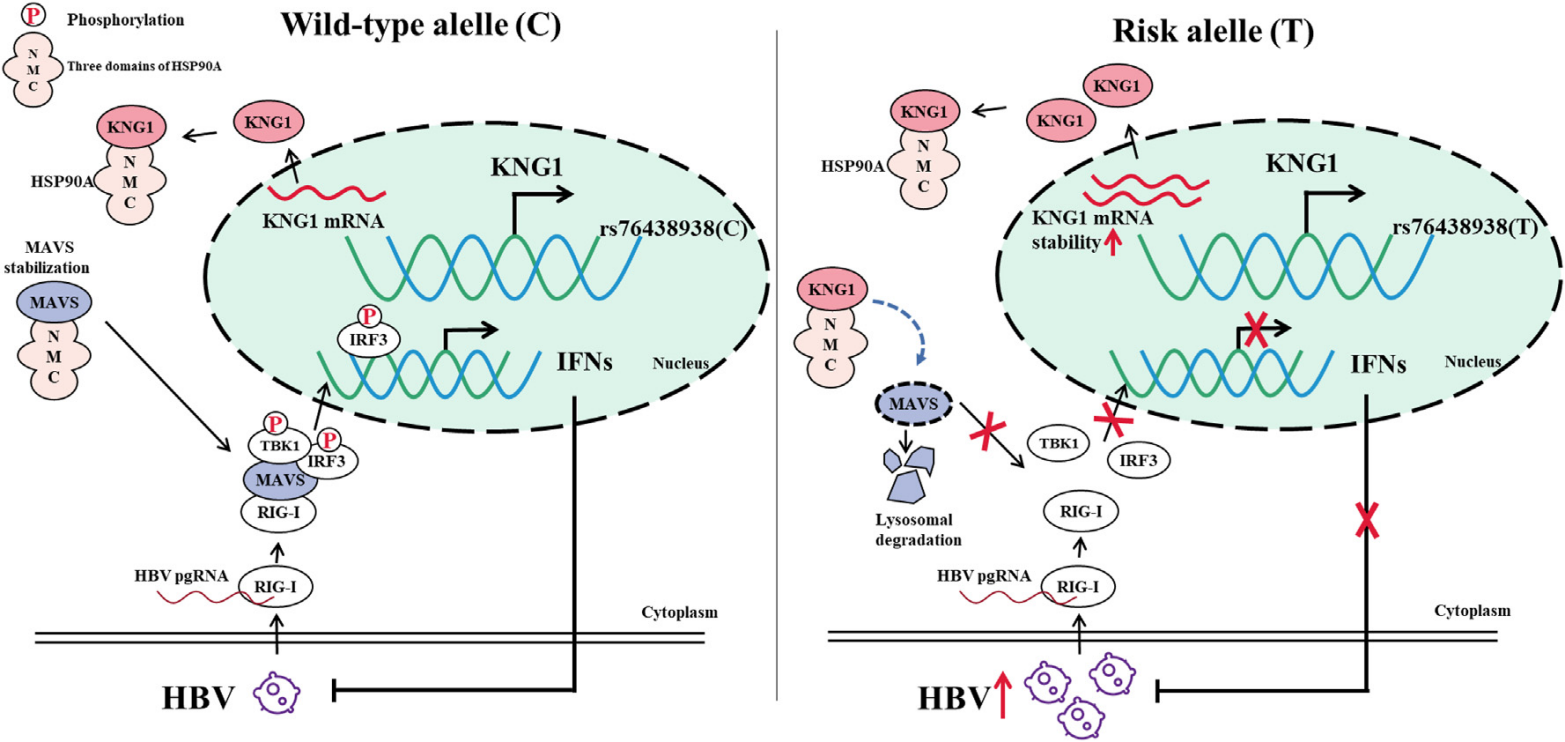
**Working model of rs76438938 in *KNG1* regulation of HBV infection.** Under normal circumstances, both KNG1 and MAVS bind to the N-terminal domain of molecular chaperone HSP90A, which can stabilize MAVS protein. After HBV infection, MAVS receives and transmits signals and mediates the expression of types I and III IFNs. When carrying the risk allele T, increased mRNA stability of *KNG1* leads to an increase in that protein, which competes with MAVS for binding to HSP90A. MAVS dissociated from HSP90A undergoes lysosomal degradation and cannot mediate the expression of types I and III IFNs, resulting in enhanced HBV replication, thereby increasing the risk of HBV infection.

Based on the results of genetic analysis, we found that SNP rs76438938, located in *KNG1*, was significantly associated with HBV infection. We further demonstrated that *KNG1* promotes HBV replication and inhibited types I and III IFN expression. Mechanistically, KNG1 protein competitively blocks the binding of MAVS to HSP90A, which triggers lysosomal degradation of MAVS, thereby suppressing types I and III IFN production and ultimately promoting HBV replication. Furthermore, we showed that rs76438938-T enhances the amount of *KNG1* by increasing its mRNA stability, which leads to an increased risk of HBV infection (Fig. 8).

By examining the clinical data of the subjects included in the study, we found that the SNP rs76438938-T in *KNG1* significantly increased the risk of HBV infection in carriers, and these individuals tended to have more severe disease stages. We also found that *KNG1* was significantly up-regulated in PHHs and HepG2-NTCP cells after HBV infection, suggesting that *KNG1* plays an important role in HBV replication. The frequency of SNP rs76438938 varies among different ethnic populations, with a frequency of 0.0125 in the Chinese Han population, 0.0273 in the European population, and 0.062 in the South Asian population. To better determine the function of the interested SNP, we constructed LMWK expression plasmids carrying rs76438938 C or T alleles and found that the risk T allele increased HBV replication. There are two main transcripts of *KNG1*: HMWK, which is the main product of *KNG1* that executes its biological function, and LMWK, which was almost undetectable in multiple hepatocytes and mouse liver samples and therefore likely has minimal biological function. The rs76438938-T allele promotes HBV replication by increasing *KNG1* mRNA stability. Given its location in the 3′-UTR of *HMWK*, rs76438938 could affect mRNA stability by regulating microRNAs (miRNAs) and/or RNA-binding proteins.^40^^,^^41^ However, such a suspicion remains to be investigated in future.

*KNG1* is an important part of the coagulation system and the kinin–kallikrein system (KKS).^42^ KKS activation mediates the production of the inflammatory mediator bradykinin (BK), thereby playing an important role in inflammatory diseases such as rheumatoid arthritis (RA).^43^ *KNG1* was reported to promote the progression of inflammation, and *KNG1* knockout mice show a lower inflammatory response.^44^^,^^45^ In this study, we found that *KNG1* significantly increased HBV replication and further demonstrated that *KNG1* decreased the expression of types I and III IFNs *in vitro* and *in vivo*. A recent study has also revealed a similar phenomenon, in which the increase of *KNG1* protein in patients infected with COVID-19 was accompanied by a decrease in immune system proteins.^46^

*MAVS* is a critical signal transduction platform for types I and III IFNs.^24^ It has been reported that *MAVS* can exert its antiviral effect of IFN-α and reduce the amounts of hepatitis B virus markers *in vitro* and *in vivo*.^38^ On the other hand, HBV could promote lactate production by activating the glycolytic pathway, and lactate binds directly to MAVS, preventing its aggregation and mitochondrial localization to escape the immune response.^47^ In this study, we found that overexpression of *KNG1* reduced MAVS protein without affecting its mRNA level, and the inhibition of *MAVS* expression blocked the effect of *KNG1* in inhibiting IFNs expression and promoting HBV replication. Furthermore, the lysosomal inhibitor NH_4_Cl prevented MAVS degradation, indicating that the regulation of MAVS by *KNG1* is through a lysosomal-dependent pathway. Jin et al. reported that tetherin can induce selective autophagy of MAVS to inhibit type I IFN signaling.^48^ After viral infection activates RLR, RNF34 promotes the polyubiquitination of MAVS, which is then recognized by NDP52 and degraded by autophagy.^49^ Thus, our findings reveal another regulator of MAVS that may influence the replication of other viruses.

*HSP90A* exerts multiple biological functions by regulating client protein maturation and stability. A functional study revealed that the dissociation of RIG-I-HSP90A may cause ubiquitination and proteasomal degradation of RIG-I and seriously compromise innate antiviral responses.^50^ Additionally, the inhibition of HSP90A by geldanamycin (GA) disrupts the NIK/HSP90A interaction and results in the autophagy-mediated degradation of NIK.^51^ In the current study, HSP90A was identified as the key protein that interacts with KNG1 and MAVS, which was confirmed by immunoprecipitation and immunofluorescence assays. Functionally, abrogation of HSP90A by 17-AAG blocked the inhibitory effect of *KNG1* on types I and III IFNs. According to the CHX chase assay, 17-AAG treatment accelerated the lysosomal-dependent degradation of MAVS. These results testify to the important role of HSP90A in the maturation of MAVS protein.

It is worth noting that the truncation experiment showed that both KNG1 and MAVS tended to bind to the N-terminal domain of HSP90A. These results indicate that KNG1 can inhibit the binding of MAVS to HSP90A by competitively interacting with the N-terminal domain of HSP90A. Interestingly, the activation of IRF3 requires binding to the N-terminal domain of HSP90A.^52^ There were some reports that the inhibition of the interaction of HSP90A with TBK1, IRF3, and Cdc37 impairs the innate immune response to viral infection.^53^^,^^54^ These results suggest another possibility that *KNG1* might regulate IFN expression by affecting the activity of IRF3, an idea that requires further research.

As an alternative therapy for hepatitis B infection, IFN-α can induce the expression of hundreds of ISGs to inhibit HBV infection.^55^ However, only a small proportion of patients benefit from IFN-α treatment because of concomitant immune dysfunction and toxicity.^56^ IFN-λ has been used in clinical trials for CHB treatment due to its acceptable efficacy and limited side effects.^57^^,^^58^ A newly reported study showed that in PHHs and HepaRG cells infected with HBV, IFN-β, IFN-λ1, and IFN-λ2 induce cccDNA deamination and degradation as efficiently as IFN-α.^59^ Considering the inhibitory effect of *KNG1* on intracellular interferon expression, we investigated the impact of *KNG1* on the response to both IFN-α and -λ1 treatment. We found that overexpressed *KNG1* inhibits the expression of endogenous IFNs and ISGs induced by the interferons α or λ1 treatments. It has been reported that most of the products encoded by ISGs are used to detect viral molecules and regulate signaling pathways, thereby increasing IFN production to control viral status.^60^ This is likely due to the fact that interferons α and λ1 treatment activate ISGs which then lead to increased interferon production. Recently, it was found that IFN-λ treatment leads to the activation of the *RIG-I-MAVS* pathway in HepG2.2.15 cells, and significantly up-regulates the expression of IFNs.^61^ Besides, we found that *LMWK* also has the potential to affect the efficacy of IFN-α and IFN-λ1 treatment, and the functional differences between wild-type and mutant LMWK proteins are thus worthy of further study. These results indicate that individuals carrying the risk allele rs76438938-T may require a high dose of interferons to cure HBV infection because of their reduced response to IFN-α or -λ1 treatments.

In sum, our results systematically elucidate that rs76438938 is a functional SNP and *KNG1* plays an important role in HBV infection and treatment. Further studies are needed to determine how rs76438938 affects *KNG1* to provide a feasible precision treatment strategy for rs76438938-T carriers.

## Contributors

BZ and YW conducted the experiments; BZ and XZ participated in data analysis and verification; WY and ZY managed the project; BZ, HH, ANL, ZY, and MDL participated in paper writing and editing; MDL conceived the study and was involved in every step of the project. All authors approved the paper as submitted.

## Data sharing statement

All data are available in the main text or the Supplementary Materials. The WES data is available in NCBI databank (PRJNA 553618).

## Declaration of interests

The authors report no biomedical financial interests or other potential conflicts of interest with the work reported in this paper.

## Appendix A Supplementary data

Supplementary Tables S1–S5

Supplementary Figs. S1–S14 Supplementary Figure 1. **Characterization of a functional variant rs76438938 in *KNG1* affecting HBV infection.** (a) Workflow for identifying SNPs associated with HBV infection. (b) The expression of *KNG1* in HepG2 and HepG2.2.15 cells. HepG2 cells were transfected with pcDNA3.1-LMWK as a positive control. (c) The expression of *KNG1* in mouse liver samples. (d) Sequencing analysis of L02 cell lines with CC or TT genotype. Supplementary Figure 2. **Overexpression of *KNG1* promotes HBV replication in HepG2.2.15 and Huh7 cells.** (a) Western blotting analysis of *KNG1* overexpression in HepG2.2.15 cells. Cells were transfected with vector or *KNG1*-Flag expression plasmids for 48 h. (b) Levels of HBV DNA, HBV pgRNA, HBeAg, and HBsAg in HepG2.2.15 cells. (c) Western blotting analysis of *KNG1* overexpression in Huh7 cells. Cells were transfected with pHBV and vector or *KNG1*-Flag expression plasmids for 48 h. (d) Levels of HBV DNA, HBV pgRNA, HBeAg, and HBsAg in Huh7 cells. Error bars indicate SEM. *P*-value was determined using a two-tailed unpaired t-test. ∗*P* < 0.05; ∗∗*P* < 0.01; ∗∗∗*P* < 0.001. Supplementary Figure 3. ***KNG1* knockdown inhibits HBV replication in HepG2.2.15 and Huh7 cells.** (a) Western blotting analysis of *KNG1* knockdown in HepG2.2.15 cells. Cells were transfected with si-CTRL or si-*KNG1* for 48 h. (b) Levels of HBV DNA, HBV pgRNA, HBeAg, and HBsAg in HepG2.2.15 cells. (c) Western blotting analysis of *KNG1* knockdown in Huh7 cells. Cells were transfected with pHBV plasmid and si-Ctrl or si-*KNG1* for 48 h. (d) Levels of HBV DNA, HBV pgRNA, HBeAg, and HBsAg in Huh7 cells. Error bars indicate SEM. *P*-value was determined using a two-tailed unpaired t-test. ∗*P* < 0.05; ∗∗*P* < 0.01; ∗∗∗*P* < 0.001. Supplementary Figure 4. **Downregulation of IFNs by *KNG1* through MAVS inhibition in HepG2.2.15 cells.** (a) Effect of *KNG1* overexpression on types I and III IFNs in HepG2.2.15 cells. (b and c) Effect of *KNG1* overexpression on MAVS in HepG2.2.15 cells. Cells were transfected with vector or *KNG1*-Flag plasmids for 48 h. The level of *MAVS* mRNA and protein were determined by qRT-PCR and Western blotting. (d) Effect of *KNG1* knockdown on IFNs in HepG2.2.15 cells. Cells transfected with si-Ctrl or si-*KNG1* for 48 h. The level of MAVS mRNA and protein were determined by qRT-PCR and Western blotting. (e and f) Effect of *KNG1* knockdown on MAVS in HepG2.2.15 cells. Error bars indicate SEM. *P*-value was determined using a two-tailed unpaired t-test. ∗*P* < 0.05; ∗∗*P* < 0.01; ∗∗∗*P* < 0.001. Supplementary Figure 5. **Downregulation of IFNs by *KNG1* through MAVS inhibition in Huh7 cells.** (a) Effect of *KNG1* overexpression on types I and III IFNs in Huh7 cells. (b and c) Effect of *KNG1* overexpression on MAVS in Huh7 cells. Cells were transfected with pHBV plasmid and vector or *KNG1*-Flag plasmids for 48 h. The level of *MAVS* mRNA and protein were determined by qRT-PCR and Western blotting. (d) Effect of *KNG1* knockdown on types I and III IFNs in Huh7 cells. (e and f) Effect of *KNG1* knockdown on MAVS in Huh7 cells. Cells transfected with pHBV plasmid and si-Ctrl or si-*KNG1* for 48 h. The level of MAVS mRNA and protein were determined by qRT-PCR and Western blotting. Error bars indicate SEM. *P*-value was determined using a two-tailed unpaired t-test. ∗*P* < 0.05; ∗∗*P* < 0.01; ∗∗∗*P* < 0.001. Supplementary Figure 6. **Identification of potential proteins that interact with KNG1 and MAVS.** (a) KNG1 interacts with MAVS in HepG2.2.15 cells. Cells were transfected with *KNG1*-Flag plasmids for 48 h. Cell lysates were incubated with anti-Flag agarose, and the immunoprecipitated proteins were immunoblotted with indicated antibodies. (b) Venn diagram (left) of proteins enriched by MAVS and KNG1. Table (right) lists the names of proteins. Supplementary Figure 7. **HSP90A is essential for maintaining intracellular MAVS protein level.** (a) Effect of 17-AAG on viability in HepG2.2.15 cells. Cells were treated with different concentrations of 17-AAG for 24 h, and viability assays were performed by Cell Counting Kit-8. (b) Effect of 17-AAG on the binding of MAVS to HSP90A in HepG2.2.15 cells. Cells were treated with 0.5 μM 17-AAG for 24 h before immunoprecipitation of MAVS. (c) Effect of 17-AAG on MAVS protein in HepG2.2.15 cells. Cells were treated with increased concentrations of 17-AAG for 24 h. (d) Effect of 17-AAG on MAVS lysosomal degradation in HepG2.2.15 cells. Cells were treated with or without NH_4_Cl (20 mM) before the addition of 17-AAG (0.5 μM). (e) Effect of 17-AAG on viability in Huh7 cells. (f) Effect of 17-AAG on the binding of MAVS to HSP90A in Huh7 cells. (g) Effect of 17-AAG on MAVS protein in Huh7 cells. (h) Effect of 17-AAG on MAVS lysosomal degradation in Huh7 cells. (i) Comparison of MAVS protein stability in HepG2.2.15 cells in cycloheximide (CHX) chase assay. Cells were treated with CHX (100 μg/μl) for increasing times (0–2 h) in the presence or absence of 17-AAG (0.5 μM). Supplementary Figure 8. **KNG1 competitively blocks the binding of MAVS to HSP90A in HepG2.2.15 and Huh7 cells.** (a and b) Effect of KNG1 on the binding of MAVS and HSP90A in HepG2.2.15 cells. Vector or *KNG1*-Flag plasmids were co-transfected into HepG2.2.15 cells with *MAVS*-His or *HSP90A*-His plasmids. Cell lysates were incubated with anti-His antibody and Protein A + G agarose. (c) Effect of KNG1 on the binding of MAVS and HSP90A in Huh7 cells. Vector or *KNG1*-Flag plasmids were co-transfected into Huh7 cells with pHBV plasmids. Cell lysates were incubated with anti-MAVS antibody and Protein A + G agarose. (d) Vector or *KNG1*-Flag plasmids were co-transfected into Huh7 cells with pHBV and HSP90A-His plasmids. Cell lysates were incubated with anti-His antibody and Protein A + G agarose. Supplementary Figure 9. **The toxicity and anti-virus effect of IFN-α/-λ1 in HepG2-NTCP cells.** (a–c) Effect of IFN-α and IFN-λ1 on the viability of HepG2-NTCP cells. Cells were treated with different concentrations of IFN-α or IFN-λ1 for 48 h after HBV infection, and viability assays were performed by cell counting kit-8. (b–d) Effect of IFN-α and IFN-λ1 on HBV DNA levels. HBV DNA levels were detected by qRT-PCR. Error bars indicate SEM. *P*-value was determined using a two-tailed unpaired t-test. ∗*P* < 0.05; ∗∗*P* < 0.01; ∗∗∗*P* < 0.001. Supplementary Figure 10. ***KNG1* overexpression reduces the therapeutic effect of IFN-α and -λ1 in HepG2.2.15 cells.** (a) *KNG1* overexpression in HepG2.2.15 cells. HepG2.2.15 cells were transfected with vector or *KNG1*-Flag plasmid for 24 h and then treated with 10 ng/ml IFN-α for 48 h. (b–d) qRT-PCR analysis of IFNs, ISGs and HBV DNA in HepG2.2.15 cells after IFN-α treatment. (e) *KNG1* overexpression in HepG2.2.15 cells. HepG2.2.15 cells were transfected with vector or *KNG1*-Flag plasmid for 24 h and then treated with 100 ng/ml IFN-λ1 for 48 h. (f–h) qRT-PCR analysis of IFNs, ISGs and HBV DNA in HepG2.2.15 cells after IFN-λ1 treatment. Error bars indicate SEM. *P*-value was determined using a two-tailed unpaired t-test. ∗*P* < 0.05; ∗∗*P* < 0.01; ∗∗∗*P* < 0.001. Supplementary Figure 11. ***KNG1* overexpression reduces the therapeutic effect of IFN-α and -λ1 in Huh7 cells.** (a) *KNG1* overexpression in Huh7 cells. Huh7 cells were transfected with pHBV and vector or *KNG1*-Flag plasmids for 24 h and then treated with 10 ng/ml IFN-α for 48 h. (b–d) qRT-PCR analysis of IFNs, ISGs and HBV DNA in Huh7 cells after IFN-α treatment. (e) *KNG1* overexpression in Huh7 cells. Huh7 cells were transfected with pHBV and vector or *KNG1*-Flag plasmids for 24 h and then treated with 100 ng/ml IFN-λ1 for 48 h. (f–h) qRT-PCR analysis of IFNs, ISGs and HBV DNA in Huh7 cells after IFN-λ1 treatment. Error bars indicate SEM. *P*-value was determined using a two-tailed unpaired t-test. ∗*P* < 0.05; ∗∗*P* < 0.01; ∗∗∗*P* < 0.001. Supplementary Figure 12. **SNP rs76438938-T reduces the therapeutic effect of IFN-α and -λ1 in HepG2-NTCP cells.** (a) *LMWK* with rs76438938 (C/T) overexpression in HepG2-NTCP cells under IFN-α treatment. HepG2-NTCP cells were transfected with *LMWK* plasmids carrying rs76438938-C or rs76438938-T for 24 h before HBV infection and then treated with 10 ng/ml IFN-α for 48 h after infection. (b–d) qRT-PCR analysis of IFNs, ISGs and HBV DNA in HepG2-NTCP cells after IFN-α treatment. (e) *LMWK* with rs76438938 (C/T) overexpression in HepG2-NTCP cells under IFN-λ1 treatment. HepG2-NTCP cells were transfected with *LMWK* plasmids carrying rs76438938-C or rs76438938-T for 24 h before HBV infection and then treated with 100 ng/ml IFN-λ1 for 48 h after infection. (f–h) qRT-PCR analysis of IFNs, ISGs and HBV DNA in HepG2-NTCP cells after IFN-λ1 treatment. Error bars indicate SEM. *P*-value was determined using a two-tailed unpaired t-test. ∗*P* < 0.05; ∗∗*P* < 0.01; ∗∗∗*P* < 0.001. Supplementary Figure 13. **SNP rs76438938-T reduces the therapeutic effect of IFN-α and -λ1 in HepG2.2.15 cells.** (a) *LMWK* with rs76438938 (C/T) overexpression in HepG2.2.15 cells under IFN-α treatment. HepG2.2.15 cells were transfected with *LMWK* plasmids carrying rs76438938-C or rs76438938-T for 24 h and then treated with 10 ng/ml IFN-α for 48 h after infection. (b–d) qRT-PCR analysis of IFNs, ISGs and HBV DNA in HepG2.2.15 cells after IFN-α treatment. (e) *LMWK* with rs76438938 (C/T) overexpression in HepG2.2.15 cells under IFN-λ1 treatment. HepG2.2.15 cells were transfected with *LMWK* plasmids carrying rs76438938-C or rs76438938-T for 24 h and then treated with 100 ng/ml IFN-λ1 for 48 h after infection. (f–h) qRT-PCR analysis of IFNs, ISGs and HBV DNA in HepG2.2.15 cells after IFN-λ1 treatment. Error bars indicate SEM. *P*-value was determined using a two-tailed unpaired t-test. ∗*P* < 0.05; ∗∗*P* < 0.01; ∗∗∗*P* < 0.001. Supplementary Figure 14. **SNP rs76438938-T reduces the therapeutic effect of IFN-α and -λ1 in Huh7 cells.** (a) *LMWK* with rs76438938 (C/T) overexpression in Huh7 cells under IFN-α treatment. Huh7 cells were transfected with pHBV and *LMWK* plasmids carrying rs76438938-C or rs76438938-T for 24 h and then treated with 10 ng/ml IFN-α for 48 h after infection. (b–d) qRT-PCR analysis of IFNs, ISGs and HBV DNA in Huh7 cells after IFN-α treatment. (e) *LMWK* with rs76438938 (C/T) overexpression in Huh7 cells under IFN-λ1 treatment. Huh7 cells were transfected pHBV and with *LMWK* plasmids carrying rs76438938-C or rs76438938-T for 24 h and then treated with 100 ng/ml IFN-λ1 for 48 h after infection. (f–h) qRT-PCR analysis of IFNs, ISGs and HBV DNA in Huh7 cells after IFN-λ1 treatment. Error bars indicate SEM. *P*-value was determined using a two-tailed unpaired t-test. ∗*P* < 0.05; ∗∗*P* < 0.01; ∗∗∗*P* < 0.001.

Supplemental Western blots

Reagent Validation files

## References

[1] Lai C.L., Ratziu V., Yuen M.F., Poynard T. (2003). Viral hepatitis B. Lancet.

[2] Perz J.F., Armstrong G.L., Farrington L.A., Hutin Y.J., Bell B.P. (2006). The contributions of hepatitis B virus and hepatitis C virus infections to cirrhosis and primary liver cancer worldwide. J Hepatol.

[3] Polaris Observatory Collaborators (2018). Global prevalence, treatment, and prevention of hepatitis B virus infection in 2016: a modelling study. Lancet Gastroenterol Hepatol.

[4] Ganem D., Prince A.M. (2004). Hepatitis B virus infection--natural history and clinical consequences. N Engl J Med.

[5] Davila S., Froeling F.E., Tan A. (2010). New genetic associations detected in a host response study to hepatitis B vaccine. Genes Immun.

[6] Wright T.L., Lau J.Y. (1993). Clinical aspects of hepatitis B virus infection. Lancet.

[7] Brouwer W.P., Sonneveld M.J., Tabak F. (2014). Polymorphisms of HLA-DP are associated with response to peginterferon in Caucasian patients with chronic hepatitis B. Aliment Pharmacol Ther.

[8] Mbarek H., Ochi H., Urabe Y. (2011). A genome-wide association study of chronic hepatitis B identified novel risk locus in a Japanese population. Hum Mol Genet.

[9] Nishida N., Sawai H., Matsuura K. (2012). Genome-wide association study confirming association of HLA-DP with protection against chronic hepatitis B and viral clearance in Japanese and Korean. PLoS One.

[10] Hu Z., Liu Y., Zhai X. (2013). New loci associated with chronic hepatitis B virus infection in Han Chinese. Nat Genet.

[11] Karra V.K., Gumma P.K., Chowdhury S.J. (2015). IL-18 polymorphisms in hepatitis B virus related liver disease. Cytokine.

[12] Huang X., Li H., Wang J. (2015). Genetic polymorphisms in toll-like receptor 3 gene are associated with the risk of hepatitis B virus-related liver diseases in a Chinese population. Gene.

[13] Geng P.L., Song L.X., An H., Huang J.Y., Li S., Zeng X.T. (2016). Toll-like receptor 3 is associated with the risk of HCV infection and HBV-related diseases. Medicine.

[14] Manolio T.A., Collins F.S., Cox N.J. (2009). Finding the missing heritability of complex diseases. Nature.

[15] Wieland S., Thimme R., Purcell R.H., Chisari F.V. (2004). Genomic analysis of the host response to hepatitis B virus infection. Proc Natl Acad Sci U S A.

[16] Fernandez M., Quiroga J.A., Carreno V. (2003). Hepatitis B virus downregulates the human interferon-inducible MxA promoter through direct interaction of precore/core proteins. J Gen Virol.

[17] Gordien E., Rosmorduc O., Peltekian C., Garreau F., Brechot C., Kremsdorf D. (2001). Inhibition of hepatitis B virus replication by the interferon-inducible MxA protein. J Virol.

[18] Lucifora J., Durantel D., Testoni B., Hantz O., Levrero M., Zoulim F. (2010). Control of hepatitis B virus replication by innate response of HepaRG cells. Hepatology.

[19] Shlomai A., Schwartz R.E., Ramanan V. (2014). Modeling host interactions with hepatitis B virus using primary and induced pluripotent stem cell-derived hepatocellular systems. Proc Natl Acad Sci U S A.

[20] Sato S., Li K., Kameyama T. (2015). The RNA sensor RIG-I dually functions as an innate sensor and direct antiviral factor for hepatitis B virus. Immunity.

[21] Sheppard P., Kindsvogel W., Xu W. (2003). IL-28, IL-29 and their class II cytokine receptor IL-28R. Nat Immunol.

[22] Onoguchi K., Yoneyama M., Takemura A. (2007). Viral infections activate types I and III interferon genes through a common mechanism. J Biol Chem.

[23] Kawai T., Takahashi K., Sato S. (2005). IPS-1, an adaptor triggering RIG-I- and Mda5-mediated type I interferon induction. Nat Immunol.

[24] Liu S., Cai X., Wu J. (2015). Phosphorylation of innate immune adaptor proteins MAVS, STING, and TRIF induces IRF3 activation. Science.

[25] Tao J., Su K., Yu C. (2015). Fine mapping analysis of HLA-DP/DQ gene clusters on chromosome 6 reveals multiple susceptibility loci for HBV infection. Amino Acids.

[26] Jiang X., Zhang B., Zhao J. (2019). Identification and characterization of SEC24D as a susceptibility gene for hepatitis B virus infection. Sci Rep.

[27] Li H., Durbin R. (2009). Fast and accurate short read alignment with Burrows-Wheeler transform. Bioinformatics.

[28] DePristo M.A., Banks E., Poplin R. (2011). A framework for variation discovery and genotyping using next-generation DNA sequencing data. Nat Genet.

[29] McKenna A., Hanna M., Banks E. (2010). The genome analysis toolkit: a MapReduce framework for analyzing next-generation DNA sequencing data. Genome Res.

[30] Wang K., Li M., Hakonarson H. (2010). ANNOVAR: functional annotation of genetic variants from high-throughput sequencing data. Nucleic Acids Res.

[31] Schaid D.J., Rowland C. (1998). Use of parents, sibs, and unrelated controls for detection of associations between genetic markers and disease. Am J Hum Genet.

[32] Purcell S., Neale B., Todd-Brown K. (2007). PLINK: a tool set for whole-genome association and population-based linkage analyses. Am J Hum Genet.

[33] Komor A.C., Zhao K.T., Packer M.S. (2017). Improved base excision repair inhibition and bacteriophage Mu Gam protein yields C:G-to-T:A base editors with higher efficiency and product purity. Sci Adv.

[34] Iwamoto M., Watashi K., Tsukuda S. (2014). Evaluation and identification of hepatitis B virus entry inhibitors using HepG2 cells overexpressing a membrane transporter NTCP. Biochem Biophys Res Commun.

[35] Li Y., Si L., Zhai Y. (2016). Genome-wide association study identifies 8p21.3 associated with persistent hepatitis B virus infection among Chinese. Nat Commun.

[36] Zhang Z., Filzmayer C., Ni Y. (2018). Hepatitis D virus replication is sensed by MDA5 and induces IFN-beta/lambda responses in hepatocytes. J Hepatol.

[37] Xu F., Song H., Xiao Q. (2019). Type III interferon-induced CBFbeta inhibits HBV replication by hijacking HBx. Cell Mol Immunol.

[38] Li T., Yang X., Li W. (2021). ADAR1 stimulation by IFN-alpha downregulates the expression of MAVS via RNA editing to regulate the anti-HBV response. Mol Ther.

[39] Young J.C., Obermann W.M., Hartl F.U. (1998). Specific binding of tetratricopeptide repeat proteins to the C-terminal 12-kDa domain of hsp90. J Biol Chem.

[40] Li F., Zhao H., Su M. (2019). HnRNP-F regulates EMT in bladder cancer by mediating the stabilization of Snail1 mRNA by binding to its 3′ UTR. EBioMedicine.

[41] Bartel D.P. (2009). MicroRNAs: target recognition and regulatory functions. Cell.

[42] Colman R.W., Schmaier A.H. (1997). Contact system: a vascular biology modulator with anticoagulant, profibrinolytic, antiadhesive, and proinflammatory attributes. Blood.

[43] Sainz I.M., Uknis A.B., Isordia-Salas I., Dela Cadena R.A., Pixley R.A., Colman R.W. (2004). Interactions between bradykinin (BK) and cell adhesion molecule (CAM) expression in peptidoglycan-polysaccharide (PG-PS)-induced arthritis. FASEB J.

[44] Langhauser F., Gob E., Kraft P. (2012). Kininogen deficiency protects from ischemic neurodegeneration in mice by reducing thrombosis, blood-brain barrier damage, and inflammation. Blood.

[45] Shi K., Chen X., Xie B. (2018). Celastrol alleviates chronic obstructive pulmonary disease by inhibiting cellular inflammation induced by cigarette smoke via the Ednrb/Kng1 signaling pathway. Front Pharmacol.

[46] Geyer P.E., Arend F.M., Doll S. (2021). High-resolution serum proteome trajectories in COVID-19 reveal patient-specific seroconversion. EMBO Mol Med.

[47] Zhou L., He R., Fang P. (2021). Hepatitis B virus rigs the cellular metabolome to avoid innate immune recognition. Nat Commun.

[48] Jin S., Tian S., Luo M. (2017). Tetherin suppresses type I interferon signaling by targeting MAVS for NDP52-mediated selective autophagic degradation in human cells. Mol Cell.

[49] He X., Zhu Y., Zhang Y. (2019). RNF34 functions in immunity and selective mitophagy by targeting MAVS for autophagic degradation. EMBO J.

[50] Matsumiya T., Imaizumi T., Yoshida H., Satoh K., Topham M.K., Stafforini D.M. (2009). The levels of retinoic acid-inducible gene I are regulated by heat shock protein 90-alpha. J Immunol.

[51] Qing G., Yan P., Qu Z., Liu H., Xiao G. (2007). Hsp90 regulates processing of NF-kappa B2 p100 involving protection of NF-kappa B-inducing kinase (NIK) from autophagy-mediated degradation. Cell Res.

[52] Yang K., Shi H., Qi R. (2006). Hsp90 regulates activation of interferon regulatory factor 3 and TBK-1 stabilization in Sendai virus-infected cells. Mol Biol Cell.

[53] Liu X., Main D., Ma Y., He B. (2018). Herpes simplex virus 1 inhibits TANK-binding kinase 1 through formation of the Us11-Hsp90 complex. J Virol.

[54] Lee M.N., Roy M., Ong S.E. (2013). Identification of regulators of the innate immune response to cytosolic DNA and retroviral infection by an integrative approach. Nat Immunol.

[55] Kwon H., Lok A.S. (2011). Hepatitis B therapy. Nat Rev Gastroenterol Hepatol.

[56] Ji H.F. (2011). Vitamin D levels may explain the racial differences in response rates to antiviral therapy for chronic hepatitis C. Hepatology.

[57] Phillips S., Mistry S., Riva A. (2017). Peg-interferon lambda treatment induces robust innate and adaptive immunity in chronic hepatitis B patients. Front Immunol.

[58] Chan H.L.Y., Ahn S.H., Chang T.T. (2016). Peginterferon lambda for the treatment of HBeAg-positive chronic hepatitis B: a randomized phase 2b study (LIRA-B). J Hepatol.

[59] Bockmann J.H., Stadler D., Xia Y. (2019). Comparative analysis of the antiviral effects mediated by type I and III interferons in hepatitis B virus-infected hepatocytes. J Infect Dis.

[60] Sadler A.J., Williams B.R. (2008). Interferon-inducible antiviral effectors. Nat Rev Immunol.

[61] Makjaroen J., Somparn P., Hodge K., Poomipak W., Hirankarn N., Pisitkun T. (2018). Comprehensive proteomics identification of IFN-lambda3-regulated antiviral proteins in HBV-transfected cells. Mol Cell Proteomics.

