# Supplementary material for: An HBV susceptibility variant of KNG1 modulates the therapeutic effects of interferons α and λ1 in HBV infection by promoting MAVS lysosomal degradation
Source: eBioMedicine. 2023 Jul 11;94:104694. doi: 10.1016/j.ebiom.2023.104694 (PMC10435766; doi:10.1016/j.ebiom.2023.104694)
Supplement: Supplementary Tables S1–S5 [file mmc1.docx]

Supplementary Table 1. Demographic characteristics of samples used in this study.

| Characteristic | CHBVI | Sib-Controls |
| --- | --- | --- |
| Sample size | 300 | 300 |
| Age (years) | 39.5±11.3 | 39.3±11.7 |
| Female (%) | 110 (36.7%) | 169 (56.3%) |
| Male (%) | 190 (63.3%) | 131 (43.7%) |
| HBV-DNA (*10^8^ copies/mL) | 6.6±46.1 | —— |
| HBsAg (*10^3^ IU/mL） | 1.2±1.8 | —— |
| ALT (U/L) | 80.9±127.2 | 22.0±15.2 |
| Albumin (g/L) | 44.0±7.2 | 44.7±3.7 |
| TBIL (µmol/L) | 44.3±97.3 | 11.4±5.7 |

ALT = alanine aminotransferase; CHBVI = chronic hepatitis B virus infection; TBIL = total bilirubin

Supplementary Table 2. Primers used in this study.

| GAPDH-F | CGACAGTCAGCCGCATCTT |
| --- | --- |
| GAPDH-R | CCGTTGACTCCGACCTTCA |
| KNG1-F | CTCGGCTGTGTGCATCCTAT |
| KNG1-R | CCTGTCTTTGGGCCCGTTTT |
| HMWK-F | TCAACCACTGGGAATGATCTCAC |
| HMWK-R | CGCTCTTCATCTTGTGCAGG |
| LMWK-F | AGCCTAGATTGCAACGCTGA |
| LMWK-R | GGACCTTAGGTGACTAGTTGTT |
| HBV DNA-F | GAGTGTGGATTCGCACTCC |
| HBV DNA-R | GAGGCGAGGGAGTTCTTCT |
| HBV pgRNA-F | CACCCTAGCAGCCATGGAAA |
| HBV pgRNA-R | GCTCCTCTGCCGATCCATAC |
| ISG15-F | CGCAGATCACCCAGAAGATCG |
| ISG15-R | TTCGTCGCATTTGTCCACCA |
| ISG20-F | CTCGTTGCAGCCTCGTGAA |
| ISG20-R | CGGGTTCTGTAATCGGTGATCTC |
| OASL-F | CTGATGCAGGAACTGTATAGCAC |
| OASL-R | CACAGCGTCTAGCACCTCTT |
| OAS1-F | TGTCCAAGGTGGTAAAGGGTG |
| OAS1-R | CCGGCGATTTAACTGATCCTG |
| OAS2-F | ACGTGACATCCTCGATAAAACTG |
| OAS2-R | GAACCCATCAAGGGACTTCTG |
| Mx1-F | GGTGGTCCCCAGTAATGTGG |
| Mx1-R | CGTCAAGATTCCGATGGTCCT |
| RSAD2-F | TTGGACATTCTCGCTATCTCCT |
| RSAD2-R | AGTGCTTTGATCTGTTCCGTC |
| MAVS-F | GGACGAAGTGGCCTCTGTCTA |
| MAVS-R | CATGGGGTAACTTGGCTCCTT |
| IFNB-F | TCTCCTGTTGTGCTTCTCCAC |
| IFNB-R | GCCTCCCATTCAATTGCCAC |
| IFNL1-F | CGCCTTGGAAGAGTCACTCA |
| IFNL1-R | GAAGCCTCAGGTCCCAATTC |
| IFNL2/3-F | AGTTCCGGGCCTGTATCCAG |
| IFNL2/3-R | GAGCCGGTACAGCCAATGGT |
| Mouse Gapdh-F | AGGTCGGTGTGAACGGATTTG |
| Mouse Gapdh-R | TGTAGACCATGTAGTTGAGGTCA |
| Mouse Mavs-F | CTGCCTCACAGCTAGTGACC |
| Mouse Mavs-R | CCGGCGCTGGAGATTATTG |
| Mouse Kng1-F | CTGCTGACTTTAACACAGGGAG |
| Mouse Kng1-R | GGTTGCCACTTTTTACCCCAG |
| Mouse Ifnl2/3-F | AAGAACCCAAGCTGACCCTG |
| Mouse Ifnl2/3-R | AAGCAGCCTCTTCTCGATGG |

Supplementary Table 3. A list of siRNAs used in this study.

| si-Ctrl | UUCUCCGAACGUGUCACGU |
| --- | --- |
| si-KNG1 | GCAGAGAAUAACGCAACUU |
| si-MAVS | CCACCUUGAUGCCUGUGAA |

Supplementary Table 4. Summary of three nonsense mutants in this study.

| Gene | SNP ID | Chr.: position | Minor allele | Major allele | MAF | Genotype | DFAM | | Case/Control | | Function |
| --- | --- | --- | --- | --- | --- | --- | --- | --- | --- | --- | --- |
|  |  |  |  |  |  |  | CHISQ | P | OR | P |  |
| KNG1 | rs76438938 | chr3:186743675 | T | C | 0.0125 | 0/15/585 | 7.364 | 0.0067 | 4.666 | 0.0209 | Stopgain |
| MAP3K10 | rs773840600 | chr19:40205180 | T | G | 0.0108 | 0/13/587 | 6.231 | 0.0126 | 0.1841 | 0.0305 | Stopgain |
| OR5AR1 | rs11228710 | chr11:56663740 | C | T | 0.2217 | 22/222/356 | 4.983 | 0.0256 | 1.441 | 0.0155 | Stopgain |

Supplementary Table 5. The genotype of family individuals carrying the risk allele T of SNP rs76438938.

| Family ID# | 35 | | 40 | | 253 | | 260 | | 277 | | 463 | | 646 | | 710 | | 794 | | 968 | | 2336 | | 2490 | | 2499 | |
| --- | --- | --- | --- | --- | --- | --- | --- | --- | --- | --- | --- | --- | --- | --- | --- | --- | --- | --- | --- | --- | --- | --- | --- | --- | --- | --- |
| HBV infected | √ | x | √ | x | √ | x | √ | x | √ | x | √ | x | √ | x | √ | x | √ | x | √ | x | √ | x | √ | x | √ | x |
| Genotype | **CT** | CC | **CT** | CC | **CT** | CC | **CT** | CC | **CT** | CC | **CT** | CC | **CT** | CC | **CT** | CC | **CT** | CC | **CT** | **CT** | **CT** | **CT** | CC | **CT** | **CT** | CC |
| Gender | F | F | F | F | M | F | M | M | F | F | F | F | F | F | M | M | M | F | M | F | F | F | F | F | M | F |
| Stage | CHB |  | HC |  | CHB |  | HC |  | CHB |  | CHB |  | HC |  | Carrier |  | HC |  | HC |  | CHB |  | Carrier |  | Carrier |  |

CHB= Chronic hepatitis B; HC=Hepatitis cirrhosis
