# Supplementary material for: An HBV susceptibility variant of KNG1 modulates the therapeutic effects of interferons α and λ1 in HBV infection by promoting MAVS lysosomal degradation
Source: eBioMedicine. 2023 Jul 11;94:104694. doi: 10.1016/j.ebiom.2023.104694 (PMC10435766; doi:10.1016/j.ebiom.2023.104694)
Supplement: Supplementary Figs. S1–S14 — Supplementary Figure 1. Characterization of a functional variant rs76438938 in KNG1 affecting HBV infection. (a) Workflow for identifying SNPs associated with HBV infection. (b) The expression of KNG1 in HepG2 and HepG2.2.15 cells. HepG2 cells were transfected with pcDNA3.1-LMWK as a positive control. (c) The expression of KNG1 in mouse liver samples. (d) Sequencing analysis of L02 cell lines with CC or TT genotype. Supplementary Figure 2. Overexpression of KNG1 promotes HBV replication in HepG2.2.15 and Huh7 cells. (a) Western blotting analysis of KNG1 overexpression in HepG2.2.15 cells. Cells were transfected with vector or KNG1-Flag expression plasmids for 48 h. (b) Levels of HBV DNA, HBV pgRNA, HBeAg, and HBsAg in HepG2.2.15 cells. (c) Western blotting analysis of KNG1 overexpression in Huh7 cells. Cells were transfected with pHBV and vector or KNG1-Flag expression plasmids for 48 h. (d) Levels of HBV DNA, HBV pgRNA, HBeAg, and HBsAg in Huh7 cells. Error bars indicate SEM. P-value was determined using a two-tailed unpaired t-test. ∗P < 0.05; ∗∗P < 0.01; ∗∗∗P < 0.001. Supplementary Figure 3. KNG1 knockdown inhibits HBV replication in HepG2.2.15 and Huh7 cells. (a) Western blotting analysis of KNG1 knockdown in HepG2.2.15 cells. Cells were transfected with si-CTRL or si-KNG1 for 48 h. (b) Levels of HBV DNA, HBV pgRNA, HBeAg, and HBsAg in HepG2.2.15 cells. (c) Western blotting analysis of KNG1 knockdown in Huh7 cells. Cells were transfected with pHBV plasmid and si-Ctrl or si-KNG1 for 48 h. (d) Levels of HBV DNA, HBV pgRNA, HBeAg, and HBsAg in Huh7 cells. Error bars indicate SEM. P-value was determined using a two-tailed unpaired t-test. ∗P < 0.05; ∗∗P < 0.01; ∗∗∗P < 0.001. Supplementary Figure 4. Downregulation of IFNs by KNG1 through MAVS inhibition in HepG2.2.15 cells. (a) Effect of KNG1 overexpression on types I and III IFNs in HepG2.2.15 cells. (b and c) Effect of KNG1 overexpression on MAVS in HepG2.2.15 cells. Cells were transfected with vector or KNG1-Flag [file mmc2.pptx]

## Slide 1
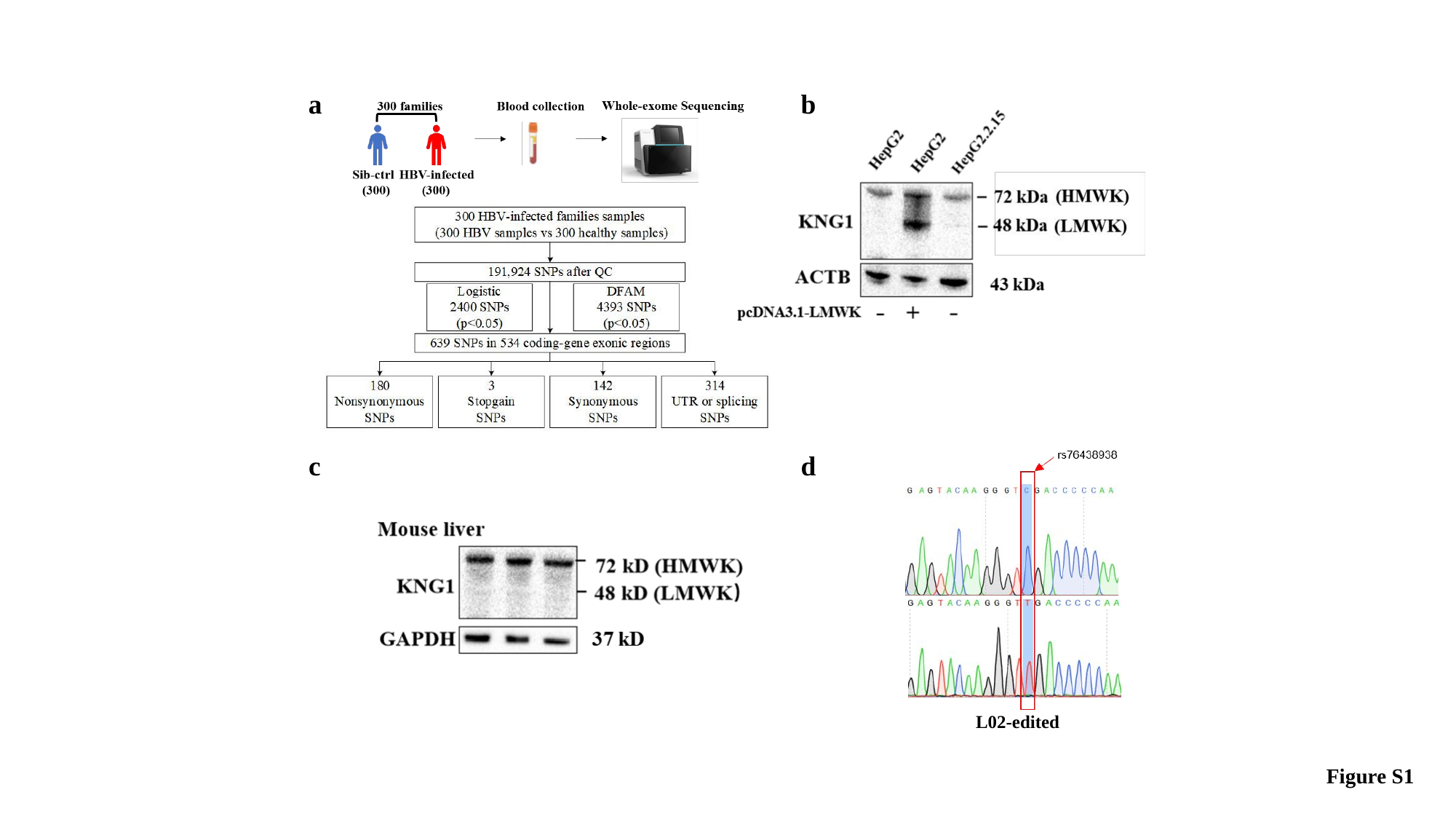

a
b
c
d
L02-edited
Figure S1

## Slide 2
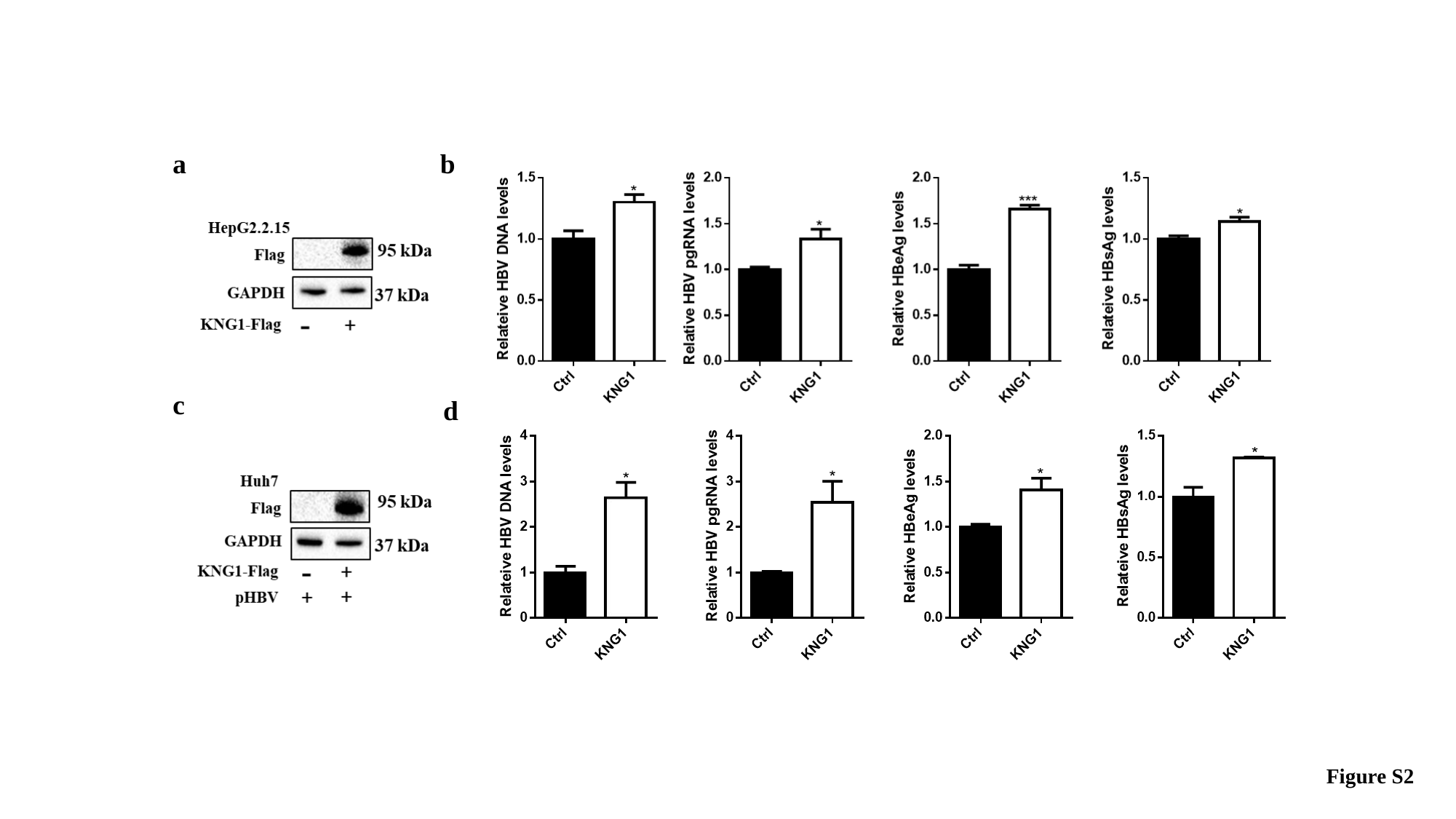

a
b
c
d
Figure S2

## Slide 3
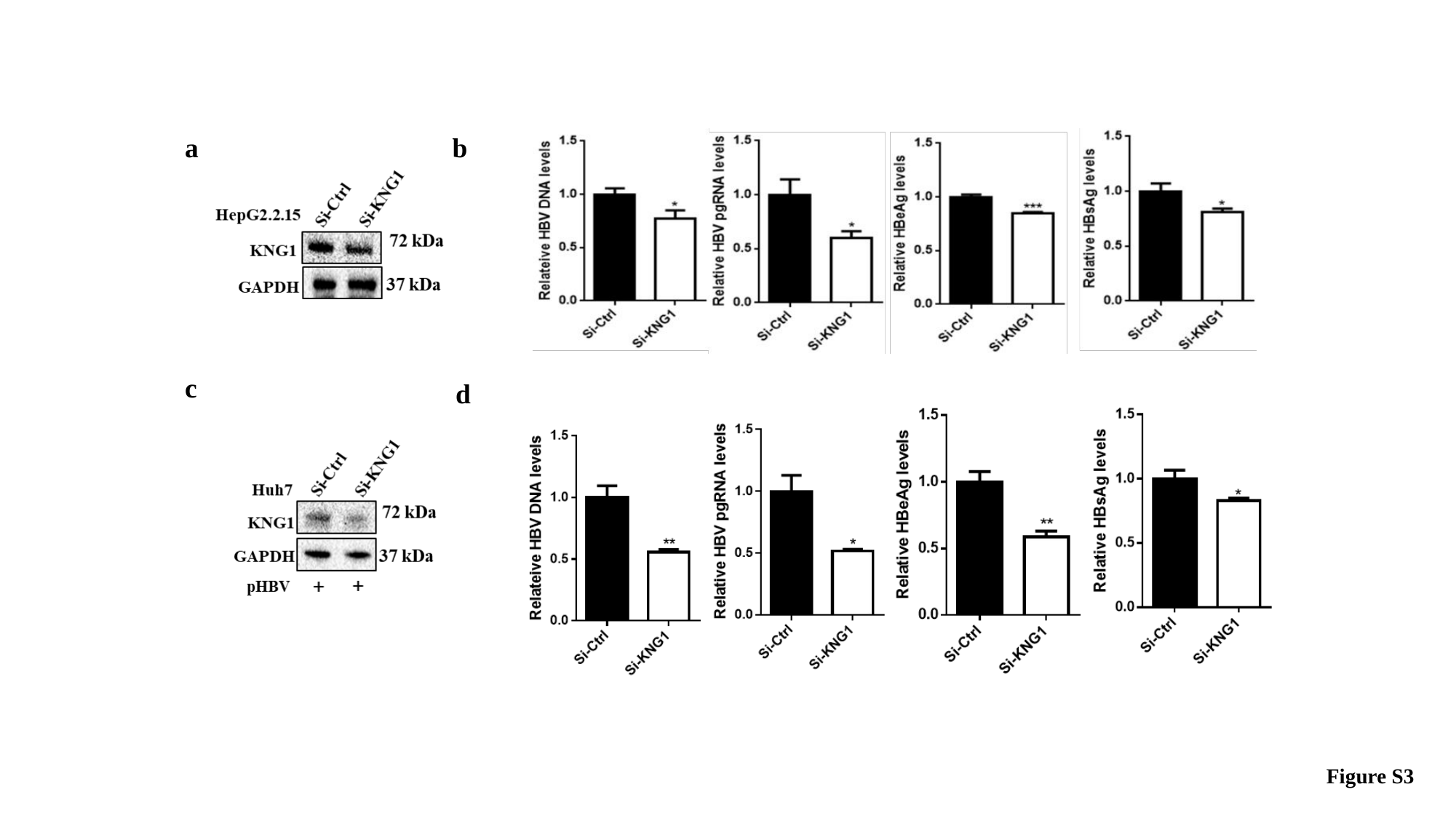

a
b
c
d
Figure S3

## Slide 4
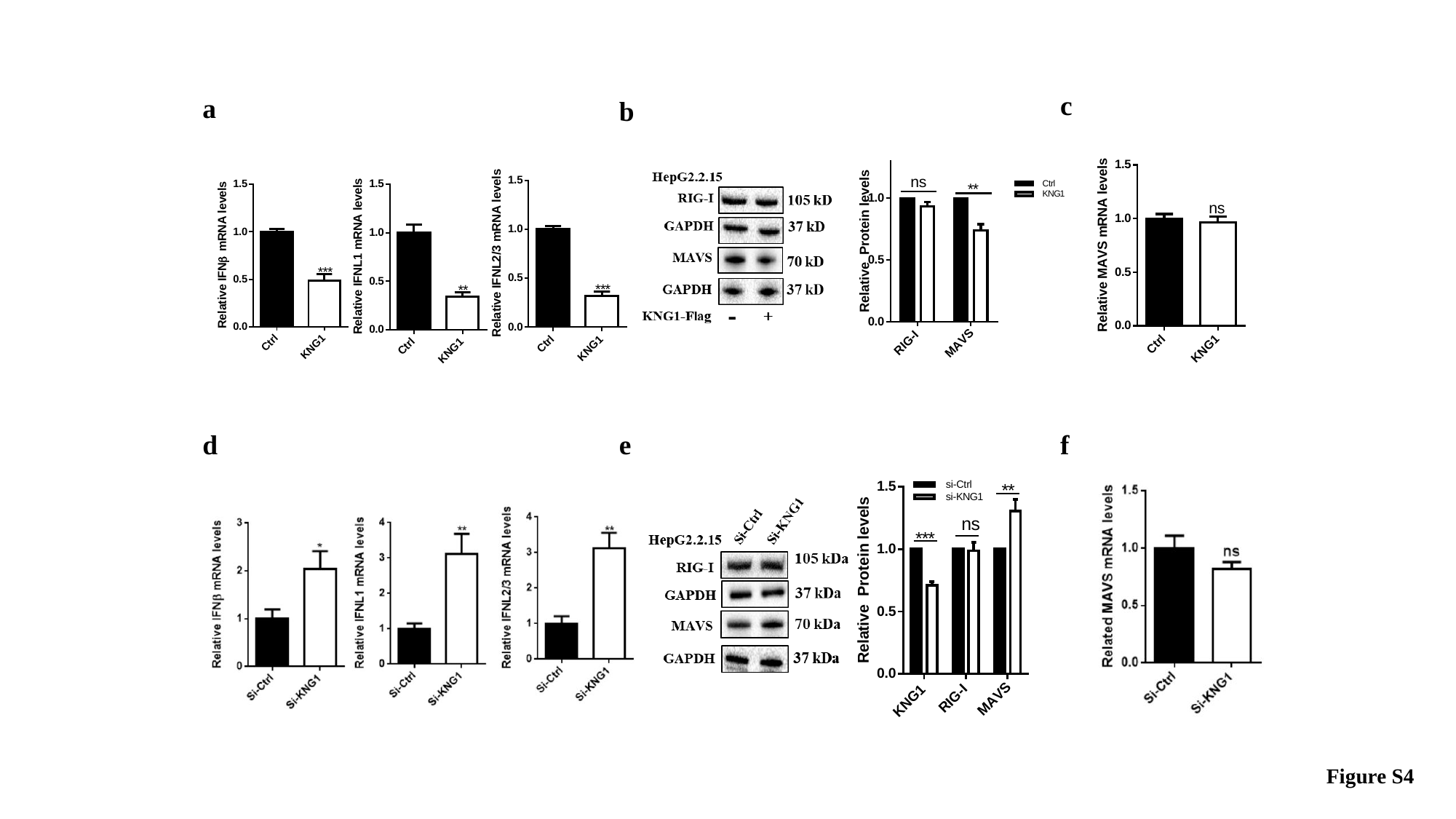

c
a
b
d
e
f
Figure S4

## Slide 5
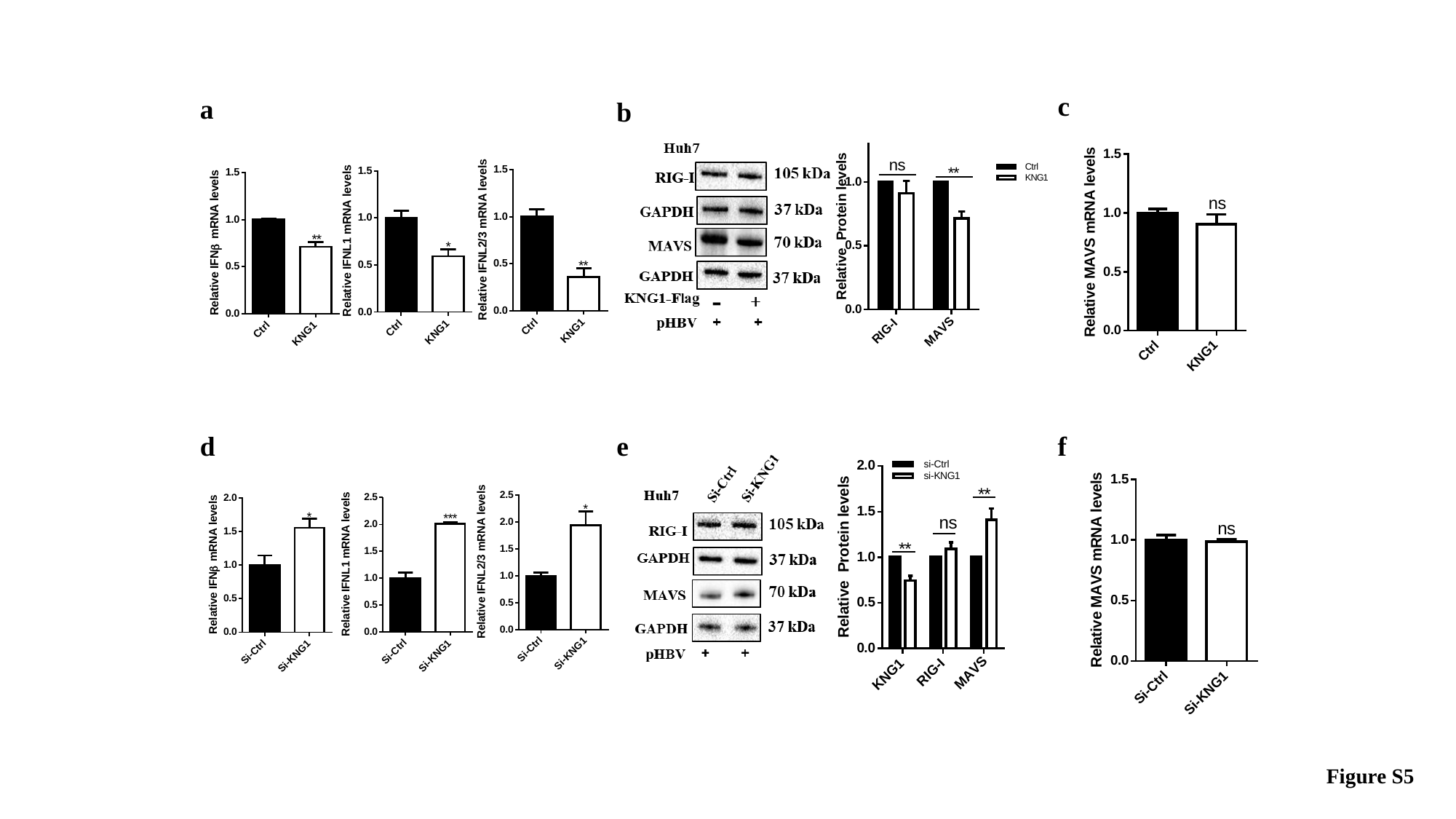

c
a
b
d
e
f
Figure S5

## Slide 6
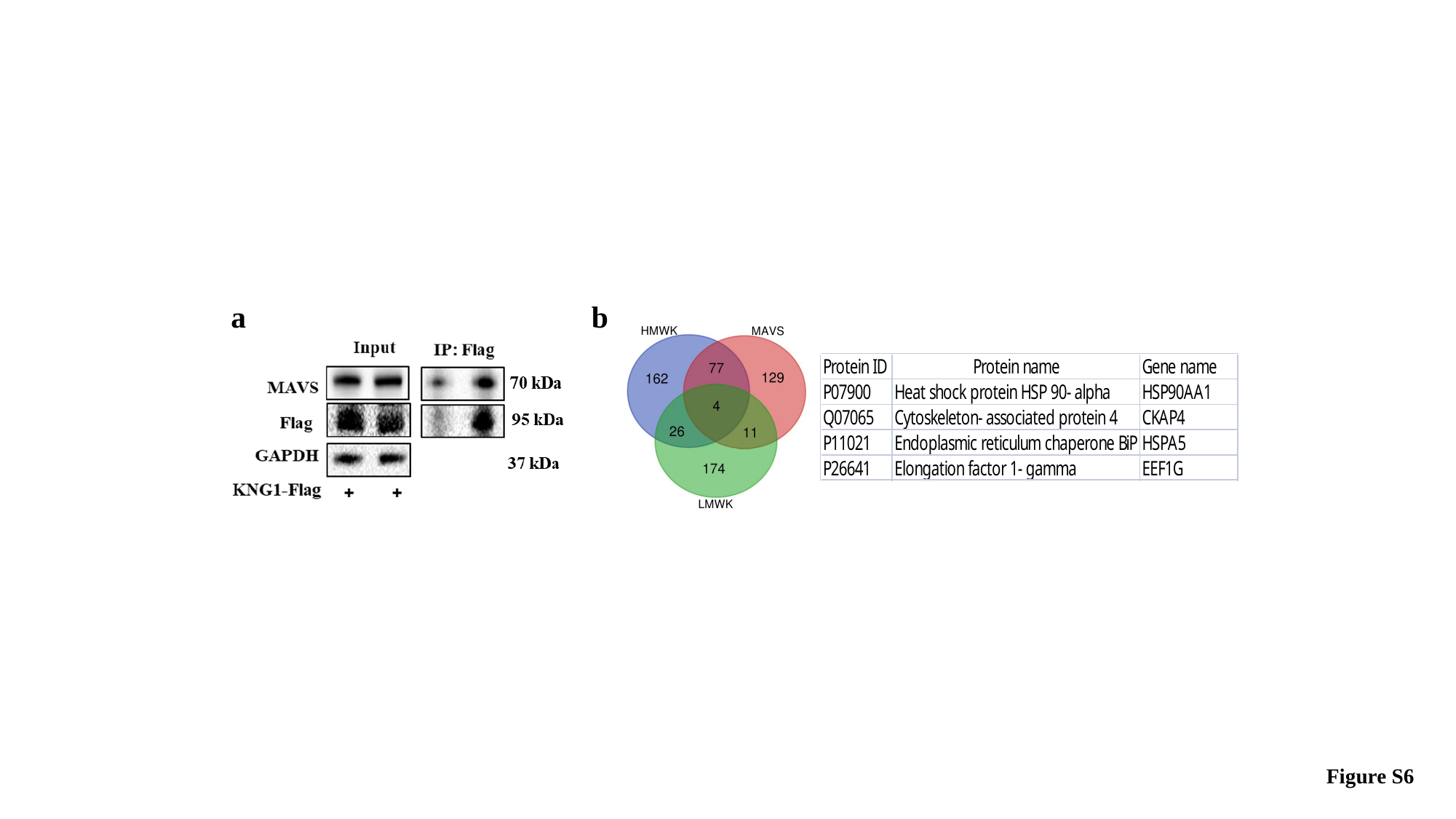

a
b
Figure S6

## Slide 7
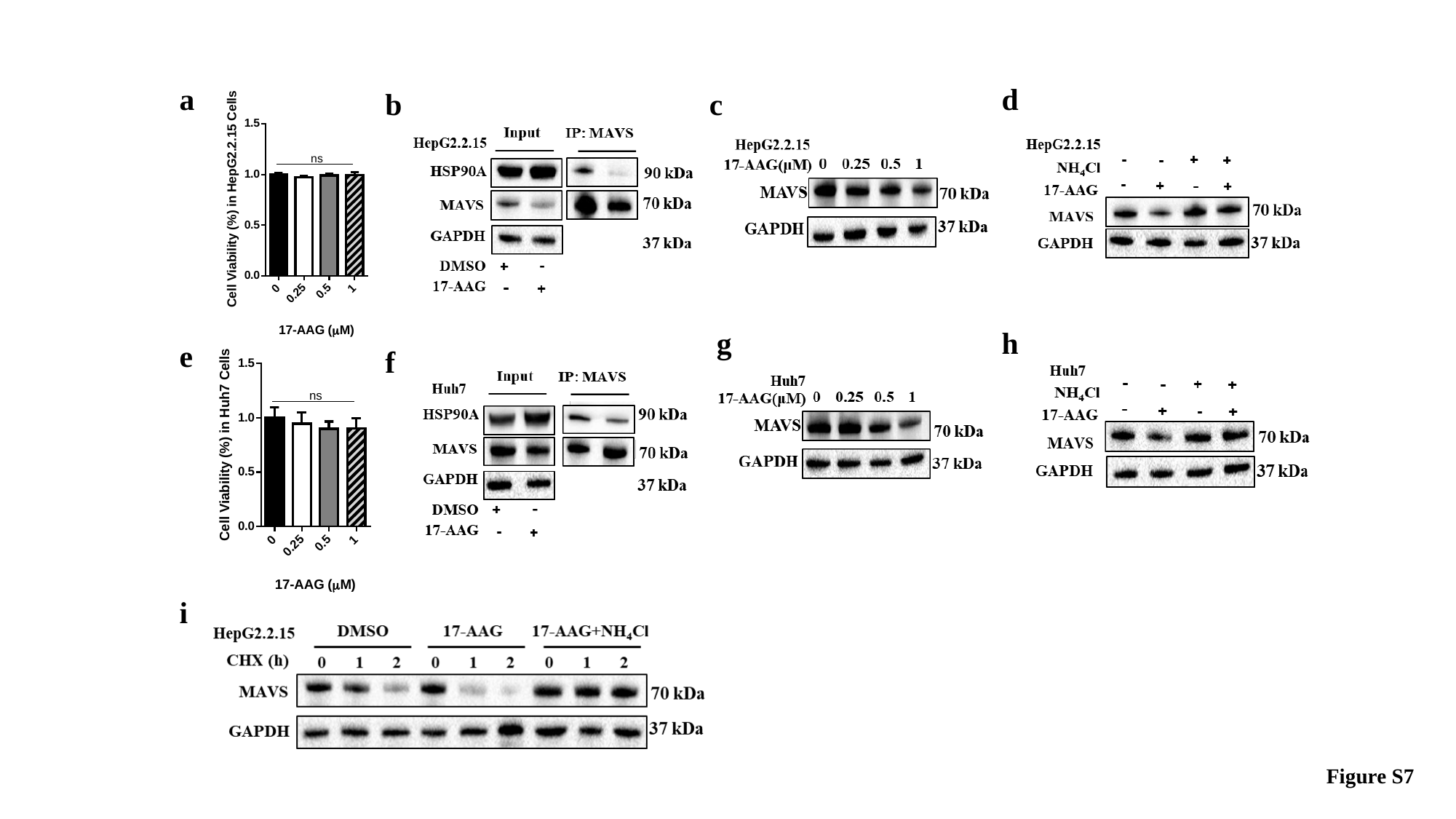

a
d
b
c
g
h
e
f
i
Figure S7

## Slide 8
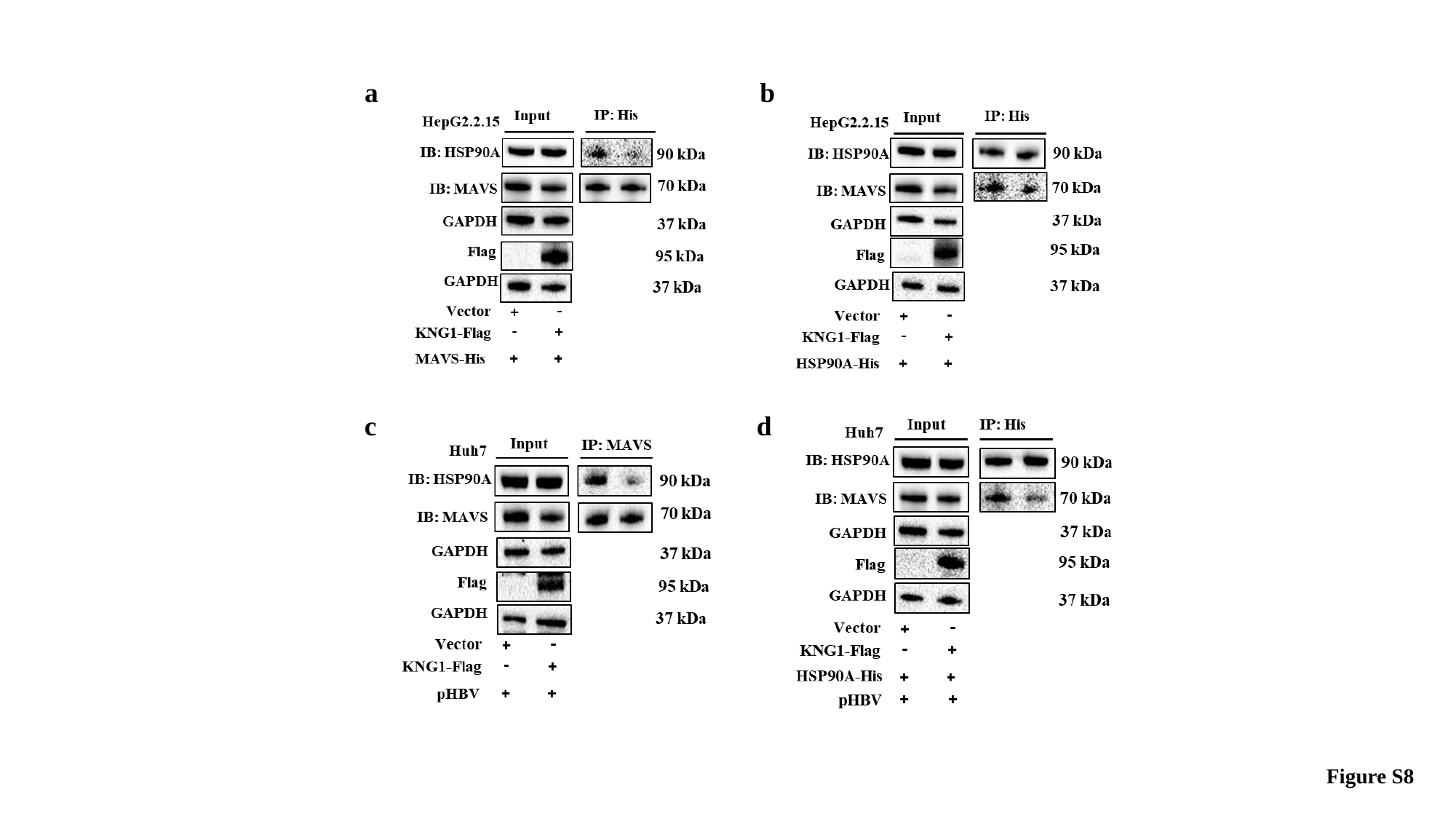

a
b
d
c
Figure S8

## Slide 9
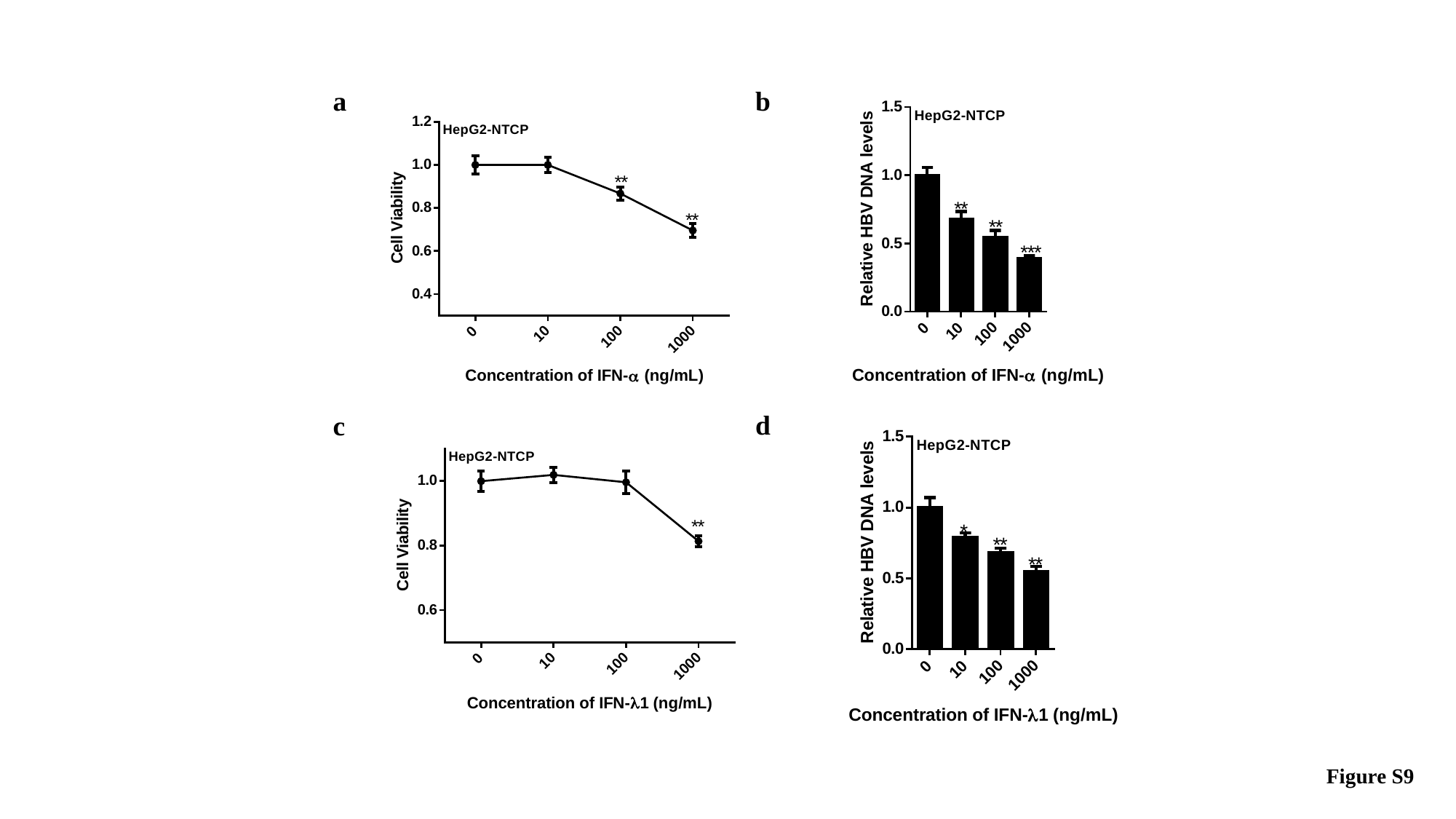

a
b
d
c
Figure S9

## Slide 10
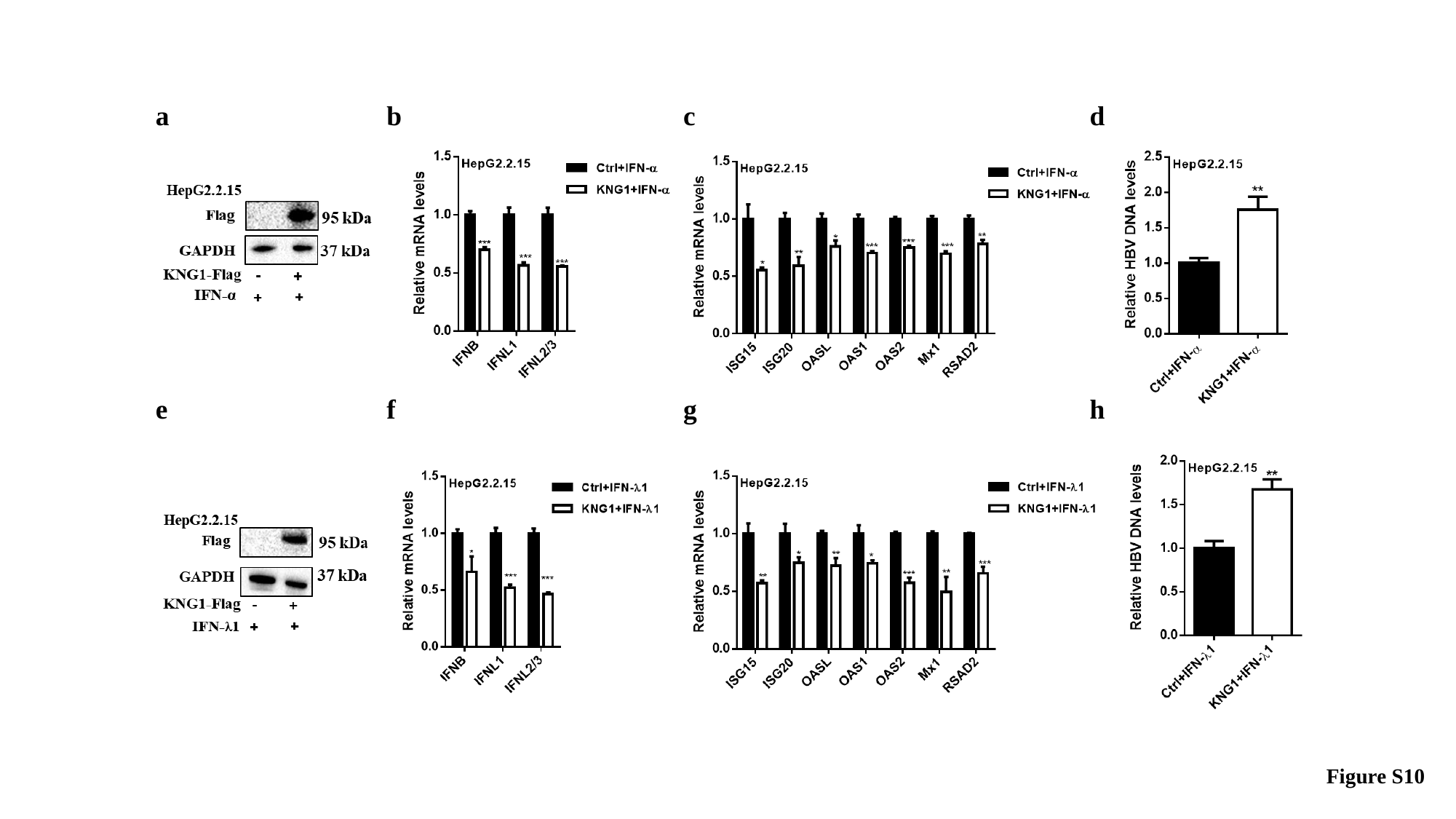

c
d
a
b
g
h
e
f
Figure S10

## Slide 11
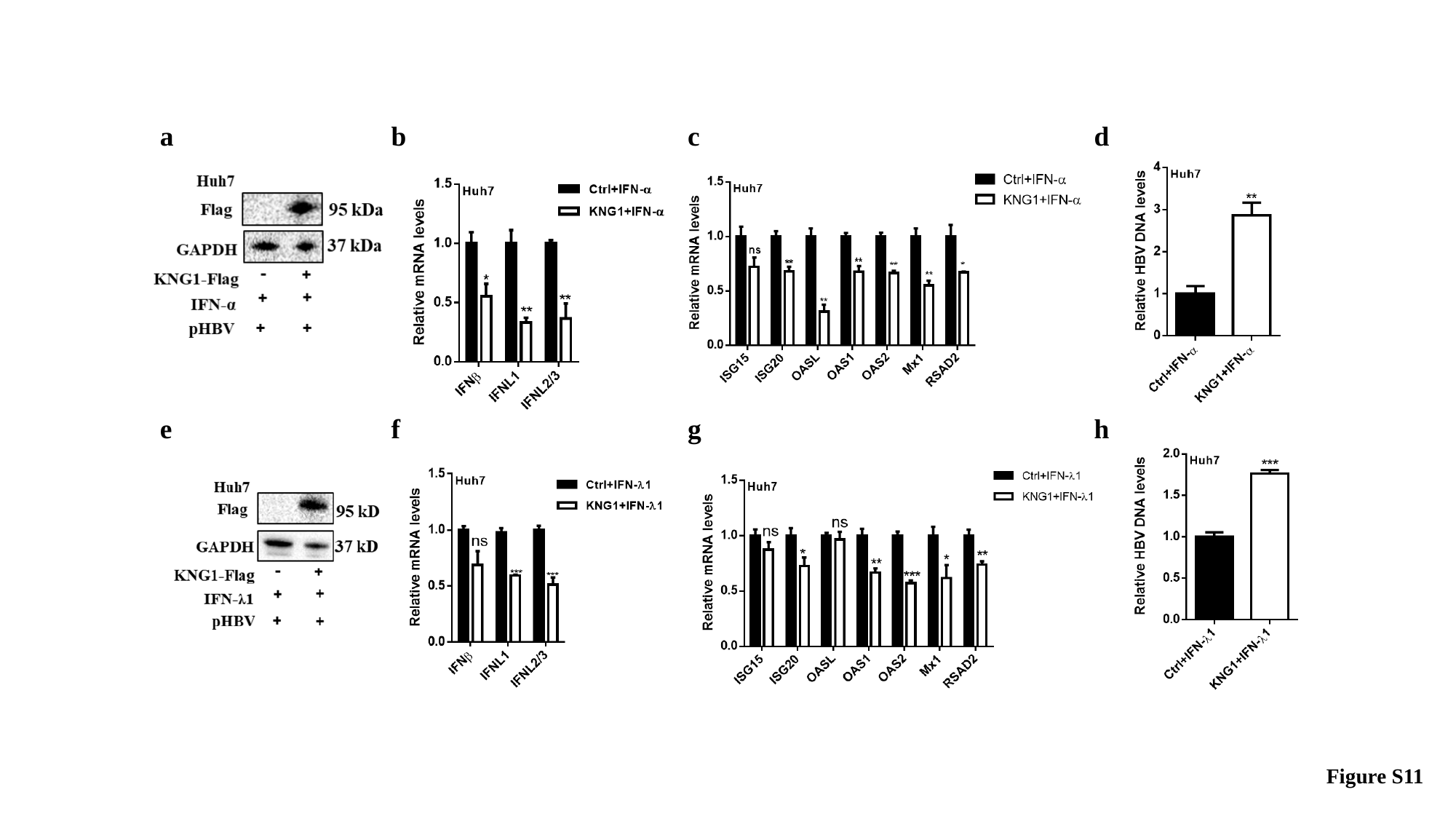

c
d
a
b
g
h
e
f
Figure S11

## Slide 12
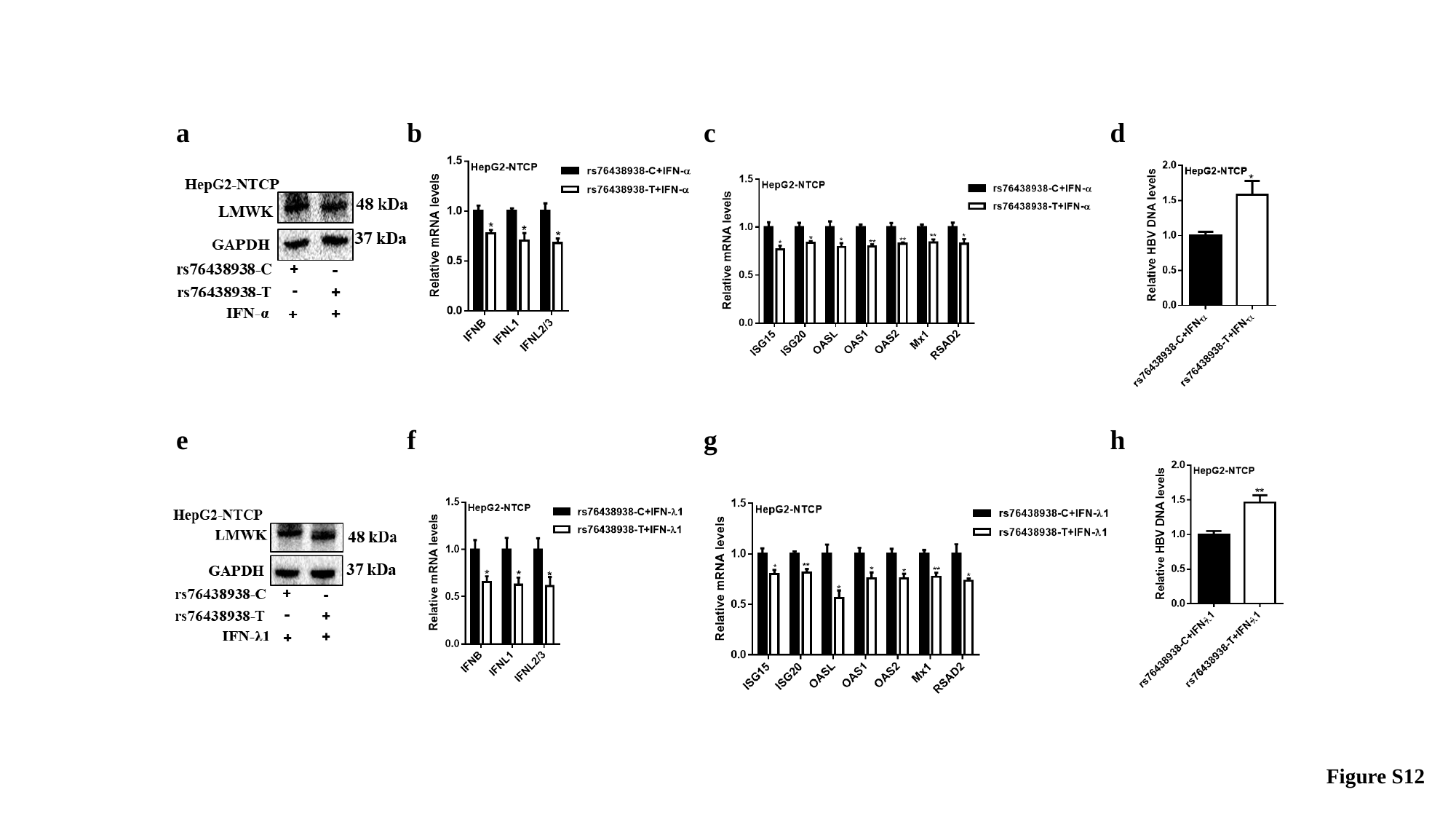

c
d
a
b
g
h
e
f
Figure S12

## Slide 13
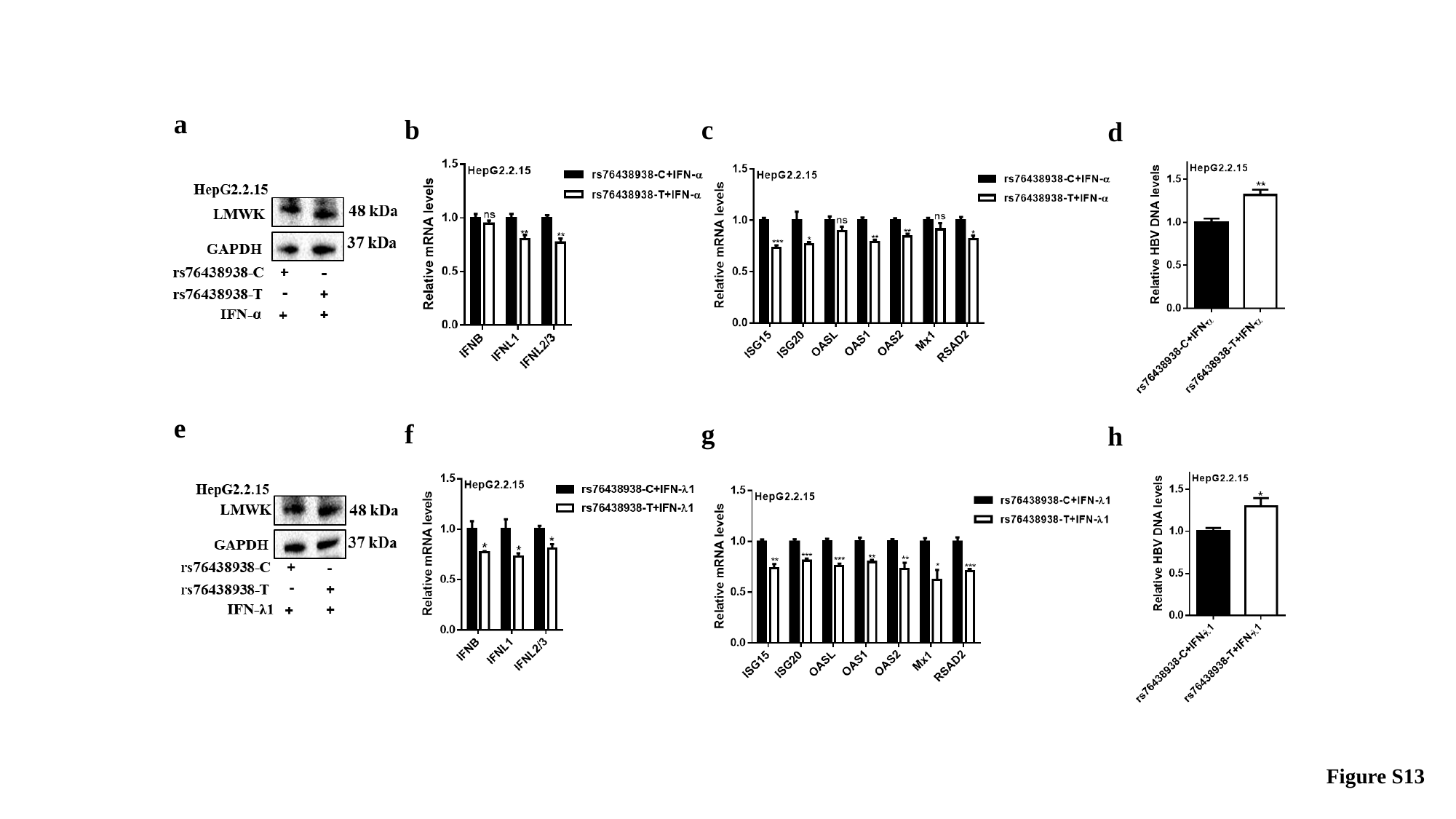

a
b
c
d
e
f
g
h
Figure S13

## Slide 14
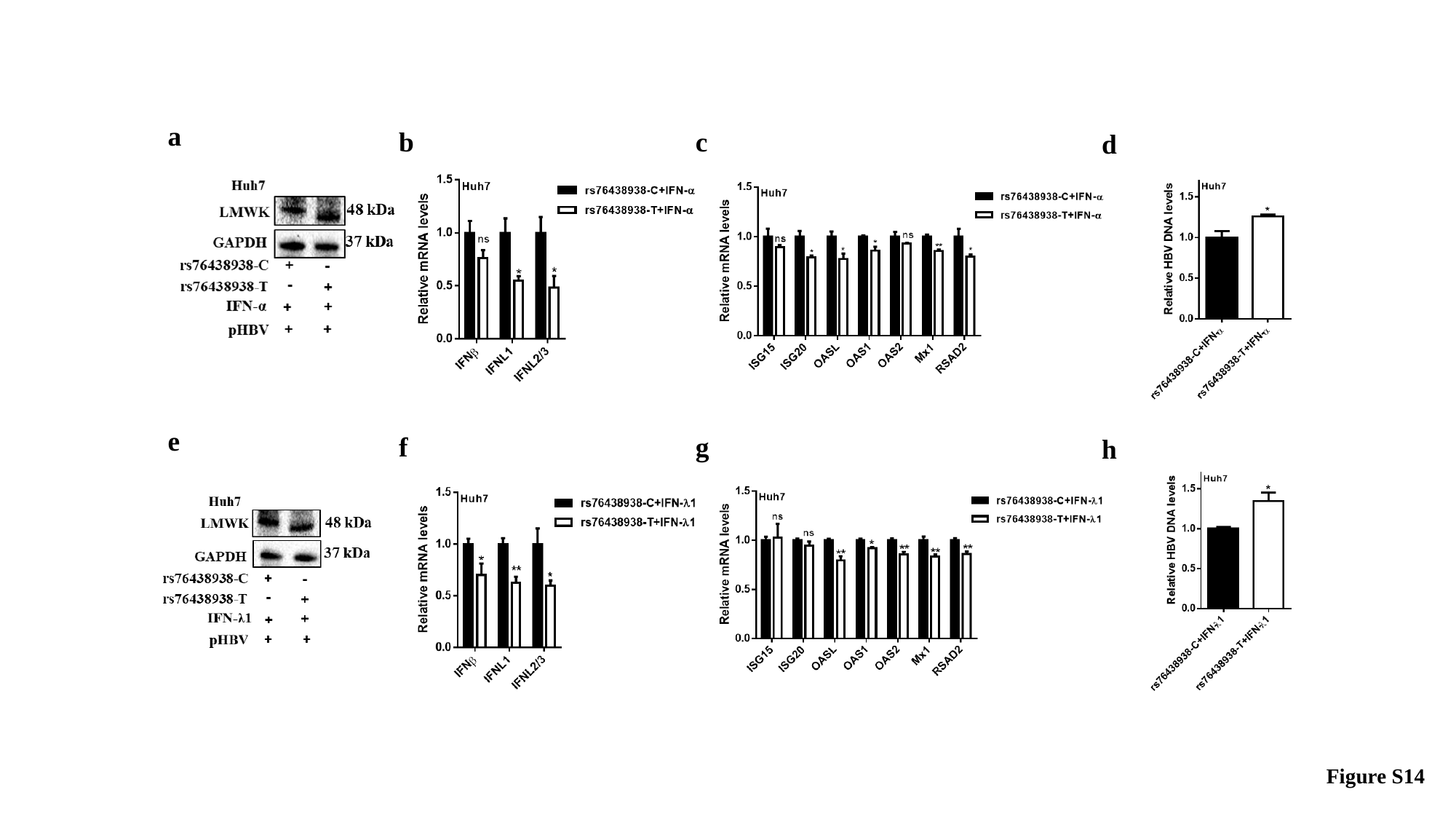

a
b
c
d
e
f
g
h
Figure S14
