# Supplementary material for: An HBV susceptibility variant of KNG1 modulates the therapeutic effects of interferons α and λ1 in HBV infection by promoting MAVS lysosomal degradation
Source: eBioMedicine. 2023 Jul 11;94:104694. doi: 10.1016/j.ebiom.2023.104694 (PMC10435766; doi:10.1016/j.ebiom.2023.104694)

Fig.1c

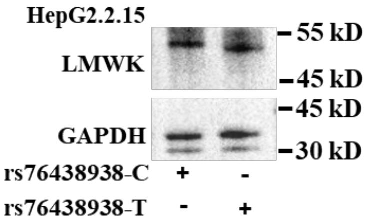

Fig.1g

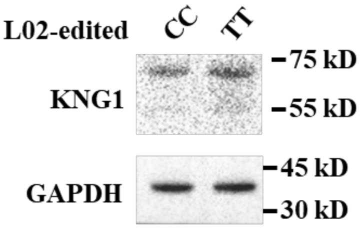

Fig.2c

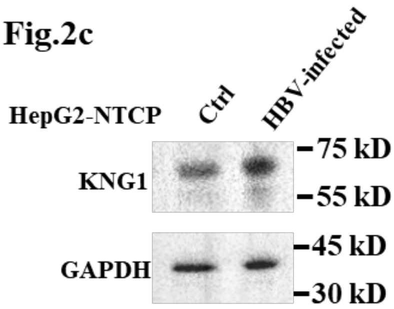

Fig.2d

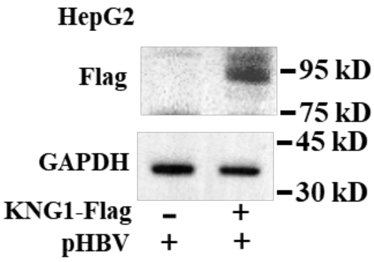

Fig.2f

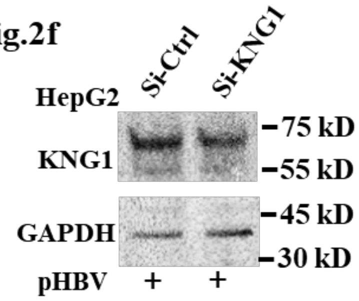

Fig.3b

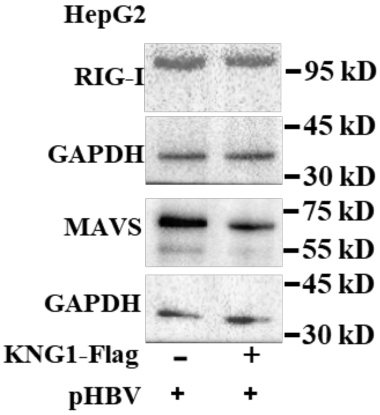

Fig.3e

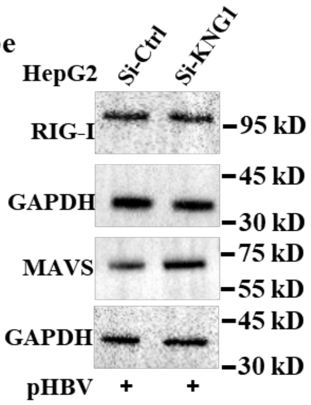

Fig.3g

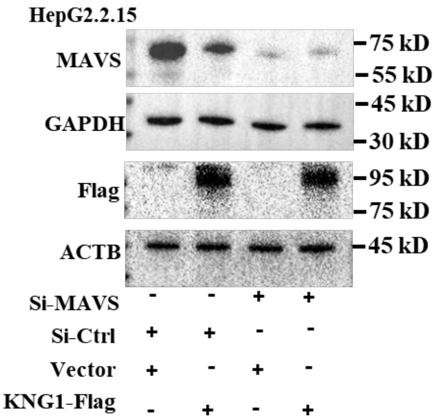

Fig.4a

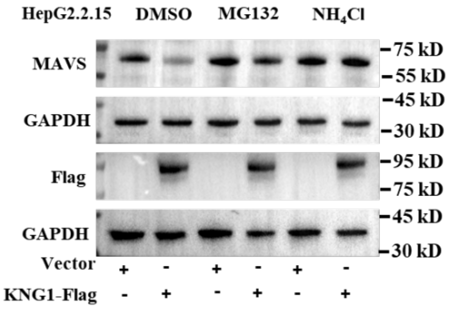

Fig.4b

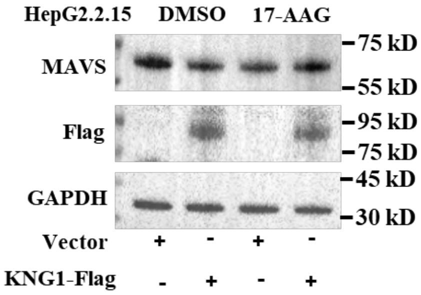

Fig.4d

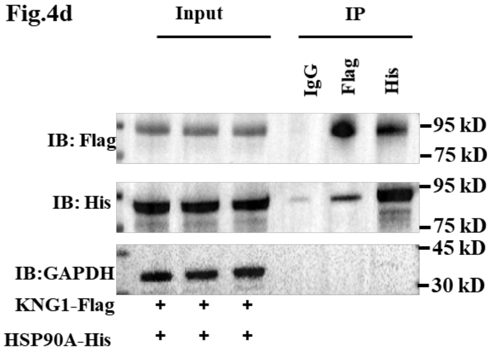

Fig.4e

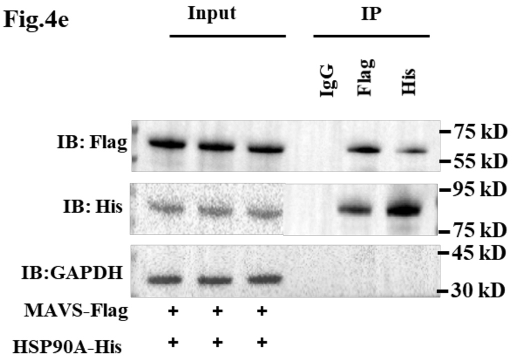

Fig.5b

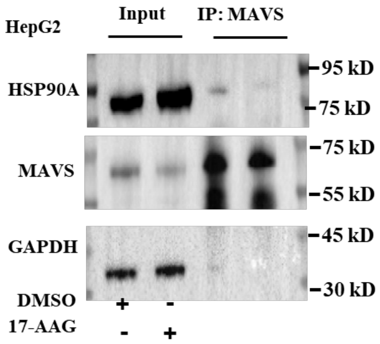

Fig.5c

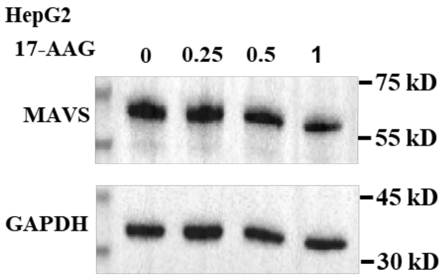

Fig.5d

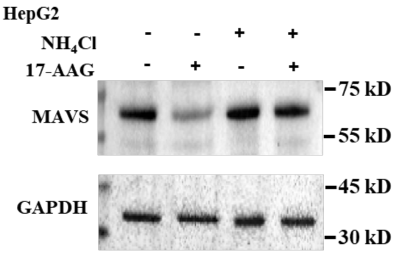

Fig.5e

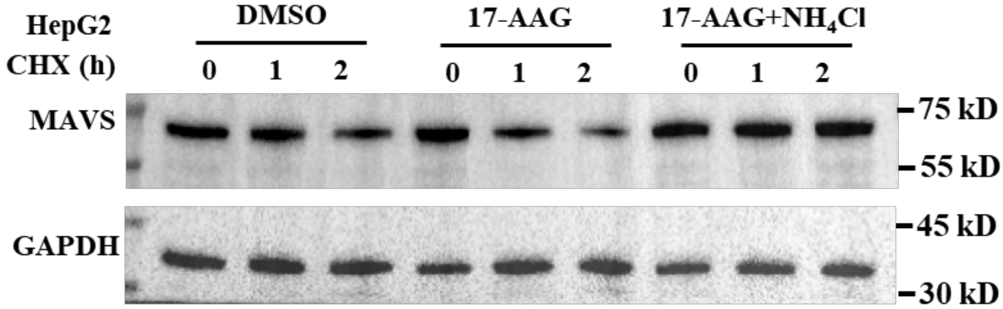

Fig.5f

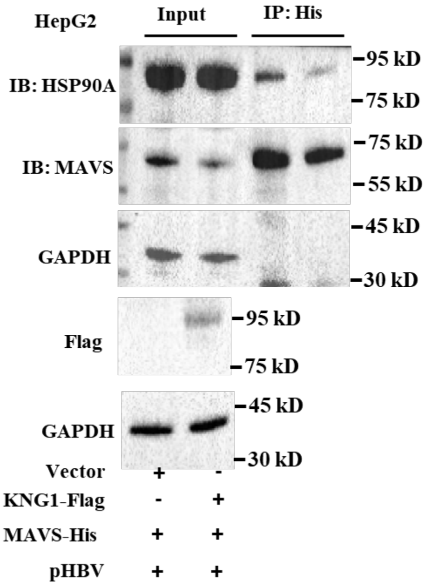

Fig.5g

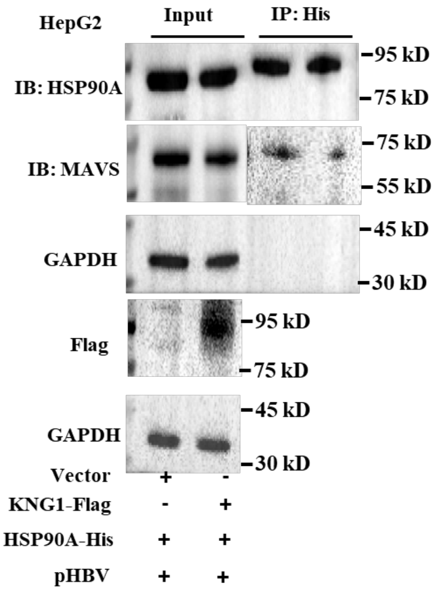

Fig.5h

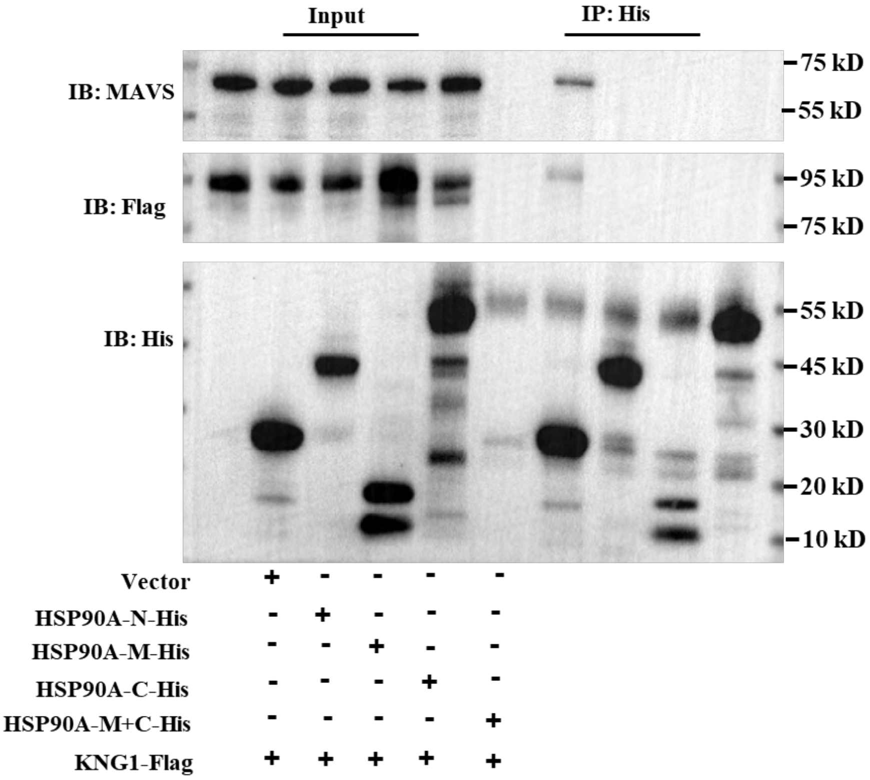

Fig.6d

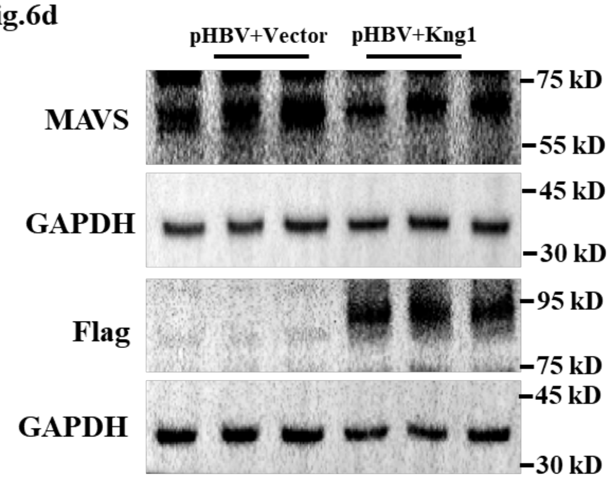

Fig.7a

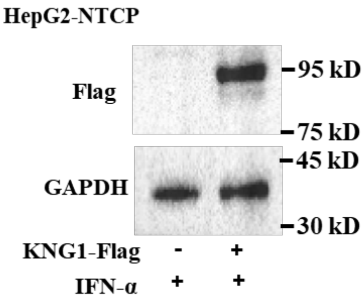

Fig.7e

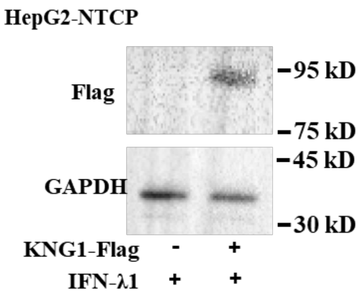

Supplementary Fig.1b

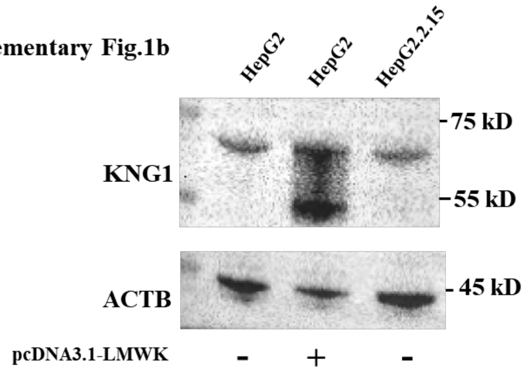

Supplementary Fig.1c

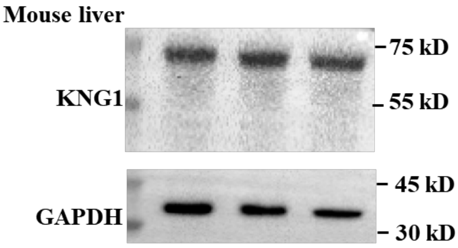

Supplementary Fig.2a

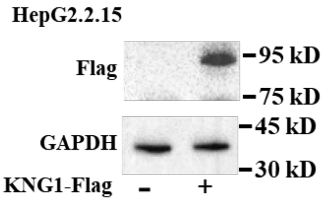

Supplementary Fig.2c

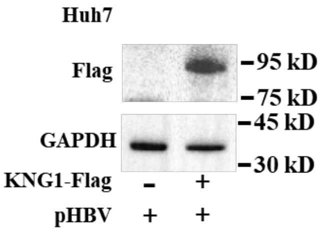

Supplementary Fig.3a

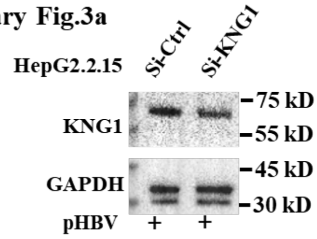

Supplementary Fig.3c

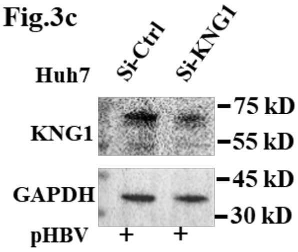

Supplementary Fig.4b

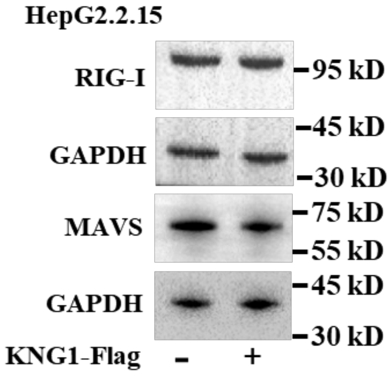

Supplementary Fig.4e

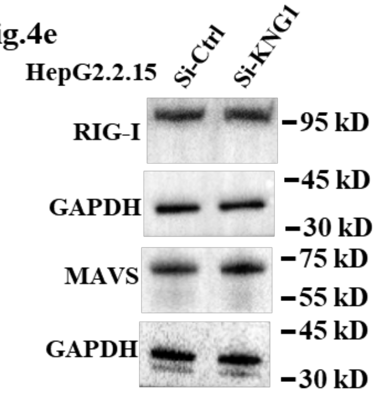

Supplementary Fig.5b

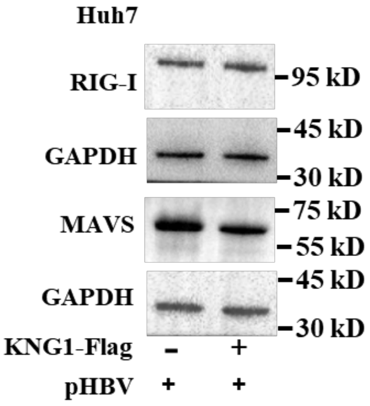

Supplementary Fig.5e

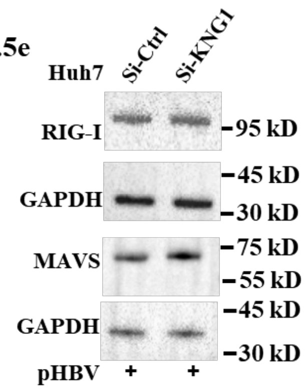

Supplementary Fig.6a

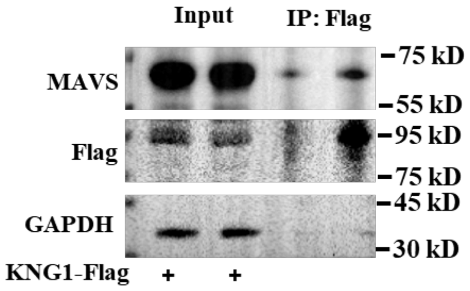

Supplementary Fig.7b

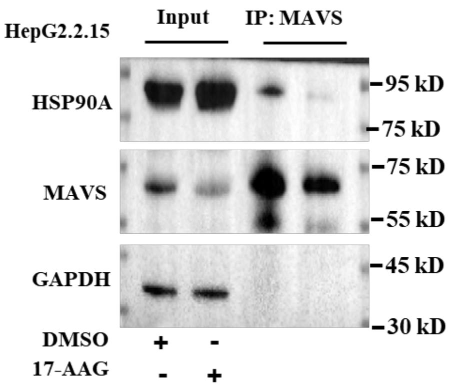

Supplementary Fig.7c

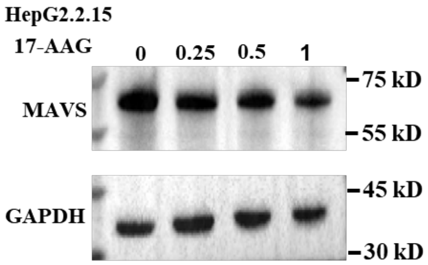

Supplementary Fig.7d

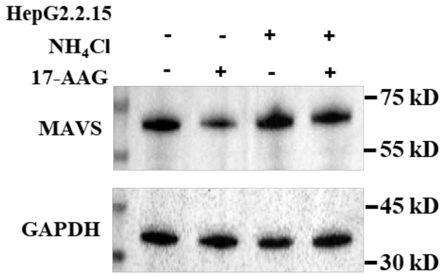

Supplementary Fig.7f

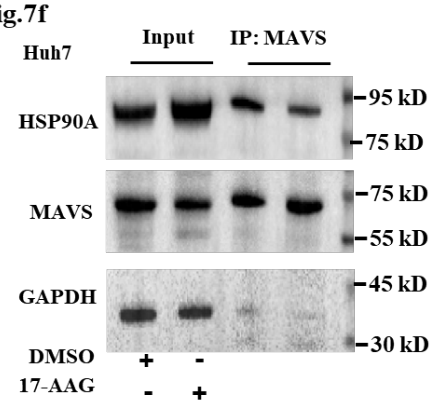

Supplementary Fig.7g

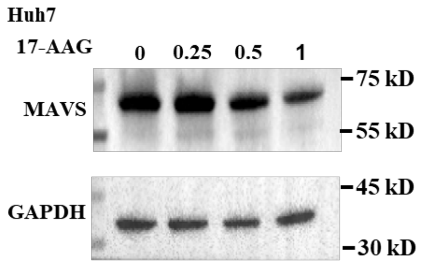

Supplementary Fig.7h

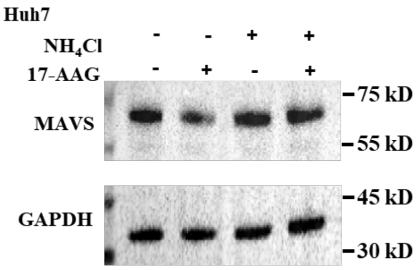

Supplementary Fig.7i

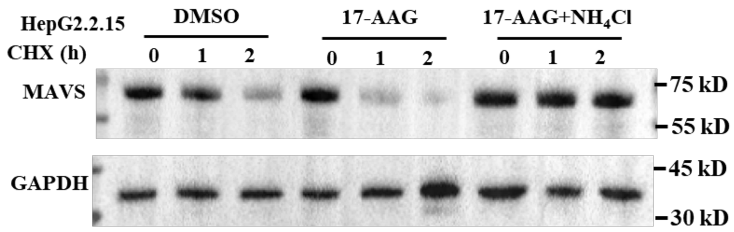

Supplementary Fig.8a

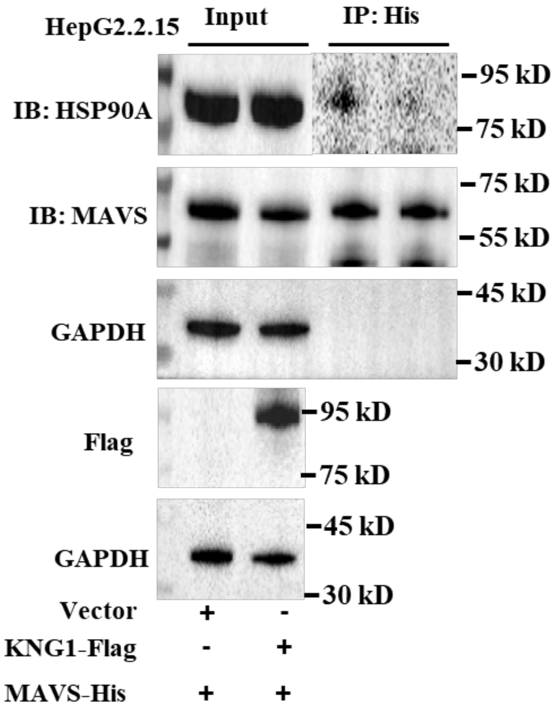

Supplementary Fig.8b

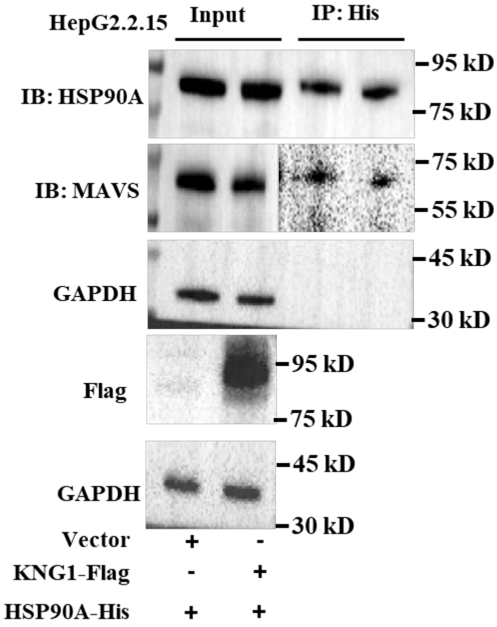

Supplementary Fig.8c

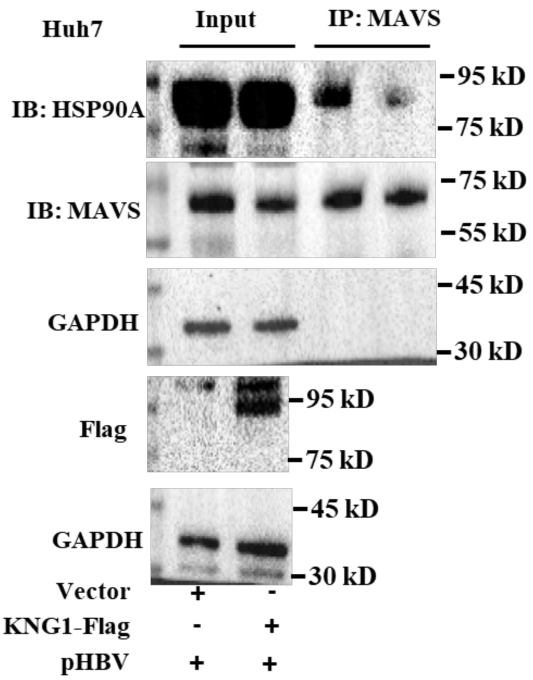

Supplementary Fig.8d

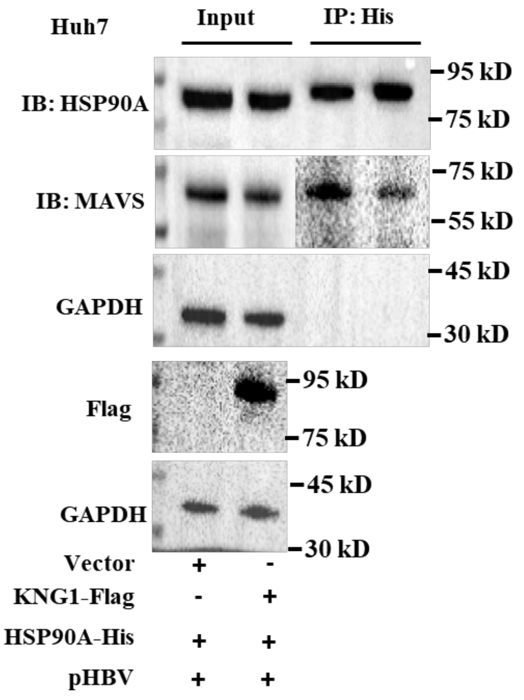

Supplementary Fig.10a

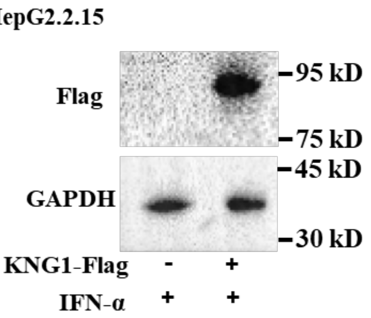

Supplementary Fig.10e

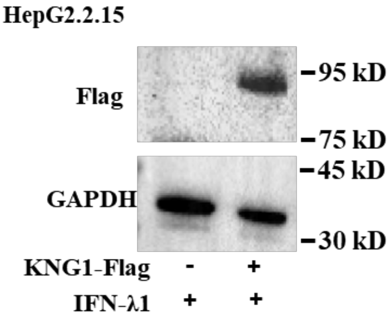

Supplementary Fig.11a

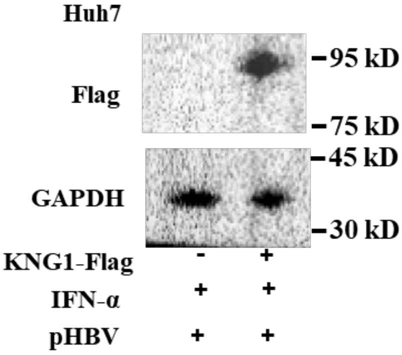

Supplementary Fig.11e

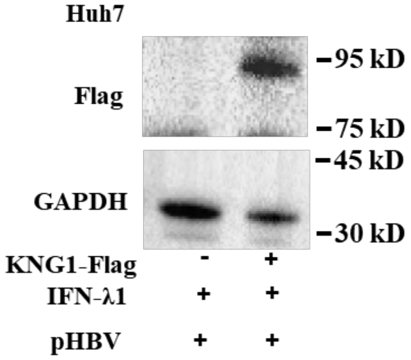

Supplementary Fig.12a

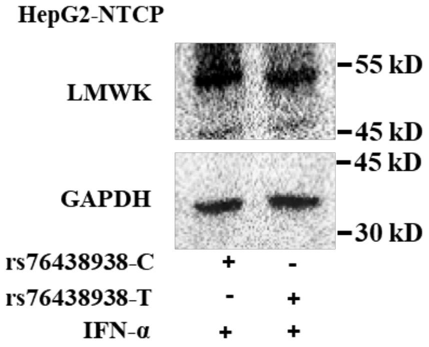

Supplementary Fig.12e

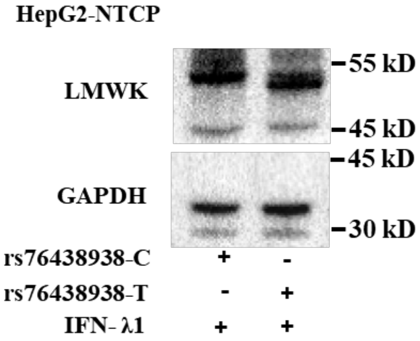

Supplementary Fig.13a

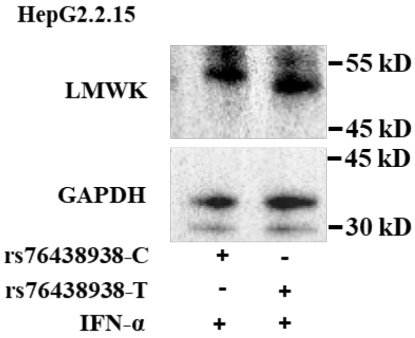

Supplementary Fig.13e

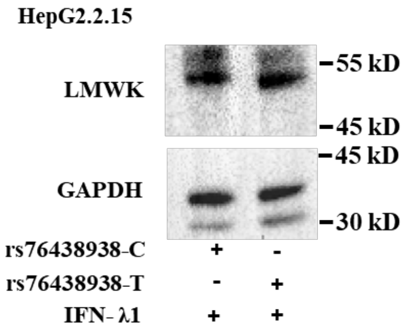

Supplementary Fig.14a

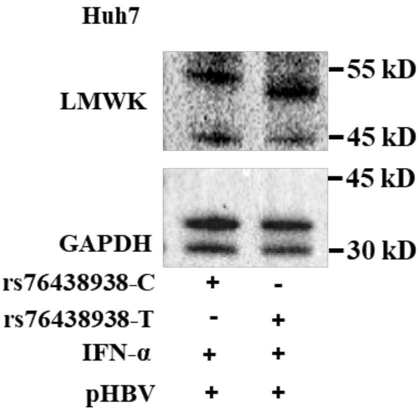

Supplementary Fig.14e

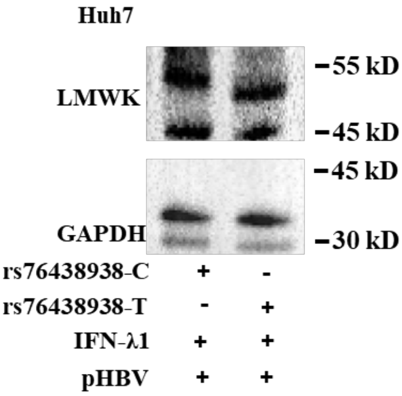

Supplement: Supplemental Western blots [file mmc3.pdf]
