# Supplementary material for: An HBV susceptibility variant of KNG1 modulates the therapeutic effects of interferons α and λ1 in HBV infection by promoting MAVS lysosomal degradation
Source: eBioMedicine. 2023 Jul 11;94:104694. doi: 10.1016/j.ebiom.2023.104694 (PMC10435766; doi:10.1016/j.ebiom.2023.104694)
Supplement: Reagent Validation files [file mmc4.pdf]

For Research Use Only

Kininogen 1 Polyclonal antibody

Catalog Number: 11926-1-AP   5 Publications

proteintech®
Antibodies | ELISA kits | Proteins
www.ptglab.com

Basic Information

Catalog Number:
11926-1-AP

Size:
450 μg/ml

Source:
Rabbit

Isotype:
IgG

Immunogen Catalog Number:
AG2574

GenBank Accession Number:
BC060039

GeneID (NCBI):
3827

Full Name:
kininogen 1

Calculated MW:
644 aa, 72 kDa

Observed MW:
60 kDa, 47 kDa

Purification Method:
Antigen affinity purification

Recommended Dilutions:
WB 1:500-1:1000
IHC 1:50-1:500
IF 1:10-1:100

Applications

Tested Applications:
IF, IHC, WB,ELISA

Cited Applications:
IF, WB

Species Specificity:
human

Cited Species:
human, mouse, rat

Note-IHC: suggested antigen retrieval with TE buffer pH 9.0; (*) Alternatively, antigen retrieval may be performed with citrate buffer pH 6.0

Positive Controls:

WB : HeLa cells, A2780 cells, COLO 320 cells, MCF-7 cells

IHC : human kidney tissue, human endometrial cancer tissue

IF : HepG2 cells,

Background Information

Kininogens are inhibitors of thiol proteases. Kininogen 1 plays important role in Kinin-kallikrein system. This gene is translated into High-molecular weight kininogen (HMWK) and low-molecular weight kininogen (LMWK) after alternative splicing. HMWK is produced by the liver together with prekallikrein. It acts mainly as a cofactor on coagulation and inflammation, and has no intrinsic catalytic activity. LMWK is produced locally by numerous tissues, and secreted together with tissue kallikrein. 11926-1-AP was generated against N-terminal 300 aa of HMW kininogen. It can bind both HMW and LMW kiniogen.

Notable Publications

Author	Pubmed ID	Journal	Application
Bo Ma	30235220	PLoS One	WB
Shen Liu	33144554	Med Sci Monit	WB
Qin Zheng	29734187	Cell Physiol Biochem	WB

Storage

Storage:
Store at -20°C. Stable for one year after shipment.
Storage Buffer:
PBS with 0.02% sodium azide and 50% glycerol pH 7.3.
Aliquoting is unnecessary for -20°C storage

Selected Validation Data

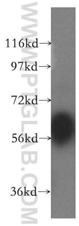

HeLa cells were subjected to SDS PAGE followed by western blot with 11926-1-AP (Kininogen 1 antibody) at dilution of 1:300 incubated at room temperature for 1.5 hours.

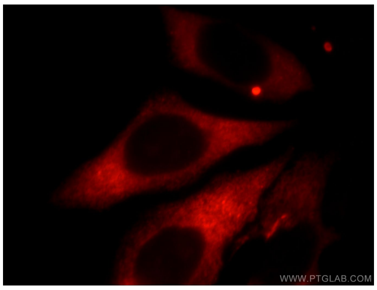

Immunofluorescent analysis of HepG2 cells, using KNG1 antibody 11926-1-AP at 1:25 dilution and Rhodamine-labeled goat anti-rabbit IgG (red).

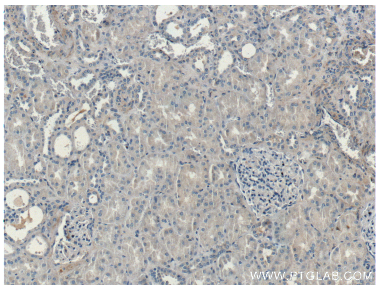

Immunohistochemical analysis of paraffin-embedded human kidney tissue slide using 11926-1-AP (Kininogen 1 antibody at dilution of 1:200 (under 10x lens).

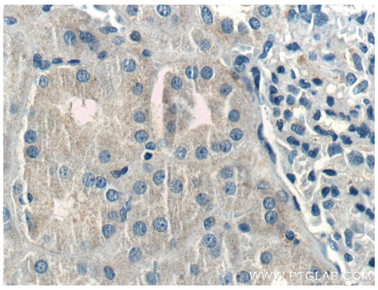

Immunohistochemical analysis of paraffin-embedded human kidney tissue slide using 11926-1-AP (Kininogen 1 antibody at dilution of 1:200 (under 40x lens).

For Research Use Only

MAVS; VISA Polyclonal antibody

Catalog Number: 14341-1-AP Featured Product 50 Publications

proteintech®
Antibodies | ELISA kits | Proteins
www.ptglab.com

Basic Information

Catalog Number:
14341-1-AP

Size:
800 μg/ml

Source:
Rabbit

Isotype:
IgG

Immunogen Catalog Number:
AG5655

GenBank Accession Number:
BC044952

GeneID (NCBI):
57506

Full Name:
mitochondrial antiviral signaling protein

Calculated MW:
57 kDa

Observed MW:
50-55 kDa, 70-75 kDa

Purification Method:
Antigen affinity purification

Recommended Dilutions:
WB 1:2000-1:16000
IP 0.5-4.0 ug for IP and 1:500-1:2000 for WB
IHC 1:250-1:1000
IF 1:50-1:500

Applications

Tested Applications:
IF, IHC, IP, WB, ELISA

Cited Applications:
CoIP, IF, IP, RIP, WB

Species Specificity:
human

Cited Species:
human, monkey, mouse, pig

Note-IHC: suggested antigen retrieval with TE buffer pH 9.0; (*) Alternatively, antigen retrieval may be performed with citrate buffer pH 6.0

Positive Controls:

WB : A431 cells, Jurkat cells, HeLa cells, HuH-7 cells, HepG2 cells

IP : HEK-293 cells,

IHC : human breast cancer tissue, human skin tissue

IF : HeLa cells,

Background Information

Mitochondrial antiviral-signaling protein (MAVS) is also known as virus-induced-signaling adapter (VISA) or IFN-β promoter stimulator protein 1 (IPS-1), it is widely involved and required for innate immune defense against viruses. MAVS, present in T cells, monocytes, epithelial cells and hepatocytes, contains CARD and transmembrane domains which are essential for antiviral functions. MAVS is able to interact with various cellular proteins including DDX58/RIG-I, IFIH1/MDA5, TRAF2, TRAF6, TMEM173/MITA, IFIT3 and etc. It can undergoe phosphorylation on multiple sites and ubiquitination, which may together cause the molecular weight migrate to about 70 kDa despite the predicated 57 kDa.

Notable Publications

Author	Pubmed ID	Journal	Application
Jiangang Zheng	34587973	BMC Vet Res	WB
Ya-Ling Yang	36174668	Eur J Pharmacol	WB
Zhuo Luo	32943610	Signal Transduct Target Ther	WB,CoIP

Storage

Storage:
Store at -20°C. Stable for one year after shipment.
Storage Buffer:
PBS with 0.02% sodium azide and 50% glycerol pH 7.3.
Aliquoting is unnecessary for -20°C storage

For technical support and original validation data for this product please contact:
T: 4006900926 E: Proteintech-CN@ptglab.com W: ptgcn.com

This product is exclusively available under Proteintech Group brand and is not available to purchase from any other manufacturer.

Selected Validation Data

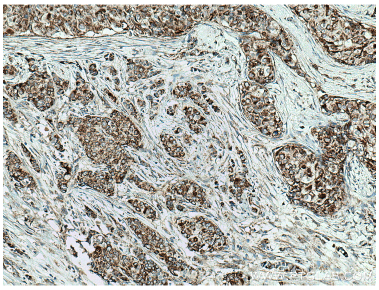

Immunohistochemical analysis of paraffin-embedded human breast cancer tissue slide using 14341-1-AP (MAVS; VISA antibody) at dilution of 1:500 (under 10x lens). Heat mediated antigen retrieval with Tris-EDTA buffer (pH 9.0).

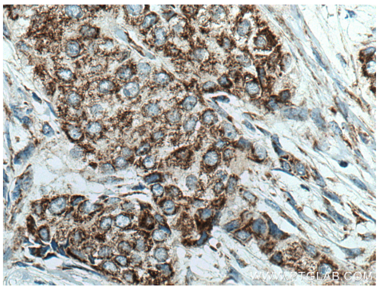

Immunohistochemical analysis of paraffin-embedded human breast cancer tissue slide using 14341-1-AP (MAVS; VISA antibody) at dilution of 1:500 (under 40x lens). Heat mediated antigen retrieval with Tris-EDTA buffer (pH 9.0).

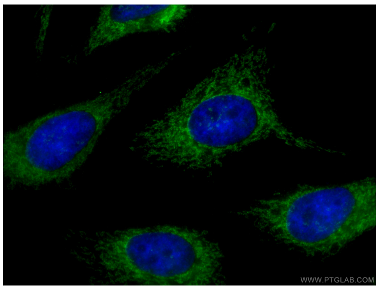

Immunofluorescent analysis of (4% PFA) fixed HeLa cells using 14341-1-AP (MAVS; VISA antibody), at dilution of 1:200 and CoraLite®488-Conjugated AffiniPure Goat Anti-Rabbit IgG(H+L).

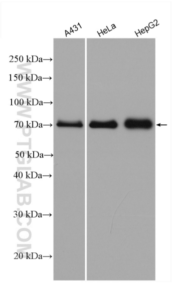

Various lysates were subjected to SDS PAGE followed by western blot with 14341-1-AP (MAVS; VISA antibody) at dilution of 1:8000 incubated at room temperature for 1.5 hours.

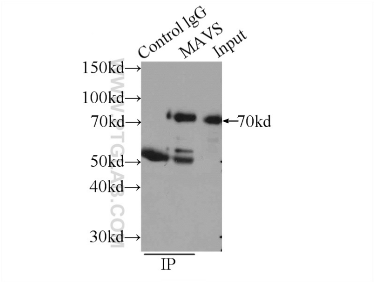

IP Result of anti-MAVS; VISA (IP:14341-1-AP, 3ug; Detection:14341-1-AP 1:1000) with HEK-293 cells lysate 1700ug.

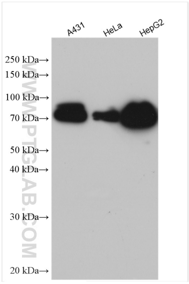

Various lysates were subjected to SDS PAGE followed by western blot with 14341-1-AP (MAVS; VISA antibody) at dilution of 1:10000 incubated at room temperature for 1.5 hours.

For Research Use Only

Beta Actin Polyclonal antibody

Catalog Number:20536-1-AP Featured Product 2581 Publications

proteintech®
Antibodies | ELISA kits | Proteins
www.ptglab.com

Basic Information

Catalog Number:
20536-1-AP

Size:
600 μg/ml

Source:
Rabbit

Isotype:
IgG

Immunogen Catalog Number:
AG14521

GenBank Accession Number:
BC002409

GeneID (NCBI):
60

Full Name:
actin, beta

Calculated MW:
375 aa, 42 kDa

Observed MW:
42 kDa

Purification Method:
Antigen affinity purification

Recommended Dilutions:
WB 1:1000-1:5000
IHC 1:50-1:500
IF 1:200-1:800

Applications

Tested Applications:
FC, IF, IHC, WB, ELISA

Cited Applications:
Cell treatment, CoIP, IF, IHC, IP, WB

Species Specificity:
human, mouse, rat, monkey, canine

Cited Species:
bovine, Camelus bactrianus, canine, carp, chicken, duck, fish, goat, grouper, Hamster

Note-IHC: suggested antigen retrieval with TE buffer pH 9.0; (*) Alternatively, antigen retrieval may be performed with citrate buffer pH 6.0

Positive Controls:

WB : HEK-293 cells, mouse colon tissue, A549 cells, C6 cells, HeLa cells, NIH/3T3 cells, mouse liver tissue, rat liver tissue, rat kidney tissue, Caco-2 cells, RAW 264.7 cells, SMMC-7721 cells, Jurkat cells, rat spleen tissue, HepG2 cells, mouse brain tissue, rat brain tissue

IHC : human colon tissue, human kidney tissue

IF : MDCK cells,

Background Information

Beta Actin, also named as ACTB and F-Actin, belongs to the actin family. Actins are highly conserved globular proteins that are involved in various types of cell motility and are ubiquitously expressed in all eukaryotic cells. At least six isoforms of actins are known in mammals and other vertebrates: alpha (ACTC1, cardiac muscle 1), alpha 1 (ACTA1, skeletal muscle) and 2 (ACTA2, aortic smooth muscle), beta (ACTB), gamma 1 (ACTG1) and 2 (ACTG2, enteric smooth muscle). Beta and gamma 1 are two non-muscle actin proteins. Most actins consist of 376aa, while ACTG2 (rich in muscles) has 375aa and ACTG1(found in non-muscle cells) has only 374aa. Beta actin has been widely used as the internal control in RT-PCR and Western Blotting as a 42-kDa protein. However, the 37-40, 31, 15 kDa cleaved fragment of beta actin can be generated during apoptosis process. This antibody was generated against N-terminal region of human beta actin protein and can cross-react with other actins. (9173887, 11217076, 10229193)

Notable Publications

Author	Pubmed ID	Journal	Application
Xiao-Feng Zhu	36180975	Phytother Res	WB
Yu Xu	36247302	Am J Transl Res	WB
Ling Guo	34867437	Front Physiol	WB

Storage

Storage:
Store at -20°C. Stable for one year after shipment.
Storage Buffer:
PBS with 0.02% sodium azide and 50% glycerol pH 7.3.
Aliquoting is unnecessary for -20°C storage

Selected Validation Data

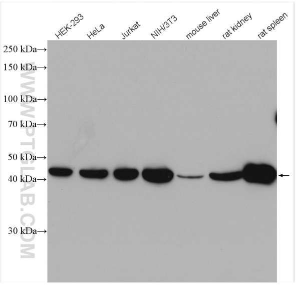

Various lysates were subjected to SDS PAGE followed by western blot with 20536-1-AP (beta Actin antibody) at dilution of 1:5000 incubated at room temperature for 1.5 hours.

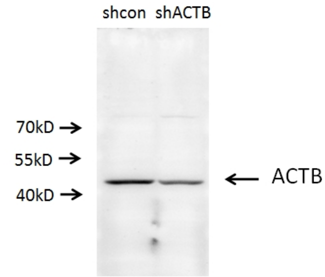

A549 cells (shcontrol and shRNA of Beta actin) were subjected to SDS PAGE followed by western blot with 20536-1-AP (ACTB antibody) at dilution of 1:500.

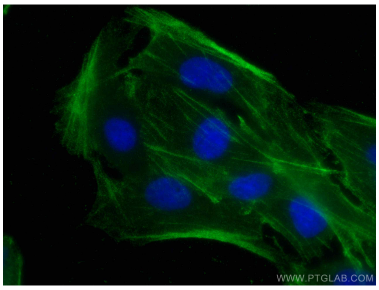

Immunofluorescent analysis of (-20°C Methanol) fixed MDCK cells using Beta Actin antibody (20536-1-AP) at dilution of 1:400 and CoraLite®488-Conjugated AffiniPure Goat Anti-Rabbit IgG(H+L).

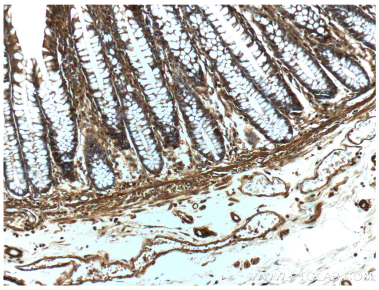

Immunohistochemical analysis of paraffin-embedded human colon tissue slide using 20536-1-AP (beta actin Antibody) at dilution of 1:200 (under 10x lens).

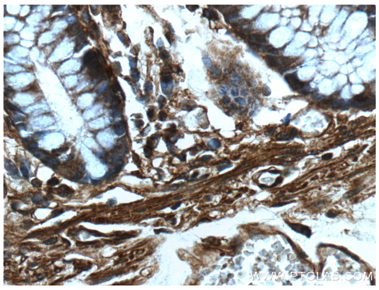

Immunohistochemical analysis of paraffin-embedded human colon tissue slide using 20536-1-AP (beta actin Antibody) at dilution of 1:200 (under 40x lens).

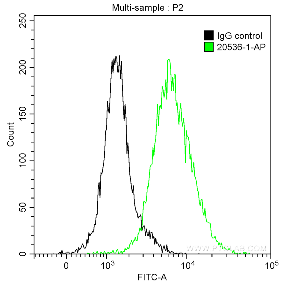

1X10^6 HepG2 cells were intracellularly stained with 0.2 ug Anti-Human Beta Actin (20536-1-AP) and CoraLite®488-Conjugated AffiniPure Goat Anti-Rabbit IgG(H+L) at dilution 1:1000 (green), and 0.2 ug Control Antibody. Cells were fixed with 90% MeOH.

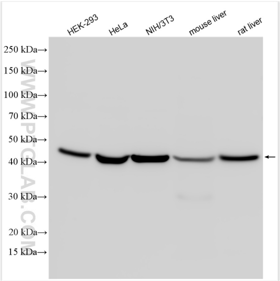

Various lysates were subjected to SDS PAGE followed by western blot with 20536-1-AP (Beta Actin antibody) at dilution of 1:10000 incubated at room temperature for 1.5 hours.

For Research Use Only

DYKDDDDK tag Polyclonal antibody (Binds to FLAG® tag epitope)

proteintech® Antibodies | ELISA kits | Proteins www.ptglab.com

Catalog Number:20543-1-AP 653 Publications

Basic Information

Catalog Number:
20543-1-AP

Size:
600 μg/ml

Source:
Rabbit

Isotype:
IgG

Immunogen Catalog Number:
AG2329

GenBank Accession Number:

GeneID (NCBI):

Full Name:

Purification Method:
Antigen affinity purification

Recommended Dilutions:
WB 1:20000-1:100000

Applications

Tested Applications:
WB, ELISA

Cited Applications:
ChIP, CoIP, FC, IF, IP, RIP, WB

Species Specificity:
recombinant protein

Cited Species:
human, monkey, mouse, rat, yeast

Positive Controls:

WB : Transfected HEK-293T cells,

Background Information

Protein tags are protein or peptide sequences located either on the C- or N- terminal of the target protein, which facilitates one or several of the following characteristics: solubility, detection, purification, localization and expression. The DYKDDDDK(FLAG) peptide has been used extensively as a general tag in expression vectors. This peptide can be expressed and detected with the protein of interest as an amino-terminal or carboxy-terminal fusion. N-terminal DDDDK vectors provide an Ek cleavage site for removal of the fusion tag. The DDDDK peptide is likely to be located on the surface of a fusion protein because of its hydrophilic nature. As a result, the DDDDK peptide is more likely to be accessible to antibodies. A DDDDK-tag can be used in many different assays that require recognition by an antibody, such as western blotting, immunocytochemistry, immunoprecipitation, flow cytometry, protein purification, and in the study of protein-protein interactions, cell ultrastructure, and protein localization and so on. This antibody is a rabbit polyclonal antibody raised against 3xFlag (3xDYKDDDDKT) sequence and recognizes the (1x) and (3x)DYKDDDDK peptide and detects DDDDK-tagged proteins. Anti-FLAG is a registered trademark of Sigma-Aldrich Biotechnology.

Notable Publications

Author	Pubmed ID	Journal	Application
Sirwan Sleman	36179070	Viral Immunol	WB,IP
Huanru Wang	31575039	Int J Mol Sci	
M Zatyka	25274773	Hum Mol Genet	IP

Storage

Storage:
Store at -20°C. Stable for one year after shipment.
Storage Buffer:
PBS with 0.02% sodium azide and 50% glycerol pH 7.3.
Aliquoting is unnecessary for -20°C storage

For technical support and original validation data for this product please contact:
T: 4006900926 E: Proteintech-CN@ptglab.com W: ptgcn.com

This product is exclusively available under Proteintech Group brand and is not available to purchase from any other manufacturer.

Selected Validation Data

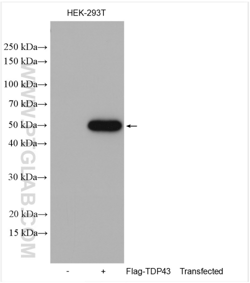

HEK-293T cells and transfected HEK-293T lysates were subjected to SDS PAGE followed by western blot with 20543-1-AP (DYKDDDDK tag antibody) at dilution of 1:50000 incubated at room temperature for 1.5 hours.

For Research Use Only

RIG-1/DDX58 Polyclonal antibody

Catalog Number:20566-1-AP Featured Product 31 Publications

proteintech® Antibodies | ELISA kits | Proteins
www.ptglab.com

Basic Information

Catalog Number:
20566-1-AP

Size:
1000 μg/ml

Source:
Rabbit

Isotype:
IgG

GenBank Accession Number:
NM_014314

GeneID (NCBI):
23586

Full Name:
DEAD (Asp-Glu-Ala-Asp) box polypeptide 58

Calculated MW:
107 kDa

Observed MW:
101/106 kDa

Purification Method:
Antigen affinity purification

Recommended Dilutions:
WB 1:1000-1:6000
IP 0.5-4.0 ug for IP and 1:200-1:1000 for WB
IF 1:10-1:100

Applications

Tested Applications:
IF, IP, WB, ELISA

Cited Applications:
CoIP, IHC, IP, RIP, WB

Species Specificity:
human, mouse

Cited Species:
human, monkey, mouse, pig

Positive Controls:

WB : Jurkat cells, A431 cells, MCF-7 cells, mouse brain tissue, HepG2 cells, MOLT-4 cells

IP : HepG2 cells,

IF : HepG2 cells,

Background Information

DDX58, also named as RIG-1, belongs to the helicase family. It is involved in innate immune defense against viruses. Upon interaction with intracellular dsRNA produced during viral replication, triggers a transduction cascade involving MAVS/IPS1, which results in the activation of NF-kappa-B, IRF3 and IRF7 and the induction of the expression of antiviral cytokines such as IFN-beta and RANTES (CCL5). Detects dsRNA produced from non-self dsDNA by RNA polymerase III, such as Epstein-Barr virus-encoded RNAs (EBERs). It is essential for the production of interferons in response to RNA viruses including paramyxoviruses, influenza viruses, Japanese encephalitis virus and HCV. The antibody is specific to DDX58.

Notable Publications

Author	Pubmed ID	Journal	Application
Liang Zhang	30258002	J Virol	WB
Wei Zhang	25228491	J Gen Virol	WB
Lei-Ke Zhang	27605671	J Virol	WB

Storage

Storage:
Store at -20°C. Stable for one year after shipment.
Storage Buffer:
PBS with 0.02% sodium azide and 50% glycerol pH 7.3.
Aliquoting is unnecessary for -20°C storage

Selected Validation Data

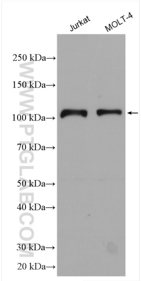

Various lysates were subjected to SDS PAGE followed by western blot with 20566-1-AP (RIG-1/DDX58 antibody) at dilution of 1:3000 incubated at room temperature for 1.5 hours.

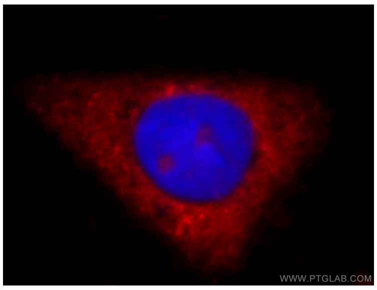

Immunofluorescent analysis of HepG2 cells, using DDX58 antibody 20566-1-AP at 1:25 dilution and Rhodamine-labeled goat anti-rabbit IgG (red). Blue pseudocolor = DAPI (fluorescent DNA dye).

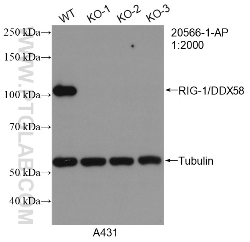

WB result of RIG-1/DDX58 antibody (20566-1-AP; 1:2000; room temperature for 1.5 hours) with wild-type and RIG-1/DDX58 knockout A431 cells.

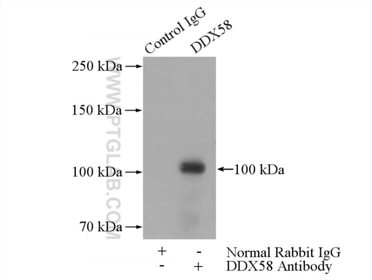

IP Result of anti-DDX58 (IP:20566-1-AP, 4ug; Detection:20566-1-AP 1:300) with HepG2 cells lysate 3600ug.

For Research Use Only

6*His, His-Tag Monoclonal antibody

Catalog Number:66005-1-Ig   625 Publications

proteintech®
Antibodies | ELISA kits | Proteins
www.ptglab.com

Basic Information

Catalog Number:
66005-1-Ig

Size:
1500 μg/ml

Source:
Mouse

Isotype:
IgG1

GenBank Accession Number:

GeneID (NCBI):

Full Name:

Calculated MW:
1 kDa

Purification Method:
Protein A purification

CloneNo.:
1B7G5

Recommended Dilutions:
WB 1:5000-1:50000
IP 0.5-4.0 ug for IP and 1:5000-1:50000 for WB
IF 1:200-1:800

Applications

Tested Applications:
IF, IP, WB, ELISA

Cited Applications:
Cell treatment, ChIP, CoIP, ELISA, FC, IF, IHC, IP, WB

Species Specificity:
recombinant protein

Cited Species:
human, mouse

Positive Controls:

WB : recombinant protein,

IP : Transfected HEK-293 cells,

IF : Transfected HEK-293 cells,

Background Information

Protein tags are protein or peptide sequences located either on the C- or N- terminal of the target protein, which facilitates one or several of the following characteristics: solubility, detection, purification, localization and expression. His-tag is often used for affinity purification and binding assays. Expressed His-tagged proteins can be purified and detected easily because the string of histidine residues binds to several types of immobilized metal ions, including nickel, cobalt and copper, under specific buffer conditions. The His-tag antibody is a useful tool for monitoring of the His-tagged proteins, and recognizes His-tags placed at N-terminal, C-terminal, and internal regions of fusion proteins expressed in bacteria, insect, and mammalian cells.

Notable Publications

Author	Pubmed ID	Journal	Application
Yanan Shao	36177860	Mol Plant Pathol	WB
Yueke Lin	36178239	EMBO Rep	WB,IP
Hao Yang	27708221	Oncotarget	

Storage

Storage:
Store at -20°C. Stable for one year after shipment.
Storage Buffer:
PBS with 0.02% sodium azide and 50% glycerol pH 7.3.
Aliquoting is unnecessary for -20°C storage

For technical support and original validation data for this product please contact:
T: 4006900926   E: Proteintech-CN@ptglab.com   W: ptgcn.com

This product is exclusively available under Proteintech Group brand and is not available to purchase from any other manufacturer.

Selected Validation Data

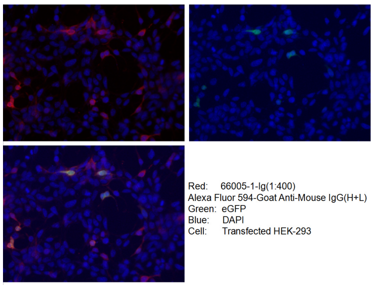

Immunofluorescent analysis of (-20°C Ethanol) fixed Transfected HEK-293 cells using 66005-1-Ig (6*His, His-Tag antibody) at dilution of 1:400 and Alexa Fluor 594-Conjugated AffiniPure Goat Anti-Mouse IgG(H+L).

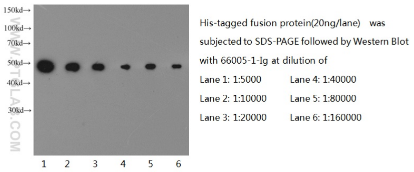

Western blot of 6*His-tagged fusion protein with anti-6*HIS tag (66005-1-Ig) at various dilutions.

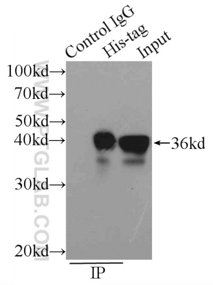

IP Result of anti-6*His, His-Tag (IP:66005-1-Ig, 7ug; Detection:66005-1-Ig 1:10000) with Transfected HEK-293 cells lysate 300ug.

For Research Use Only

DYKDDDDK tag Monoclonal antibody (Binds to FLAG® tag epitope)

proteintech® Antibodies | ELISA kits | Proteins www.ptglab.com

Catalog Number:66008-4-Ig 59 Publications

Basic Information

Catalog Number:
66008-4-Ig

Size:
500 μg/ml

Source:
Mouse

Isotype:
IgG2b

GenBank Accession Number:

GeneID (NCBI):

Full Name:

Purification Method:
Protein A purification

CloneNo.:
8H6A10

Recommended Dilutions:
WB 1:5000-1:50000
IP 0.5-4.0 ug for IP and 1:5000-1:50000 for WB
IF 1:1000-1:4000

Applications

Tested Applications:
IF, IP, WB, ELISA

Cited Applications:
ChIP, CoIP, IF, IP, RIP, WB

Species Specificity:
recombinant protein

Cited Species:
bovine, human, mouse, pig

Positive Controls:

WB : Transfected HEK-293 cells,

IP : Transfected HEK-293 cells,

IF : Transfected HEK-293 cells,

Background Information

DYKDDDDK Tag (Equivalent To FLAG Antibody From Sigma) with the following sequence DYKDDDDK, is a hydrophilic tag for recombinant protein technology. Tags can be used as a tool to localize gene products in a variety of cell types, study proteins topology, and also help to identify and characterize new, low abundance or poorly immunogenic proteins. Due to its high hydrophilic character, the DKYDDDDK tag is likely to be located on the surface of a fusion protein, which enables the tag to be accessible for antibodies. DYKDDDDK Tag Antibody is generated against 1xDYKDDDDK tag (DYKDDDDK) and can recognize protein containing one or more DYKDDDDK tags, independently on N-terminal, C-terminal or internal regions of the target protein. Anti-FLAG is a registered trademark of Sigma-Aldrich Biotechnology.

Notable Publications

Author	Pubmed ID	Journal	Application
Zi-Chao Wang	36163178	Cell Death Dis	WB
Changsheng Jiang	36094311	Microbiol Spectr	CoIP
Guanghui Zhang	36088509	Br J Cancer	WB,IP

Storage

Storage:
Store at -20°C.
Storage Buffer:
PBS with 0.02% sodium azide and 50% glycerol pH 7.3.
Aliquoting is unnecessary for -20°C storage

For technical support and original validation data for this product please contact:
T: 4006900926 E: Proteintech-CN@ptglab.com W: ptgcn.com

This product is exclusively available under Proteintech Group brand and is not available to purchase from any other manufacturer.

Selected Validation Data

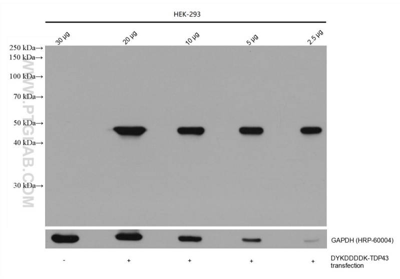

HEK-293 and different loading amount of DYKDDDDK tagged TDP-43 transfected HEK-293 cells were subjected to SDS PAGE followed by western blot with 66008-4-Ig (DYKDDDDK tag antibody) at dilution of 1:20000 incubated at room temperature for 1.5 hours. The membrane was re-blotted with GAPDH antibody (HRP-60004) as loading control.

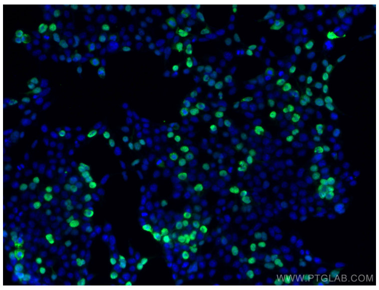

Immunofluorescent analysis of (-20°C Ethanol) fixed DYKDDDDK-TDP43 Transfected HEK-293 cells using DYKDDDDK tag antibody (66008-4-Ig, Clone: 8H6A10) at dilution of 1:2000 and CoraLite®488-Conjugated AffiniPure Goat Anti-Mouse IgG(H+L).

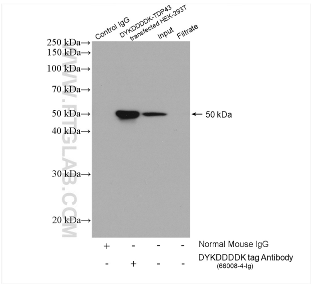

IP result of anti-DYKDDDDK tag(IP:66008-4-Ig, 5 ug; Detection:80010-1-RR 1:10000) with Transfected HEK-293T cells lysate 250 ug.

Leader in Biomolecular Solutions for Life Science

www.abclonal.com

Hsp90α Rabbit mAb

Catalog No.: A5006 **Recombinant** **2 Publications**

Basic Information

Observed MW
90KDa

Calculated MW
85kDa

Category
Primary antibody

Applications
WB,IP

Cross-Reactivity
Human, Mouse, Rat

CloneNo number
ARC1167

Background

The protein encoded by this gene is an inducible molecular chaperone that functions as a homodimer. The encoded protein aids in the proper folding of specific target proteins by use of an ATPase activity that is modulated by co-chaperones. Two transcript variants encoding different isoforms have been found for this gene.

Recommended Dilutions

**WB**	1:500 - 1:2000
**IP**	1:500 - 1:1000

Immunogen Information

Gene ID
3320

Swiss Prot
P07900

Immunogen
A synthetic peptide corresponding to a sequence within amino acids 1-100 of human Hsp90α (P07900).

Synonyms
EL52; HSPN; LAP2; HSP86; HSPC1; HSPCA; Hsp89; Hsp90; LAP-2; HSP89A; HSP90A; HSP90N; Hsp103; HSPCAL1; HSPCAL4; HEL-S-65p

Contact

400-999-6126

cn.market@abclonal.com.cn

www.abclonal.com.cn

Product Information

Source
Rabbit

Isotype
IgG

Purification
Affinity purification

Storage
Store at -20℃. Avoid freeze / thaw cycles.
Buffer: PBS with 0.02% sodium azide,0.05% BSA,50% glycerol,pH7.3.

Validation Data

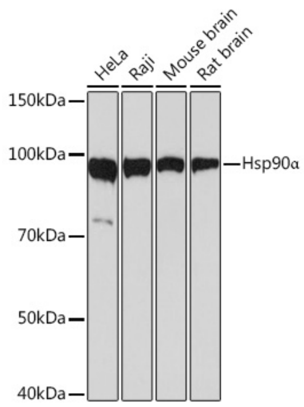

Western blot analysis of extracts of various cell lines, using Hsp90α Rabbit mAb (A5006) at 1:1000 dilution.
Secondary antibody: HRP Goat Anti-Rabbit IgG (H+L) (AS014) at 1:10000 dilution.
Lysates/proteins: 25ug per lane.
Blocking buffer: 3% nonfat dry milk in TBST.
Detection: ECL Basic Kit (RM00020).
Exposure time: 3s.

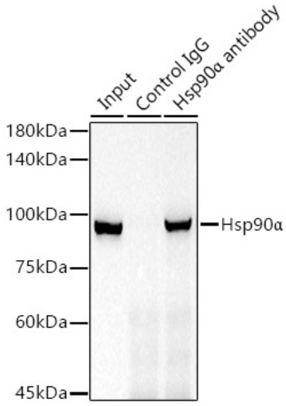

Immunoprecipitation analysis of 300ug extracts of 293T cells using 3ug Hsp90α antibody (A5006). Western blot was performed from the immunoprecipitate using Hsp90α antibody (A5006) at a dilution of 1:1000.

Leader in Biomolecular Solutions for Life Science

www.abclonal.com

GAPDH Mouse mAb (High Dilution)

Catalog No.: AC033 **154 Publications**

Basic Information

Observed MW
36KDa

Calculated MW
36kDa

Category
Loading control antibody

Applications
WB,IHC-P,IF/ICC

Cross-Reactivity
Human, Mouse, Rat

CloneNo number
AMC0062

Background

This gene encodes a member of the glyceraldehyde-3-phosphate dehydrogenase protein family. The encoded protein has been identified as a moonlighting protein based on its ability to perform mechanistically distinct functions. The product of this gene catalyzes an important energy-yielding step in carbohydrate metabolism, the reversible oxidative phosphorylation of glyceraldehyde-3-phosphate in the presence of inorganic phosphate and nicotinamide adenine dinucleotide (NAD). The encoded protein has additionally been identified to have uracil DNA glycosylase activity in the nucleus. Also, this protein contains a peptide that has antimicrobial activity against E. coli, P. aeruginosa, and C. albicans. Studies of a similar protein in mouse have assigned a variety of additional functions including nitrosylation of nuclear proteins, the regulation of mRNA stability, and acting as a transferrin receptor on the cell surface of macrophage. Many pseudogenes similar to this locus are present in the human genome. Alternative splicing results in multiple transcript variants.

Recommended Dilutions

**WB**	1:10000 - 1:760000
**IHC-P**	1:100 - 1:500
**IF/ICC**	1:50 - 1:200

Immunogen Information

Gene ID
2597

Swiss Prot
P04406

Immunogen
Recombinant fusion protein containing a sequence corresponding to amino acids 1-335 of human GAPDH (P04406).

Synonyms
G3PD; GAPD; HEL-S-162eP

Contact

400-999-6126

cn.market@abclonal.com.cn

www.abclonal.com.cn

Product Information

Source
Mouse

Isotype
IgG2b,Kappa

Purification
Affinity purification

Storage
Store at -20℃. Avoid freeze / thaw cycles.
Buffer: PBS with 0.05% proclin300,50% glycerol,pH7.3.

Validation Data

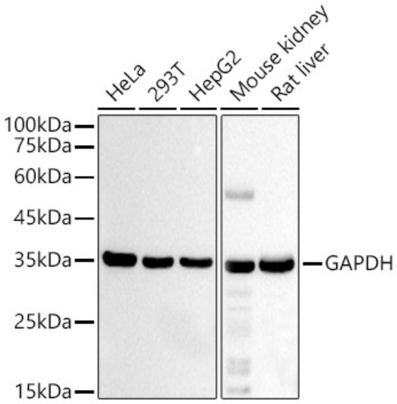

Western blot analysis of various lysates, using GAPDH Mouse mAb(High Dilution) antibody (AC033) at 1:640000 dilution.
Secondary antibody: HRP Goat Anti-Mouse IgG (H+L) (AS003) at 1:10000 dilution.
Lysates/proteins: 25ug per lane.
Blocking buffer: 3% nonfat dry milk in TBST.
Detection: ECL Basic Kit (RM00020).
Exposure time: 30s.

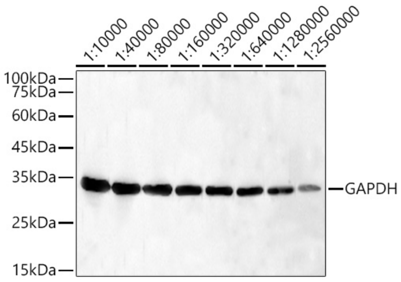

Western blot analysis of over-expressed GAPDH protein, using GAPDH antibody (AC033) at 1:10000-1:2560000 different dilution.
Secondary antibody: HRP Goat Anti-Rabbit IgG (H+L) (AS014) at 1:10000 dilution.
Lysates/proteins: 25ug per lane.
Blocking buffer: 3% nonfat dry milk in TBST.
Detection: ECL Basic Kit (RM00020).
Exposure time: 20s.

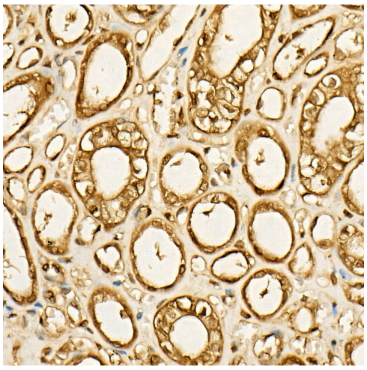

Immunohistochemistry of paraffin-embedded human kidney using GAPDH Mouse mAb (High Dilution) (AC033) at dilution of 1:500 (40x lens).Perform high pressure antigen retrieval with 10 mM citrate buffer pH 6.0 before commencing with IHC staining protocol.

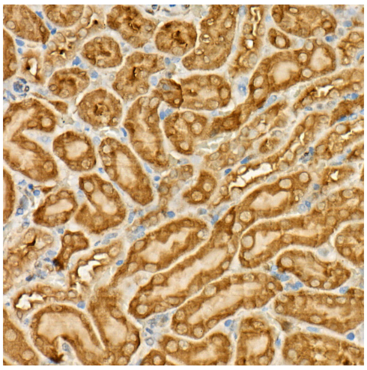

Immunohistochemistry of paraffin-embedded mouse kidney using GAPDH Mouse mAb (High Dilution) (AC033) at dilution of 1:500 (40x lens).Perform high pressure antigen retrieval with 10 mM citrate buffer pH 6.0 before commencing with IHC staining protocol.

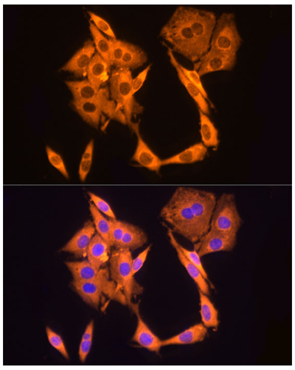

Immunofluorescence analysis of PC-12 cells using GAPDH Mouse mAb (High Dilution) (AC033) at dilution of 1:100 (40x lens). Blue: DAPI for nuclear staining.

Leader in Biomolecular Solutions for Life Science

www.abclonal.com

HRP Goat Anti-Mouse IgG (H+L)

Catalog No.: AS003 **545 Publications**

Basic Information

Observed MW

Calculated MW

Category
Secondary antibody

Applications
ELISA,WB

Cross-Reactivity

Background

Secondary antibodies are affinity-purified antibodies which will work with target-specific primary antibody in the detection, sorting or purification of its specified target. Secondary antibodies offer increased versatility enabling users to use many detection systems (e.g. HRP, AP, fluorescence). They can also provide greater sensitivity through signal amplification as multiple secondary antibodies . Most commonly, secondary antibodies are generated by immunizing the host animal (different from host species of primary antibody) with a pooled population of normal immunoglobulins from the host species of primary antibody and can be further purified and modified (i.e. antibody fragmentation, label conjugation, etc.) to ensure well-characterized specificity to corresponding normal immunoglobulins.

Recommended Dilutions

**ELISA**	1:5000 - 1:10000
**WB**	1:2000 - 1:10000

Immunogen Information

Gene ID

Swiss Prot

Immunogen
Mouse IgG

Synonyms

Contact

400-999-6126

cn.market@abclonal.com.cn

www.abclonal.com.cn

Product Information

Source
Goat

Isotype
Horseradish peroxidase conjugated IgG

Purification
Affinity purification

Storage
Store at -20℃. Avoid freeze / thaw cycles.
Buffer: PBS with 0.75% BSA,50% glycerol,pH7.3.

Validation Data

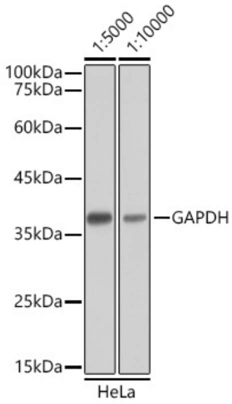

Western blot analysis of extracts of HeLa cells, using GAPDH antibody as the primary antibody.
Secondary antibody: HRP Goat Anti-Mouse IgG (H+L) antibody (AS003) at 1:5000/1:10000 dilution.
Lysates/proteins: 25ug per lane.
Blocking buffer: 3% nonfat dry milk in TBST.
Detection: ECL Basic Kit (RM00020).
Exposure time: 60s.

abcam

Product datasheet

Goat Anti-Rabbit IgG H&L (Alexa Fluor® 488) ab150077

★★★★★ 20 Abreviews 2381 References 16 Images

Overview

**Product name**	Goat Anti-Rabbit IgG H&L (Alexa Fluor® 488)
**Host species**	Goat
**Target species**	Rabbit
**Specificity**	This antibody is specific to Rabbit IgG.
**Tested applications**	**Suitable for:** ICC/IF, Flow Cyt, IHC-P, ELISA, IHC-Fr
**Immunogen**	The details of the immunogen for this antibody are not available.
**Conjugation**	Alexa Fluor® 488. Ex: 495nm, Em: 519nm

Properties

**Form**	Liquid
**Storage instructions**	Shipped at 4°C. Store at +4°C short term (1-2 weeks). Upon delivery aliquot. Store at -20°C. Avoid freeze / thaw cycle. Stable for 12 months at -20°C. Store In the Dark.
**Storage buffer**	Preservative: 0.02% Sodium azide<br>Constituents: 23% Glycerol (glycerin, glycerine), PBS, 1% BSA
**Purity**	Immunogen affinity purified
**Purification notes**	This antibody was isolated by affinity chromatography using antigen coupled to agarose beads.
**Clonality**	Polyclonal
**Isotype**	IgG
**General notes**	**Fluorochrome chart – a complete guide:**

A quick and easy guide to help you select the most appropriate fluorochromes for your next experiment.

Please see **here**.

Alexa Fluor® is a registered trademark of Molecular Probes, Inc, a Thermo Fisher Scientific Company. The Alexa Fluor® dye included in this product is provided under an intellectual property license from Life Technologies Corporation. As this product contains the Alexa Fluor® dye, the purchase of this product conveys to the buyer the non-transferable right to use the purchased product and components of the product only in research conducted by the buyer (whether the buyer is an academic or for-profit entity). As this product contains the Alexa Fluor® dye the sale of this product is expressly conditioned on the buyer not using the product or its components, or any

materials made using the product or its components, in any activity to generate revenue, which may include, but is not limited to use of the product or its components: in manufacturing; (ii) to provide a service, information, or data in return for payment (iii) for therapeutic, diagnostic or prophylactic purposes; or (iv) for resale, regardless of whether they are sold for use in research. For information on purchasing a license to this product for purposes other than research, contact Life Technologies Corporation, 5781 Van Allen Way, Carlsbad, CA 92008 USA or **outlicensing@thermofisher.com**.

Applications

The Abpromise guarantee Our **Abpromise guarantee** covers the use of ab150077 in the following tested applications.

The application notes include recommended starting dilutions; optimal dilutions/concentrations should be determined by the end user.

Application	Abreviews	Notes
ICC/IF	★★★★★ (7)	1/200 - 1/1000.
Flow Cyt		1/2000 - 1/4000.
IHC-P	★★★★★ (3)	Use at an assay dependent concentration.
ELISA		Use at an assay dependent concentration.
IHC-Fr	★★★★★ (5)	Use at an assay dependent concentration.

Images

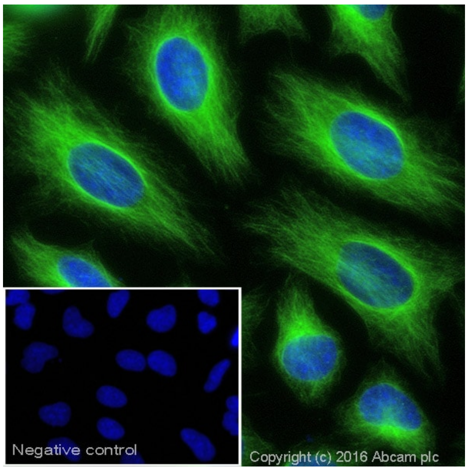

Immunocytochemistry/ Immunofluorescence - Goat Anti-Rabbit IgG H&L (Alexa Fluor® 488) (ab150077)

ICC/IF image of beta Tubulin staining in HeLa cells. The cells were 100% methanol fixed (5 min), permeabilized with 0.1% Triton X-100 for 5 minutes and then incubated in 1% BSA / 10% normal goat serum / 0.3M glycine in 0.1% PBS-Tween for 1h to block non-specific protein-protein interactions. The cells were then incubated with the primary antibody (**ab6046**, 5µg/ml) overnight at +4°C. The secondary antibody (green) was ab150077 Alexa Fluor® 488 goat anti-rabbit IgG (H+L) used at 2µg/ml for 1h. DAPI was used to stain the cell nuclei (blue) at a concentration of 1.43µM.

The negative control (inset) is a secondary-only assay to demonstrate low non-specific binding of the secondary antibody.

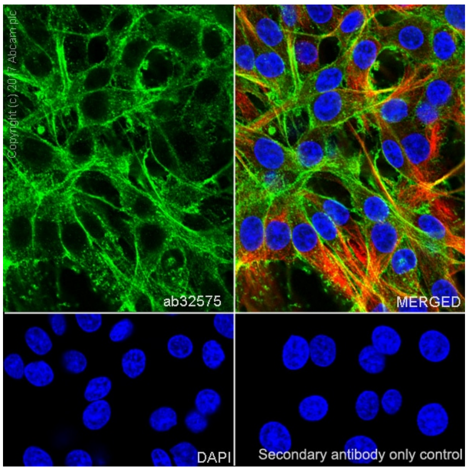

Immunocytochemistry/ Immunofluorescence - Goat Anti-Rabbit IgG H&L (Alexa Fluor® 488) (ab150077)

Immunocytochemistry/ Immunofluorescence analysis of C6(Rat glial tumor glial cell) cells labeling alpha smooth muscle Actin with purified **ab32575** at 1/100 dilution (0.71 µg/ml). Cells were fixed in 4% paraformaldehyde and permeabilized with 0.1% Triton X-100. Cells were counterstained with Ab195889 Anti-alpha Tubulin antibody [DM1A] - Microtubule Marker (Alexa Fluor® 594) 1/200 (2.5 µg/ml). Goat anti rabbit IgG (Alexa Fluor® 488, ab150077) was used as the secondary antibody at 1/1000 dilution (2 µg/ml) dilution. DAPI was used as nuclear counterstain. PBS instead of the primary antibody was used as the secondary antibody only control.

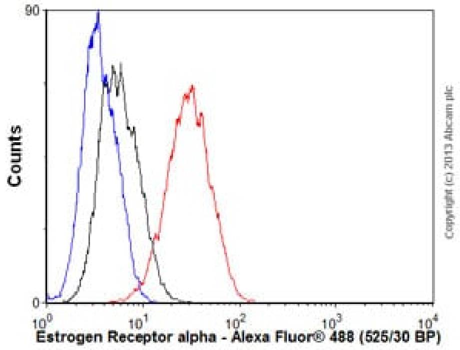

Flow Cytometry (Intracellular) - Goat Anti-Rabbit IgG H&L (Alexa Fluor® 488) (ab150077)

Overlay histogram showing MCF7 cells stained with unpurified **ab32063** (red line). The cells were fixed with 4% paraformaldehyde (10 min) and then permeabilized with 0.1% PBS-Tween for 20 min. The cells were then incubated in 1x PBS / 10% normal goat serum / 0.3M glycine to block non-specific protein-protein interactions followed by the antibody (**ab32063**, 1/1000 dilution) for 30 min at 22°C. The secondary antibody used was Alexa Fluor® 488 goat anti-rabbit IgG (H+L) (ab150077) at 1/2000 dilution for 30 min at 22°C. Isotype control antibody (black line) was rabbit IgG (monoclonal) (1µg/1x106 cells) used under the same conditions. Unlabelled sample (blue line) was also used as a control. Acquisition of >5,000 events were collected using a 20mW Argon ion laser (488nm) and 525/30 bandpass filter. This antibody gave a positive signal in MCF7 cells fixed with 80% methanol (5 min)/permeabilized with 0.1% PBS-Tween for 20 min used under the same conditions.

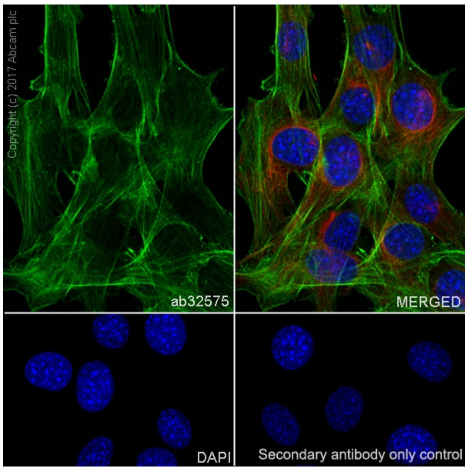

Immunocytochemistry/ Immunofluorescence - Goat Anti-Rabbit IgG H&L (Alexa Fluor® 488) (ab150077)

Immunocytochemistry/ Immunofluorescence analysis of NIH/3T3(Mouse embryonic fibroblast) cells labeling alpha smooth muscle Actin with purified **ab32575** at 1/500 dilution (5.2 µg/ml). Cells were fixed in 4% paraformaldehyde and permeabilized with 0.1% Triton X-100. Cells were counterstained with Ab195889 Anti-alpha Tubulin antibody [DM1A] - Microtubule Marker (Alexa Fluor® 594) 1/200 (2.5 µg/ml). Goat anti rabbit IgG (Alexa Fluor® 488, ab150077) was used as the secondary antibody at 1/1000 dilution (2 µg/ml) dilution. DAPI was used as nuclear counterstain. PBS instead of the primary antibody was used as the secondary antibody only control.

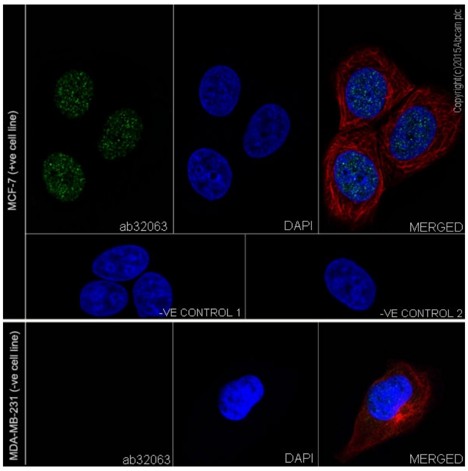

Immunocytochemistry/ Immunofluorescence - Goat Anti-Rabbit IgG H&L (Alexa Fluor® 488) (ab150077)

Immunocytochemistry/Immunofluorescence analysis of MCF-7 cells labelling Estrogen Receptor alpha with purified **ab32063** at 1/1000. Cells were fixed with 4% paraformaldehyde and permeabilized with 0.1% Triton X-100. ab150077, an Alexa Fluor® 488-conjugated goat anti-rabbit IgG (1/1000) was used as the secondary antibody. The cells were co-stained with **ab7291**, a mouse anti-tubulin (1/1000) using **ab150120**, an Alexa Fluor® 594-conjugated goat anti-mouse IgG (1/1000) as the secondary antibody. Nuclei counterstained with DAPI (blue).

Control 1: primary antibody (1/1000) and secondary antibody, **ab150120**, an Alexa Fluor® 594-conjugated goat anti-mouse IgG (1/1000).

Control 2: **ab7291** (1/1000) and secondary antibody, ab150077, an Alexa Fluor® 488-conjugated goat anti-rabbit IgG (1/1000).

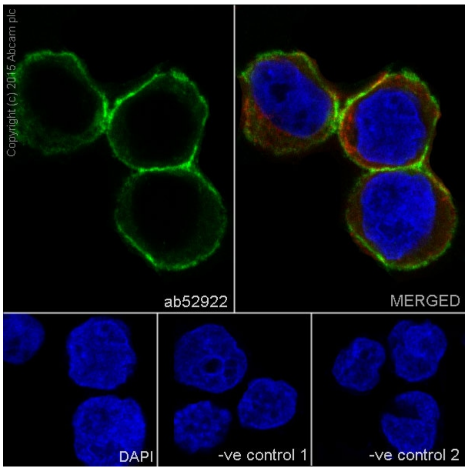

Immunocytochemistry/ Immunofluorescence - Goat Anti-Rabbit IgG H&L (Alexa Fluor® 488) (ab150077)

Immunocytochemistry/Immunofluorescence analysis of Raji (human Burkitt's lymphoma) cells labelling HLA A with purified **ab52922** at 1/100. Cells were fixed with 4% paraformaldehyde and permeabilized with 0.1% Triton X-100. ab150077, an Alexa Fluor® 488-conjugated goat anti-rabbit IgG (1/1000) was used as the secondary antibody. DAPI (blue) was used as the nuclear counterstain. **ab7291**, a mouse anti-tubulin (1/1000) and **ab150120**, an Alexa Fluor® 594-conjugated goat anti-mouse IgG (1/1000) were also used.

Control 1: primary antibody (1/100) and secondary antibody, **ab150120**, an Alexa Fluor® 594-conjugated goat anti-mouse IgG (1/500).

Control 2: **ab7291** (1/1000) and secondary antibody, ab150077, an Alexa Fluor® 488-conjugated goat anti-rabbit IgG (1/500).

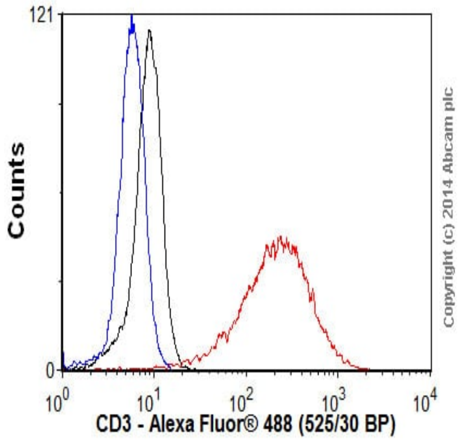

Flow Cytometry - Goat Anti-Rabbit IgG H&L (Alexa Fluor® 488) (ab150077)

Overlay histogram showing Jurkat cells stained with **ab16669** (red line). The cells were fixed with 4% paraformaldehyde (10 min) and then permeabilized with 0.1% PBS-Tween for 20 min. The cells were then incubated in 1x PBS / 10% normal goat serum / 0.3M glycine to block non-specific protein-protein interactions followed by the antibody (**ab16669**, 1/1000 dilution) for 30 min at 22°C. The secondary antibody Goat anti-rabbit IgG H&L (Alexa Fluor® 488) (ab150077) was used at 1/4000 dilution for 30 min at 22°C. Isotype control antibody (black line) was rabbit IgG (monclonal) (0.1μg/1x10^6 cells) used under the same conditions. Unlabelled sample (blue line) was also used as a control. Acquisition of >5,000 events were collected using a 20mW Argon ion laser (488nm) and 525/30 bandpass filter.

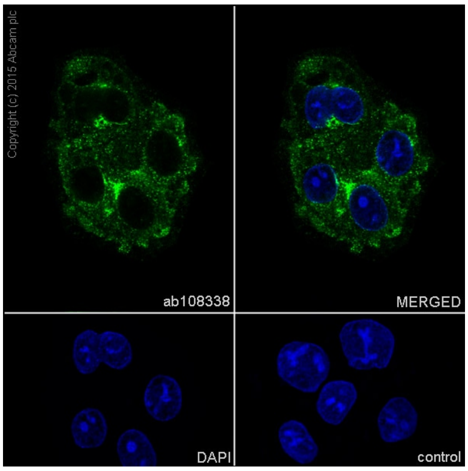

Immunocytochemistry/ Immunofluorescence - Goat Anti-Rabbit IgG H&L (Alexa Fluor® 488) (ab150077)

Immunocytochemistry/ Immunofluorescence analysis of HepG2 (Human hepatocellular carcinoma epithelial cell) cells labeling ATG9A with Purified **ab108338** at 1/100 dilution. Cells were fixed in 4% Paraformaldehyde and permeabilized with 0.1% tritonX-100. ab150077 Goat anti rabbit IgG (Alexa Fluor® 488) was used as the secondary antibody at 1/1000 dilution. DAPI nuclear counterstain. PBS instead of the primary antibody was used as the secondary antibody only control.

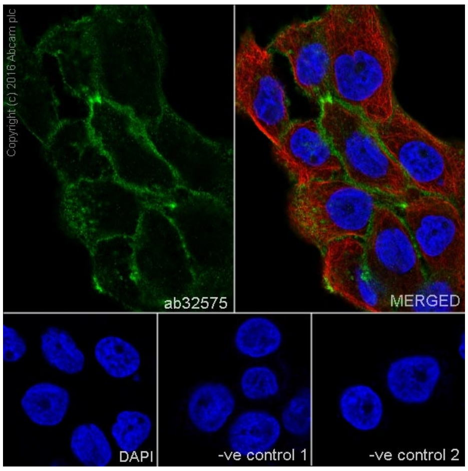

Immunocytochemistry/ Immunofluorescence - Goat Anti-Rabbit IgG H&L (Alexa Fluor® 488) (ab150077)

Immunocytochemistry/Immunofluorescence analysis of A431 (human epidermoid carcinoma) cells labeling alpha smooth muscle Actin (green) with purified **ab32575** at 1/500. Cells were fixed with 4% paraformaldehyde and permeabilized with 0.1% Triton X-100. ab150077, Alexa Fluor® 488-conjugated goat anti-rabbit IgG (1/1000) was used as the secondary antibody. Cells were counterstained with **ab7291**, anti-Tubulin (mouse mAb) at 1/1000 followed by **ab150120** Alexa Fluor®594 goat anti-mouse secondary (1/1000). Nuclei were counterstained with DAPI (blue). For negative control 1, rabbit primary antibody and anti-mouse secondary antibody (**ab150120**) were used. For negative control 2, **ab7291** (mouse primary antibody) was used followed by anti-rabbit secondary antibody (ab150077).

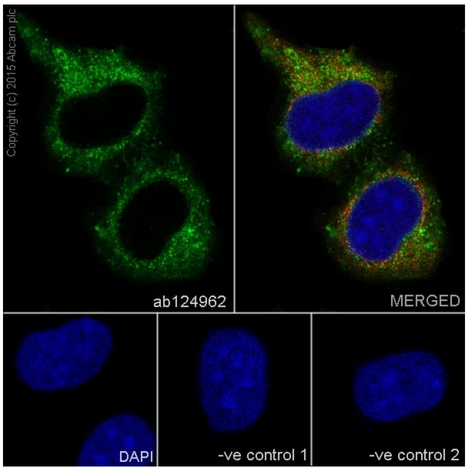

Immunocytochemistry/ Immunofluorescence - Goat Anti-Rabbit IgG H&L (Alexa Fluor® 488) (ab150077)

Immunocytochemistry/Immunofluorescence analysis of HeLa cells labelling IL-1RA with purified **ab124962** at 1/100. Cells were fixed with 100% methanol and permeabilized with 0.1% Triton X-100. ab150077, an Alexa Fluor® 488-conjugated goat anti-rabbit IgG (1/1000) was used as the secondary antibody. DAPI (blue) was used as the nuclear counterstain. **ab7291**, a mouse anti-tubulin (1/1000) and **ab150120**, an Alexa Fluor® 594-conjugated goat anti-mouse IgG (1/1000) were also used.
Control 1: primary antibody (1/100) and secondary antibody, **ab150120**, an Alexa Fluor® 594-conjugated goat anti-mouse IgG (1/1000).
Control 2: **ab7291** (1/1000) and secondary antibody, ab150077, an Alexa Fluor® 488-conjugated goat anti-rabbit IgG (1/1000).

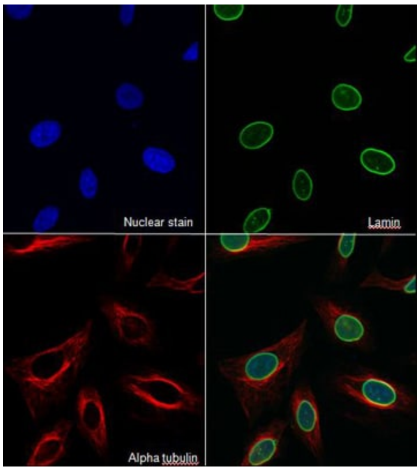

Immunocytochemistry/ Immunofluorescence - Goat Anti-Rabbit IgG H&L (Alexa Fluor® 488) (ab150077)

The cells were 100% methanol fixed (5 min) and then incubated in 1%BSA / 10% normal goat serum / 0.3M glycine in 0.1% PBS-Tween for 1h to permeabilise the cells and block non-specific protein-protein interactions. The cells were then incubated with the antibody (**ab7291**, 1µg/ml) and (**ab16048**, 1µg/ml) overnight at +4°C. The secondary antibodies were **ab150115** Alexa Fluor® 647 (red) goat anti-mouse IgG (H+L) used at 2µg/ml for 1h and ab150077 Alexa Fluor® 488 (green) goat anti-rabbit IgG (H+L) used at 2µg/ml for 1h. DAPI was used to stain the cell nuclei.

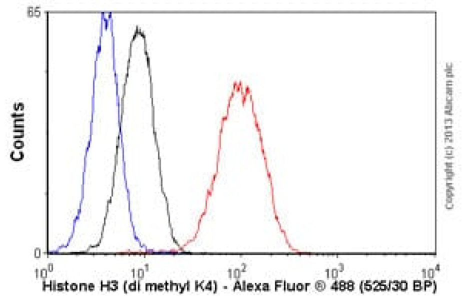

Flow Cytometry - Goat Anti-Rabbit IgG H&L (Alexa Fluor® 488) (ab150077)

Overlay histogram showing HeLa cells stained with **ab32356** (red line). The cells were fixed with 80% methanol (5 min) and then permeabilized with 0.1% PBS-Tween for 20 min. The cells were then incubated in 1x PBS / 10% normal goat serum / 0.3M glycine to block non-specific protein-protein interactions followed by the antibody (**ab32356**, 1/100 dilution) for 30 min at 22ºC. The secondary antibody used was Alexa Fluor® 488 goat anti-rabbit IgG (H&L) at 1/2000 dilution for 30 min at 22ºC. Isotype control antibody (black line) was rabbit IgG (monoclonal) (1µg/1x10^6 cells) used under the same conditions. Unlabelled sample (blue line) was also used as a control. Acquisition of >5,000 events were collected using a 20mW Argon ion laser (488nm) and 525/30 bandpass filter.

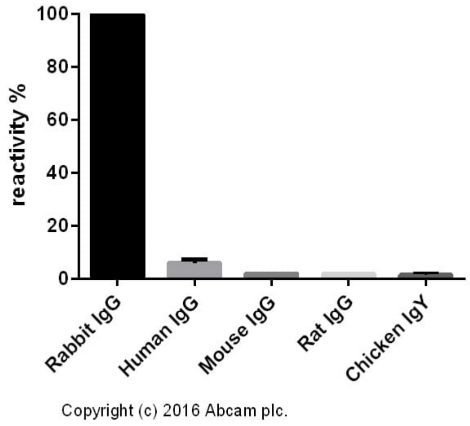

ELISA - Goat Anti-Rabbit IgG H&L (Alexa Fluor® 488) (ab150077)

Cross-reactivity of the polyclonal secondary antibody **ab182016** was tested using a sandwich ELISA approach. The wells were coated with the indicated IgG standards at 1 µg/ml (50 µl/well) and incubated overnight at 4°C, followed by a 5% BSA blocking step for 2h at RT. **ab182016** was then added starting at 1 µg/ml and gradually diluted 1/4 (50 µl/well), followed by incubation for 2h. For the detection Donkey anti-Goat IgG H&L (HRP) (**ab6885**) was used at 1/10,000 dilution (50 µl/well), followed by incubation for 1h at RT.

For the batch tested, ab182016 showed a cross-reactivity of 5-7% towards Human IgG and below 2% towards Mouse IgG, Rat IgG and Chicken IgY.

This data was developed using the unconjugated antibody (**ab182016**).

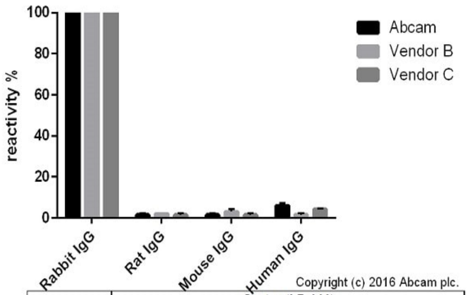

	Goat anti-Rabbit			
	Rabbit IgG	Rat IgG	Mouse IgG	Human IgG
Abcam	100%	<2%	<2%	5-7%
Vendor B	100%	2%	4%	<2%
Vendor C	100%	<2%	<2%	4-4.5%

ELISA - Goat Anti-Rabbit IgG H&L (Alexa Fluor® 488) (ab150077)

Cross-reactivity of Goat anti-Rabbit IgG H&L (**ab182016**) and Goat anti-Rabbit IgG H&L obtained from two different vendors was tested using a sandwich ELISA approach. The wells were coated with the indicated IgG standards (Rabbit, Human, Mouse and Rat) at 1 µg/ml (50 µl/well) and incubated overnight at 4°C, followed by a 5% BSA blocking step for 2h at RT. Secondary antibodies were then added starting at 1 µg/ml and gradually diluted 1/4 (50 µl/well), followed by incubation for 2h. For the detection Donkey anti-Goat IgG H&L (HRP) (**ab6885**) was used at 1/10,000 dilution (50 µl/well), followed by incubation for 1h at RT. This data is from a representative dilution.

This data was developed using the unconjugated antibody (**ab182016**).

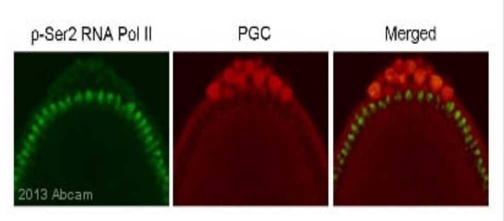

IHC - Wholemount - Goat Anti-Rabbit IgG H&L (Alexa Fluor® 488) (ab150077)

This image is courtesy of an anonymous Abreview.

IHC - Wholemount of Caenorhabditis elegans larvae labelling RNA polymerase II CTD repeat YSPTSPS (phospho S2) with **ab5095**. The sample was incubated with primary antibody (1/500 in PBS + 3% BSA + 0.1% Triton X-100) for 12 hours at 4°C. ab150077, an Alexa Fluor® 488-conjugated goat anti-rabbit IgG polyclonal (1/1000), was used as the secondary antibody.

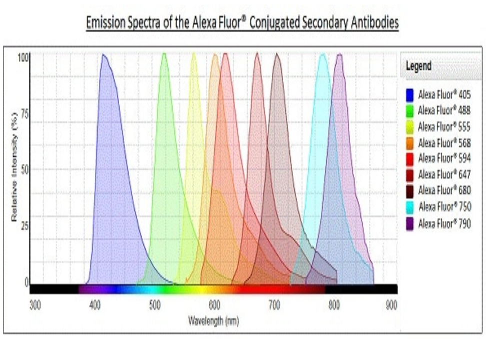

Alexa Fluor® - Goat Anti-Rabbit IgG H&L (Alexa Fluor® 488) (ab150077)

Please note: All products are "FOR RESEARCH USE ONLY. NOT FOR USE IN DIAGNOSTIC PROCEDURES"

Our Abpromise to you: Quality guaranteed and expert technical support

- Replacement or refund for products not performing as stated on the datasheet
- Valid for 12 months from date of delivery
- Response to your inquiry within 24 hours

- We provide support in Chinese, English, French, German, Japanese and Spanish
- Extensive multi-media technical resources to help you
- We investigate all quality concerns to ensure our products perform to the highest standards

If the product does not perform as described on this datasheet, we will offer a refund or replacement. For full details of the Abpromise, please visit **https://www.abcam.com/abpromise** or contact our technical team.

Terms and conditions

- Guarantee only valid for products bought direct from Abcam or one of our authorized distributors

abcam

Product datasheet

Goat Anti-Mouse IgG H&L (Alexa Fluor® 647) ab150115

★★★★★ 6 Abreviews 384 References 6 图像

概述

**产品名称**	**山羊抗小鼠**IgG H&L (Alexa Fluor® 647)
**宿主**	Goat
**靶标种属**	Mouse
**特异性**	ab150115 is specific to Mouse IgG.<br>ab150115 has less than 47% cross-reactivity with rat IgG.
**经测试应用**	**适用于:** IHC-Fr, ICC/IF, ELISA, IHC-P, Flow Cyt
**免疫原**	The details of the immunogen for this antibody are not available.
**偶联物**	Alexa Fluor® 647. Ex: 652nm, Em: 668nm

性能

**形式**	Liquid
**存放说明**	Shipped at 4°C. Store at +4°C short term (1-2 weeks). Upon delivery aliquot. Store at -20°C. Avoid freeze / thaw cycle. Stable for 12 months at -20°C. Store In the Dark.
**存储溶液**	Preservative: 0.02% Sodium azide<br>Constituents: 23% Glycerol (glycerin, glycerine), PBS, 1% BSA
**纯度**	Immunogen affinity purified
**纯化说明**	This antibody was isolated by affinity chromatography using antigen coupled to agarose beads.
**克隆**	**多克隆**
**同种型**	IgG
**常规说明**	Alexa Fluor® is a registered trademark of Molecular Probes, Inc, a Thermo Fisher Scientific Company. The Alexa Fluor® dye included in this product is provided under an intellectual property license from Life Technologies Corporation. As this product contains the Alexa Fluor® dye, the purchase of this product conveys to the buyer the non-transferable right to use the purchased product and components of the product only in research conducted by the buyer (whether the buyer is an academic or for-profit entity). As this product contains the Alexa Fluor® dye the sale of this product is expressly conditioned on the buyer not using the product or its components, or any materials made using the product or its components, in any activity to generate revenue, which may include, but is not limited to use of the product or its components: in manufacturing; (ii) to provide a service, information, or data in return for payment (iii) for therapeutic, diagnostic or

prophylactic purposes; or (iv) for resale, regardless of whether they are sold for use in research. For information on purchasing a license to this product for purposes other than research, contact Life Technologies Corporation, 5781 Van Allen Way, Carlsbad, CA 92008 USA or outlicensing@thermofisher.com.

应用

The Abpromise guarantee Abpromise™承诺保证使用ab150115于以下的经测试应用

"应用说明"部分 下显示的仅为推荐的起始稀释度；实际最佳的稀释度/浓度应由使用者检定。

应用	Ab评论	说明
IHC-Fr	★★★★☆ (2)	Use at an assay dependent concentration.
ICC/IF	★★★★★ (4)	1/200 - 1/1000.
ELISA		Use at an assay dependent concentration.
IHC-P		Use at an assay dependent concentration.
Flow Cyt		1/2000 - 1/4000.<br>ab176103 - Mouse monoclonal IgG1 (Alexa Fluor® 647), is suitable for use as an isotype control to complement this secondary antibody.

图片

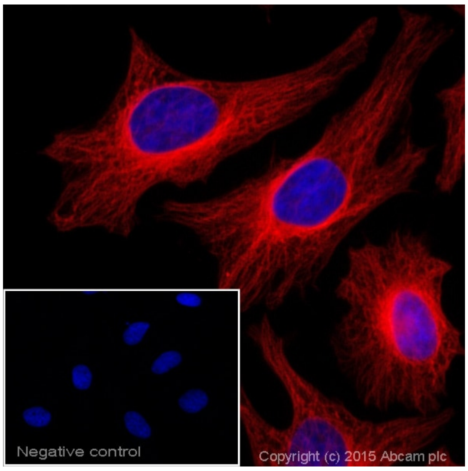

Immunocytochemistry/ Immunofluorescence - Goat Anti-Mouse IgG H&L (Alexa Fluor® 647) (ab150115)

ICC/IF image of ab7291 stained HeLa cells. The cells were 4% paraformaldehyde fixed (10 min), permeabilized with 0.1% Triton X-100 for 5 minutes and then incubated in 1% BSA / 10% normal donkey serum / 0.3M glycine in 0.1% PBS-Tween for 1h to block non-specific protein-protein interactions. The cells were then incubated with the primary antibody (ab7291, 5µg/ml) overnight at +4°C. The secondary antibody (red) was ab150115 Alexa Fluor® 647 goat anti-mouse IgG (H+L) used at 1µg/ml for 1h.DAPI was used to stain the cell nuclei (blue) at a concentration of 1.43µM.

The negative control (inset) is a secondary-only assay to demonstrate low non-specific binding of the secondary antibody.

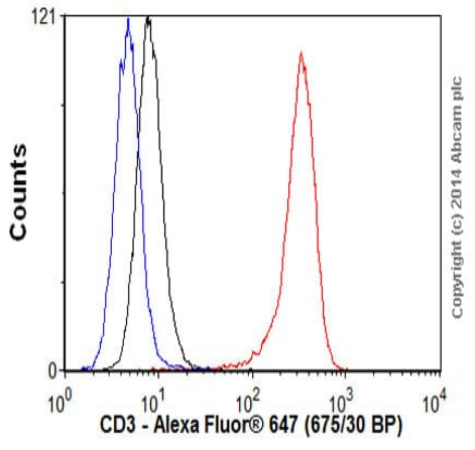

Flow Cytometry - Goat Anti-Mouse IgG H&L (Alexa Fluor® 647) (ab150115)

Overlay histogram showing Jurkat cells stained with **ab8090** (red line). The cells were fixed with 4% paraformaldehyde (10 min) and then permeabilized with 0.1% PBS-Tween for 20 min. The cells were then incubated in 1x PBS / 10% normal goat serum / 0.3M glycine to block non-specific protein-protein interactions followed by the antibody (**ab8090**, 0.1µg/1x10^6 cells) for 30 min at 22°C. The secondary antibody Goat anti-mouse IgG H&L (Alexa Fluor® 647) (ab150115) was used at 1/2000 dilution for 30 min at 22°C. Isotype control antibody (black line) was mouse IgG2a [ICIGG2A] (**ab91361**, 0.1µg/1x10^6 cells) used under the same conditions. Unlabelled sample (blue line) was also used as a control. Acquisition of >5,000 events were collected using a solid-state 25mW red diode laser (635nm) and 675/30 bandpass filter.

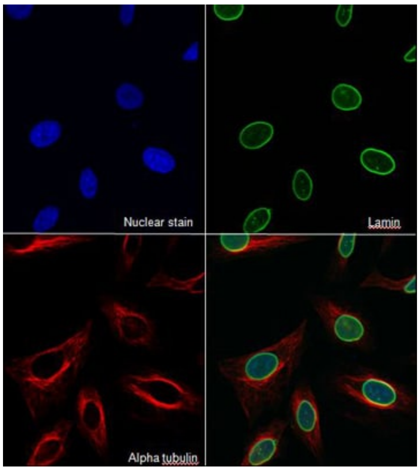

Immunocytochemistry/ Immunofluorescence - Goat Anti-Mouse IgG H&L (Alexa Fluor® 647) (ab150115)

The cells were 100% methanol fixed (5 min) and then incubated in 1% BSA / 10% normal goat serum / 0.3M glycine in 0.1% PBS-Tween for 1h to permeabilise the cells and block non-specific protein-protein interactions. The cells were then incubated with the antibody (**ab7291**, 1µg/ml) and (**ab16048**, 1µg/ml) overnight at +4°C. The secondary antibodies were ab150115 Alexa Fluor® 647 (red) goat anti-mouse IgG (H+L) used at 2µg/ml for 1h and **ab150077** Alexa Fluor® 488 (green) goat anti-rabbit IgG (H+L) used at 2µg/ml for 1h. DAPI was used to stain the cell nuclei.

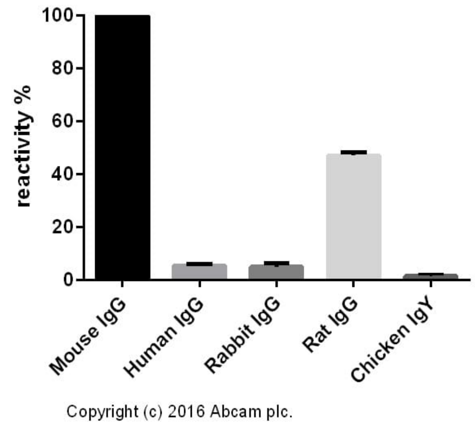

ELISA - Goat Anti-Mouse IgG H&L (Alexa Fluor® 647) (ab150115)

Cross-reactivity of the polyclonal secondary antibody **ab182017** was tested using a sandwich ELISA approach. The wells were coated with the indicated IgG standards at 1 µg/ml (50µl/well) and incubated overnight at 4°C, followed by a 5% BSA blocking step for 2h at RT. **ab182017** was then added starting at 1 µg/ml and gradually diluted 1/4 (50 µl/well), followed by incubation for 2h. For the detection Donkey anti-Goat IgG H&L (HRP) (**ab6885**) was used at 1/10,000 dilution (50 µl/well), followed by incubation for 1h at RT.

Fot the batch tested, ab182017 showed a cross-reactivity below 2% towards Chicken IgY, 6% towards Human IgG, 7% towards Rabbit IgG and 47% towards Rat IgG.

This data was developed using the unconjugated antibody (**ab182017**).

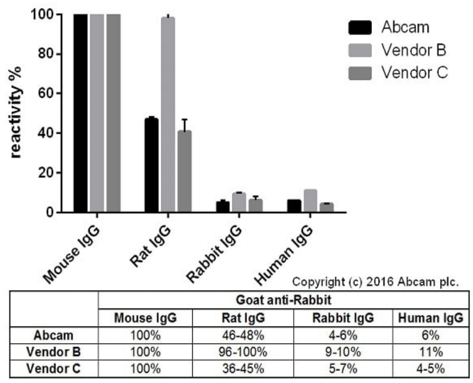

	Goat anti-Rabbit			
	Mouse IgG	Rat IgG	Rabbit IgG	Human IgG
Abcam	100%	46-48%	4-6%	6%
Vendor B	100%	96-100%	9-10%	11%
Vendor C	100%	36-45%	5-7%	4-5%

ELISA - Goat Anti-Mouse IgG H&L (Alexa Fluor® 647) (ab150115)

Cross-reactivity of Goat anti-Mouse IgG H&L (**ab182017**) and Goat anti-Mouse IgG H&L obtained from two different vendors was tested using a sandwich ELISA approach. The wells were coated with the indicated IgG standards (Rabbit, Human, Mouse and Rat) at 1 µg/ml (50µl/well) and incubated overnight at 4°C, followed by a 5% BSA blocking step for 2h at RT. Secondary antibodies were then added starting at 1 µg/ml and gradually diluted 1/4 (50 µl/well), followed by incubation for 2h. For the detection Donkey anti-Goat IgG H&L (HRP) (**ab6885**) was used at 1/10,000 dilution (50 µl/well), followed by incubation for 1h at RT. This data is from a representative dilution.

This data was developed using the unconjugated antibody (**ab182017**).

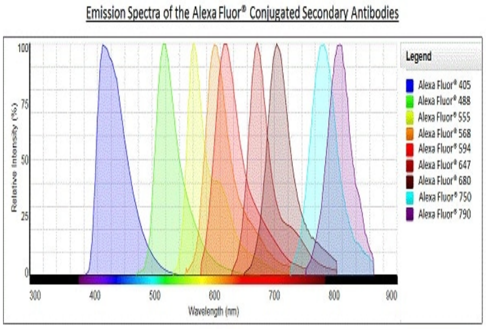

Alexa Fluor® - Goat Anti-Mouse IgG H&L (Alexa Fluor® 647) (ab150115)

Please note: All products are "FOR RESEARCH USE ONLY. NOT FOR USE IN DIAGNOSTIC PROCEDURES"

Our Abpromise to you: Quality guaranteed and expert technical support

- Replacement or refund for products not performing as stated on the datasheet
- Valid for 12 months from date of delivery
- Response to your inquiry within 24 hours

- We provide support in Chinese, English, French, German, Japanese and Spanish
- Extensive multi-media technical resources to help you
- We investigate all quality concerns to ensure our products perform to the highest standards

If the product does not perform as described on this datasheet, we will offer a refund or replacement. For full details of the Abpromise, please visit **https://www.abcam.cn/abpromise** or contact our technical team.

Terms and conditions

- Guarantee only valid for products bought direct from Abcam or one of our authorized distributors

For Research Use Only.
Not For Use In Diagnostics.

HRP-conjugated Affinipure Goat Anti-Rabbit IgG(H+L)

Catalog Number:SA00001-2

proteintech®
Antibodies | ELISA kits | Proteins
www.ptglab.com

Information

Catalog Number: SA00001-2	Reactivity: Rabbit
Host: Goat	Physical State: Liquid
Applications: ELISA, WB	Conjugation: HRP

Recommended Dilutions

1:2000-1:10,000 for western blotting with ECL substrates
1:1000-1:20,000 for ELISA and Western blotting with chromogenic substrates

Stabilizer

3 mg/ml BSA

Safety Notes

This product is for research use only,not for diagnostic or therapeutic use.

Storage

Storage:
Store at -20°C. Stable for one year after shipment.
Storage Buffer:
0.01 M Sodium phosphate, 0.25 M NaCl, 50% glycerol, 3 mg/ml BSA, pH 7.6.
Aliquoting is unnecessary for -20°C storage

Purity

The antibody was purified from antisera by immunoaffinity chromatography using antigens coupled to agarose beads.

For technical support and original validation data for this product please contact:
T: 4006900926 E: Proteintech-CN@ptglab.com W: ptgcn.com

This product is exclusively available under Proteintech Group brand and is not available to purchase from any other manufacturer.

Selected Validation Data

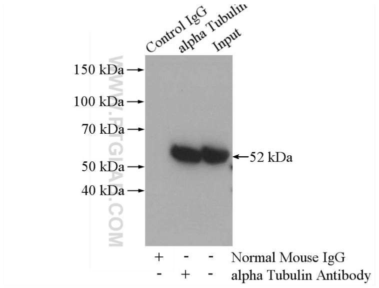

IP Result of anti-alpha Tubulin (IP: 66031-1-Ig, 5ug; Detection:11224-1-AP 1:1000) with HeLa cells lysate 2800ug. SA00001-2 (HRP-conjugated Affinipure Goat Anti-Rabbit IgG(H+L) as secondary antibody.

Cell Line Authentication Service

STR Profile Report

**The Query Sample ID:**	**2023-4008-HepG2**
**Sample Submitted By:**	**Bin Zhang**
**Institution:**	**The First Affiliated Hospital, Zhejiang University School of Medicine**
**Email Address:**	**1169797812@qq.com**
**Cell Line Designation:**	**Hep-G2(CVCL_0027)**
**Date Sample Received:**	**May.25th, 2023**
**Report Date:**	**May.31th, 2023**

Methods:

Twenty one short tandem repeat (STR) loci plus the gender locus, Amelogenin, were amplified using the commercially available EX22 Kit from AGCU. The cell line sample was processed using the ABI Prism® 3500 Genetic Analyzer. Data were analyzed using GeneMapper® ID-X software v1.6 (Applied Biosystems). Appropriate positive and negative controls were run and confirmed for the sample submitted. And the Query profile will be compared for match to STR reference profile from Cellosaurus.

Zhejiang University
310016
Forensic Science Center

268 Kaixuan Road
ShangZhangg District,

Hangzhou, Zhejiang, China
Tel: 0571-86971976, 18668219985

Results:

STR Loci	**Query Profile:** 2023-4008-HepG2 (Submitted by Zhang)	**Reference Profile:** Hep-G2 (Cellosaurus CVCL_0027)
CSF1PO	10,11	10,11
D3S1358	15,16	15,16
D5S818	11,12	11,12
D7S820	10	10
D8S1179	15,16	15,16
D13S317	9,13	9,13
D16S539	12	12,13
D18S51	13,14	13,14
D21S11	29,31	29,31
FGA	22,25	22,25
TH01	9	9
TPOX	8,9	8,9
vWA	17	17

Analysis of STR data

	**2023-4008-HepG2**	**Hep-G2**
Total Number of Distinct Alleles	22	23
Number of Distinct Alleles Shared by Both Samples	22	
Match Algorithm	[(22× 2) / (22 + 23)] × 100%	
Percent Match	98%	

The allele match algorithm compares the 13 core loci only,
even though alleles from all loci, including amelogenin locus, will be reported in the GeneMapper.
Note: These data and analysis are for research use only.

Zhejiang University
310016
Forensic Science Center

268 Kaixuan Road
ShangZhangg District,
Hangzhou, Zhejiang, China
Tel: 0571-86971976, 18668219985

Explanation:

ANSI/ATCC ASN-0002 Revised 2021 Authentication Of Human Cell Lines: Standardization of Short Tandem Repeat (STR) Profiling recommends the use of a modified Tanabe Match algorithm:

$$\text{Tanabe \% Match}=\left[\frac{2\times(\text{N° of STR alleles shared between Query and Reference Profiles})}{(\text{N° of STR alleles in Query Profile})+(\text{N° of STR alleles in Reference Profile})}\right]\times 100\%$$

In this Standard, count single peaks once. Count only the alleles at shared STR loci. The alleles at the amelogenin loci are excluded from this calculation.

Two samples are considered possibly related when their STR profiles match at 80-90% of alleles across the 13 core STR loci, as calculated using the matching algorithm. At matches of 90% or greater, the cell lines have very likely originated from the same donor. Samples between 60 and 79% match tend to be unrelated, but need may further investigation to confirm that they are unrelated.

Note: Percent matches of even 100% only indicate that the cell lines or tissue samples are derived from the same donor and do not mean the samples are genetically identical. STR analysis examines only a very small portion (< 0.0004%) of the entire genome.

Summary:

The Query STR profile: 2023-4008-HepG2 (Submitted by Bin Zhang) and the Reference STR profile: Hep-G2 (Cellosaurus CVCL_0027) have a Percent Match score of 98% by using the Tanabe algorithm to compare, which indicates that the cell lines have very likely originated from the same donor.

Electrophoretogram:

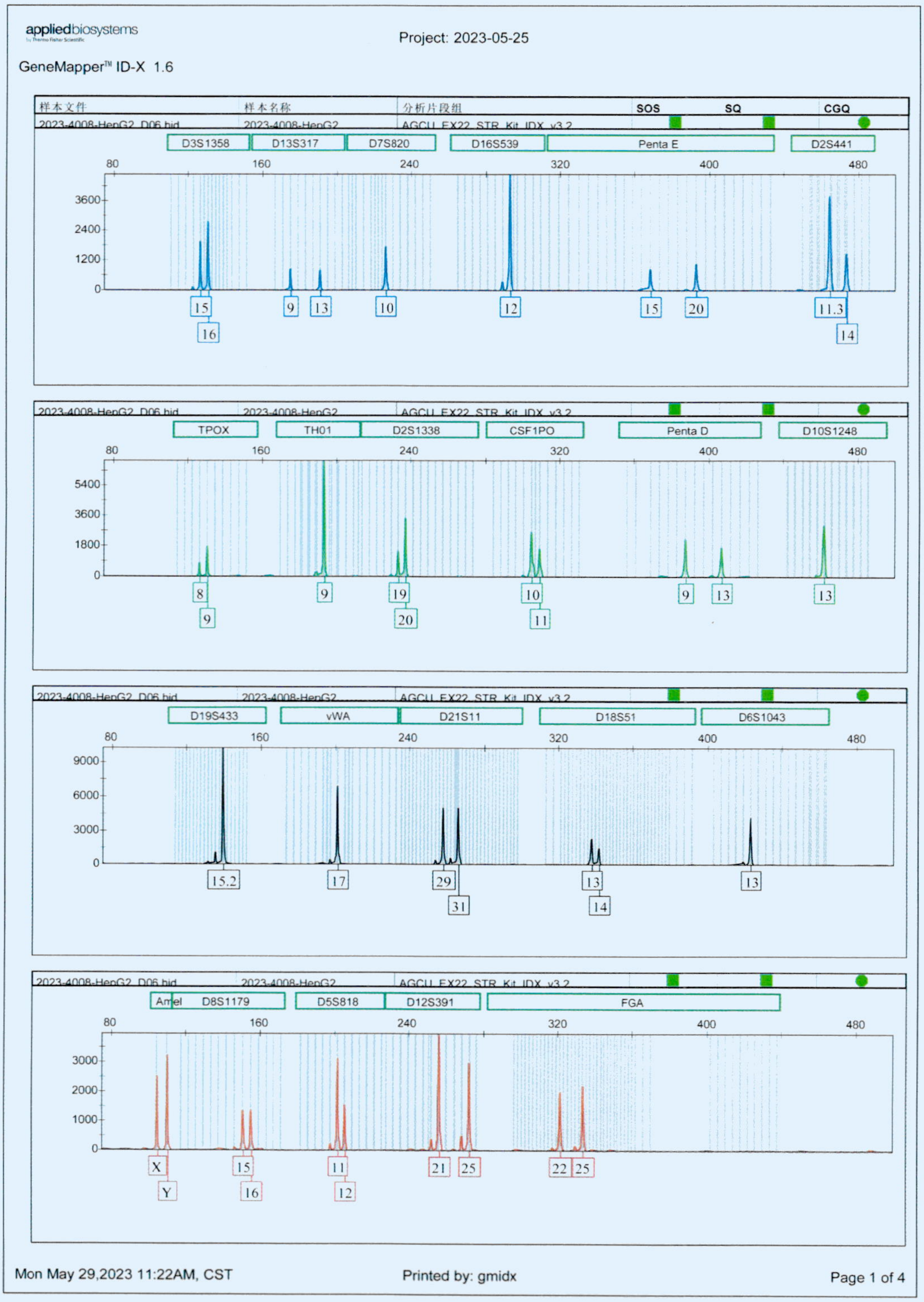

Zhejiang University
310016
Forensic Science Center

268 Kaixuan Road
ShangZhangg District,
Hangzhou, Zhejiang, China
Tel: 0571-86971976, 18668219985

Cell Line Authentication Service

STR Profile Report

**The Query Sample ID:**	**2023-4009-Huh7**
**Sample Submitted By:**	**Bin Zhang**
**Institution:**	**The First Affiliated Hospital, Zhejiang University School of Medicine**
**Email Address:**	**1169797812@qq.com**
**Cell Line Designation:**	**Huh-7(CVCL_0336)**
**Date Sample Received:**	**May.25th, 2023**
**Report Date:**	**May.31th, 2023**

Methods:

Twenty one short tandem repeat (STR) loci plus the gender locus, Amelogenin, were amplified using the commercially available EX22 Kit from AGCU. The cell line sample was processed using the ABI Prism® 3500 Genetic Analyzer. Data were analyzed using GeneMapper® ID-X software v1.6 (Applied Biosystems). Appropriate positive and negative controls were run and confirmed for the sample submitted. And the Query profile will be compared for match to STR reference profile from Cellosaurus.

Zhejiang University
310016
Forensic Science Center

268 Kaixuan Road
ShangZhangg District,

Hangzhou, Zhejiang, China
Tel: 0571-86971976, 18668219985

Zhejiang University Forensic Science Center

Results:

STR Loci	**Query Profile:** 2023-4009-Huh7 (Submitted by Zhang)	**Reference Profile:** Huh-7 (Cellosaurus CVCL_0336)
CSF1PO	11	11
D3S1358	15	15
D5S818	12	12
D7S820	11	11
D8S1179	14	14,15
D13S317	10,11	10,11
D16S539	10	10
D18S51	15	15
D21S11	30	30
FGA	22,23	22,23
TH01	7	7
TPOX	8,11	8,11
vWA	18	16,18

Analysis of STR data

	**2023-4009-Huh7**	**Huh-7**
Total Number of Distinct Alleles	16	18
Number of Distinct Alleles Shared by Both Samples	16	
Match Algorithm	[(16× 2) / (16 + 18)] × 100%	
Percent Match	94%	

The allele match algorithm compares the 13 core loci only,
even though alleles from all loci, including amelogenin locus, will be reported in the GeneMapper.
Note: These data and analysis are for research use only.

Zhejiang University
310016
Forensic Science Center

268 Kaixuan Road
ShangZhangg District,
Hangzhou, Zhejiang, China
Tel: 0571-86971976, 18668219985

Explanation:

ANSI/ATCC ASN-0002 Revised 2021 Authentication Of Human Cell Lines: Standardization of Short Tandem Repeat (STR) Profiling recommends the use of a modified Tanabe Match algorithm:

$$\text{Tanabe \% Match}=\left[\frac{2\times(\text{N° of STR alleles shared between Query and Reference Profiles})}{(\text{N° of STR alleles in Query Profile})+(\text{N° of STR alleles in Reference Profile})}\right]\times 100\%$$

In this Standard, count single peaks once. Count only the alleles at shared STR loci. The alleles at the amelogenin loci are excluded from this calculation.

Two samples are considered possibly related when their STR profiles match at 80-90% of alleles across the 13 core STR loci, as calculated using the matching algorithm. At matches of 90% or greater, the cell lines have very likely originated from the same donor. Samples between 60 and 79% match tend to be unrelated, but need may further investigation to confirm that they are unrelated.

Note: Percent matches of even 100% only indicate that the cell lines or tissue samples are derived from the same donor and do not mean the samples are genetically identical. STR analysis examines only a very small portion (< 0.0004%) of the entire genome.

Summary:

The Query STR profile: 2023-4009-Huh7 (Submitted by Bin Zhang) and the Reference STR profile: Huh-7 (Cellosaurus CVCL_0336) have a Percent Match score of 94% by using the Tanabe algorithm to compare, which indicates that the cell lines have very likely originated from the same donor.

Zhejiang University
310016
Forensic Science Center

268 Kaixuan Road
ShangZhangg District,
Hangzhou, Zhejiang, China
Tel: 0571-86971976, 18668219985

Electrophoretogram:

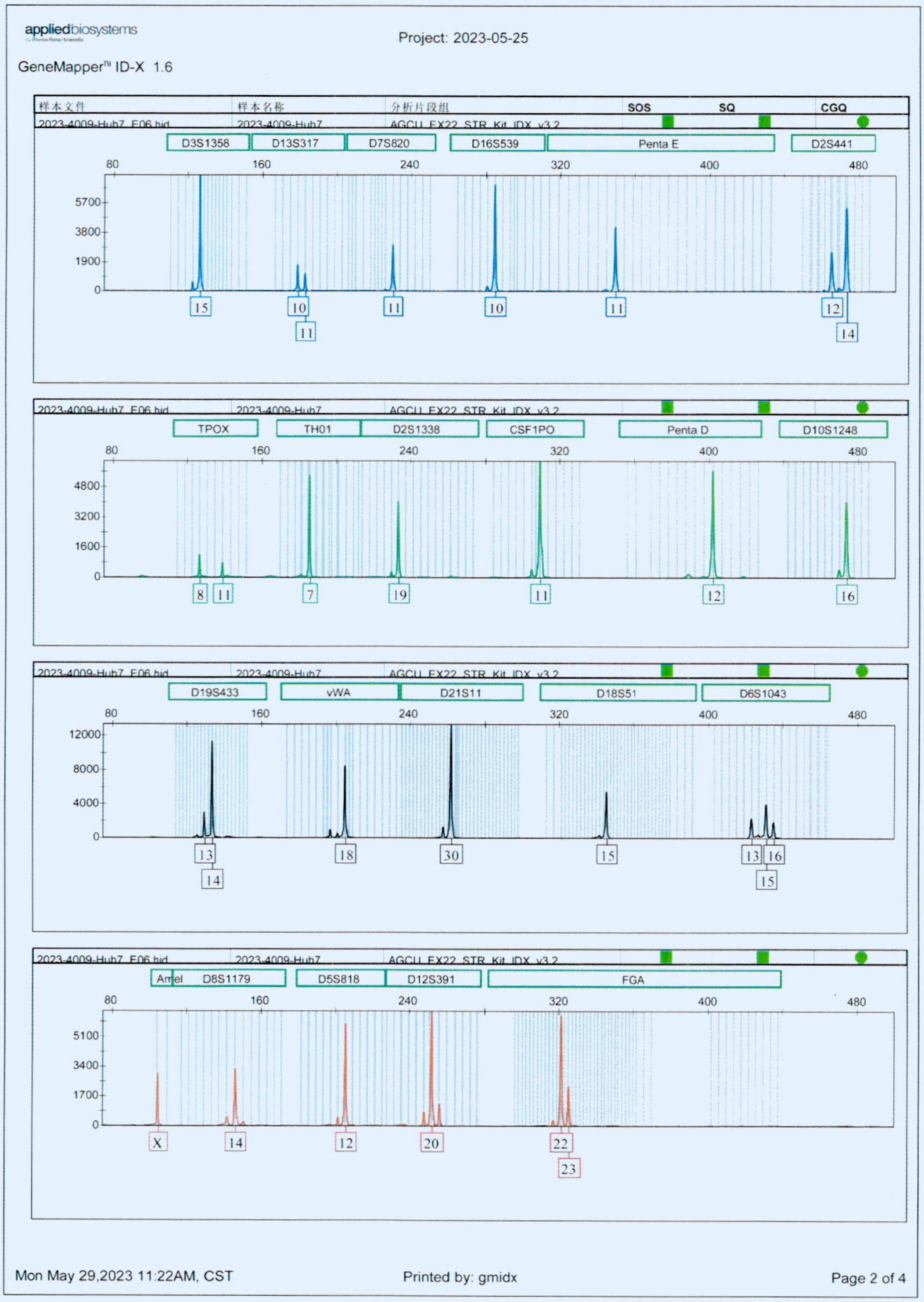

Zhejiang University
310016
Forensic Science Center

268 Kaixuan Road
ShangZhangg District,

Hangzhou, Zhejiang, China
Tel: 0571-86971976, 18668219985

Cell Line Authentication Service

STR Profile Report

**The Query Sample ID:**	**2023-4011-L-02**
**Sample Submitted By:**	**Bin Zhang**
**Institution:**	**The First Affiliated Hospital, Zhejiang University School of Medicine**
**Email Address:**	**1169797812@qq.com**
**Cell Line Designation:**	**L-02(CVCL_6926)**
**Date Sample Received:**	**May.25th, 2023**
**Report Date:**	**May.31th, 2023**

Methods:

Twenty one short tandem repeat (STR) loci plus the gender locus, Amelogenin, were amplified using the commercially available EX22 Kit from AGCU. The cell line sample was processed using the ABI Prism® 3500 Genetic Analyzer. Data were analyzed using GeneMapper® ID-X software v1.6 (Applied Biosystems). Appropriate positive and negative controls were run and confirmed for the sample submitted. And the Query profile will be compared for match to STR reference profile from Cellosaurus.

Results:

STR Loci	Query Profile:<br>2023-4011-L-02<br>(Submitted by Zhang)	Reference Profile:<br>L-02<br>(Cellosaurus CVCL_6926)
CSF1PO	9,10	10
D3S1358	15,18	15,18
D5S818	11,12	11,12
D7S820	8,12	12
D8S1179	12,13	12
D13S317	12,13.3	13.3
D16S539	9,10	9,10
D18S51	16	16
D21S11	27,28	27,28
FGA	21	18,21
TH01	7	7
TPOX	8,12	12
vWA	16,18	16,18
**Analysis of STR data**		
	**2023-4011-L-02**	**L-02**
Total Number of Distinct Alleles	23	19
Number of Distinct Alleles Shared by Both Samples	18	
Match Algorithm	[(18× 2) / (23 + 19)] × 100%	
Percent Match	86%	

The allele match algorithm compares the 13 core loci only,
even though alleles from all loci, including amelogenin locus, will be reported in the GeneMapper.
Note: These data and analysis are for research use only.

Zhejiang University
310016
Forensic Science Center

268 Kaixuan Road
ShangZhangg District,

Hangzhou, Zhejiang, China
Tel: 0571-86971976, 18668219985

Explanation:

ANSI/ATCC ASN-0002 Revised 2021 Authentication Of Human Cell Lines: Standardization of Short Tandem Repeat (STR) Profiling recommends the use of a modified Tanabe Match algorithm:

$$\text{Tanabe \% Match}=\left[\frac{2\times(\text{N° of STR alleles shared between Query and Reference Profiles})}{(\text{N° of STR alleles in Query Profile})+(\text{N° of STR alleles in Reference Profile})}\right]\times 100\%$$

In this Standard, count single peaks once. Count only the alleles at shared STR loci. The alleles at the amelogenin loci are excluded from this calculation.

Two samples are considered possibly related when their STR profiles match at 80-90% of alleles across the 13 core STR loci, as calculated using the matching algorithm. At matches of 90% or greater, the cell lines have very likely originated from the same donor. Samples between 60 and 79% match tend to be unrelated, but need may further investigation to confirm that they are unrelated.

Note: Percent matches of even 100% only indicate that the cell lines or tissue samples are derived from the same donor and do not mean the samples are genetically identical. STR analysis examines only a very small portion (< 0.0004%) of the entire genome.

Summary:

The Query STR profile: 2023-4011-L-02 (Submitted by Bin Zhang) and the Reference STR profile: L-02 (Cellosaurus CVCL_6926) have a Percent Match score of 86% by using the Tanabe algorithm to compare, which indicates that the cell lines are likely to be from the same donor.

Zhejiang University
310016
Forensic Science Center

268 Kaixuan Road
ShangZhangg District,

Hangzhou, Zhejiang, China
Tel: 0571-86971976, 18668219985

Electrophoretogram:

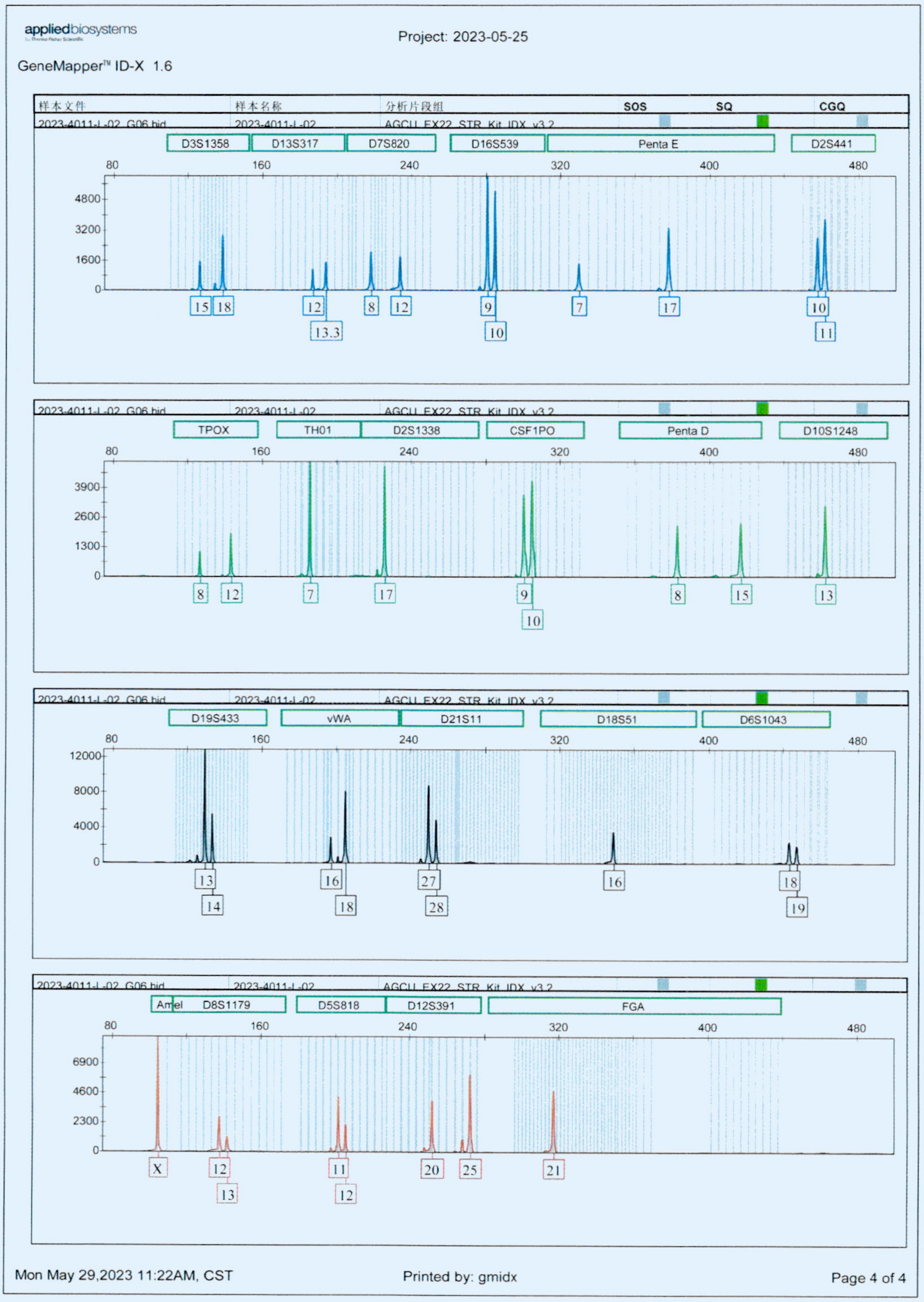

Zhejiang University
310016
Forensic Science Center

268 Kaixuan Road
ShangZhangg District,

Hangzhou, Zhejiang, China
Tel: 0571-86971976, 18668219985

Cell Line Authentication Service

STR Profile Report

**The Query Sample ID:**	**2023-4010-HepG2.2.15**
**Sample Submitted By:**	**Bin Zhang**
**Institution:**	**The First Affiliated Hospital, Zhejiang University School of Medicine**
**Email Address:**	**1169797812@qq.com**
**Cell Line Designation:**	**Hep-G2/2.2.15(CVCL_L855)**
**Date Sample Received:**	**May.25th, 2023**
**Report Date:**	**May.31th, 2023**

Methods:

Twenty one short tandem repeat (STR) loci plus the gender locus, Amelogenin, were amplified using the commercially available EX22 Kit from AGCU. The cell line sample was processed using the ABI Prism® 3500 Genetic Analyzer. Data were analyzed using GeneMapper® ID-X software v1.6 (Applied Biosystems). Appropriate positive and negative controls were run and confirmed for the sample submitted. And the Query profile will be compared for match to STR reference profile from Cellosaurus.

Zhejiang University
310016
Forensic Science Center

268 Kaixuan Road
ShangZhangg District,

Hangzhou, Zhejiang, China
Tel: 0571-86971976, 18668219985

Zhejiang University Forensic Science Center

Results:

STR Loci	**Query Profile:**<br>2023-4010-HepG2.2.15<br>(Submitted by Zhang)	**Reference Profile:**<br>Hep-G2/2.2.15<br>(Cellosaurus CVCL_L855)
CSF1PO	10,11	10,11
D3S1358	16	15,16
D5S818	11,12	11,12
D7S820	10	10
D8S1179	15,16	15,16
D13S317	9,13	9,13
D16S539	12	12,13
D18S51	13,14	13,14
D21S11	29,31	29,31
FGA	22,25	22,25
TH01	9	9
TPOX	8,9	8,9
vWA	17	17
**Analysis of STR data**		
	**2023-4010-HepG2.2.15**	**Hep-G2/2.2.15**
Total Number of Distinct Alleles	21	23
Number of Distinct Alleles Shared by Both Samples	21	
Match Algorithm	[(21× 2) / (21 + 23)] × 100%	
Percent Match	95%	

The allele match algorithm compares the 13 core loci only,
even though alleles from all loci, including amelogenin locus, will be reported in the GeneMapper.
Note: These data and analysis are for research use only.

Zhejiang University
310016
Forensic Science Center

268 Kaixuan Road
ShangZhangg District,

Hangzhou, Zhejiang, China
Tel: 0571-86971976, 18668219985

Explanation:

ANSI/ATCC ASN-0002 Revised 2021 Authentication Of Human Cell Lines: Standardization of Short Tandem Repeat (STR) Profiling recommends the use of a modified Tanabe Match algorithm:

$$\text{Tanabe \% Match}=\left[\frac{2\times(\text{N° of STR alleles shared between Query and Reference Profiles})}{(\text{N° of STR alleles in Query Profile})+(\text{N° of STR alleles in Reference Profile})}\right]\times 100\%$$

In this Standard, count single peaks once. Count only the alleles at shared STR loci. The alleles at the amelogenin loci are excluded from this calculation.

Two samples are considered possibly related when their STR profiles match at 80-90% of alleles across the 13 core STR loci, as calculated using the matching algorithm. At matches of 90% or greater, the cell lines have very likely originated from the same donor. Samples between 60 and 79% match tend to be unrelated, but need may further investigation to confirm that they are unrelated.

Note: Percent matches of even 100% only indicate that the cell lines or tissue samples are derived from the same donor and do not mean the samples are genetically identical. STR analysis examines only a very small portion (< 0.0004%) of the entire genome.

Summary:

The Query STR profile: 2023-4010-HepG2.2.15 (Submitted by Bin Zhang) and the Reference STR profile: Hep-G2/2.2.15 (Cellosaurus CVCL_L855) have a Percent Match score of 95% by using the Tanabe algorithm to compare, which indicates that the cell lines have very likely originated from the same donor.

Zhejiang University
310016
Forensic Science Center

268 Kaixuan Road
ShangZhangg District,

Hangzhou, Zhejiang, China
Tel: 0571-86971976, 18668219985

Electrophoretogram:

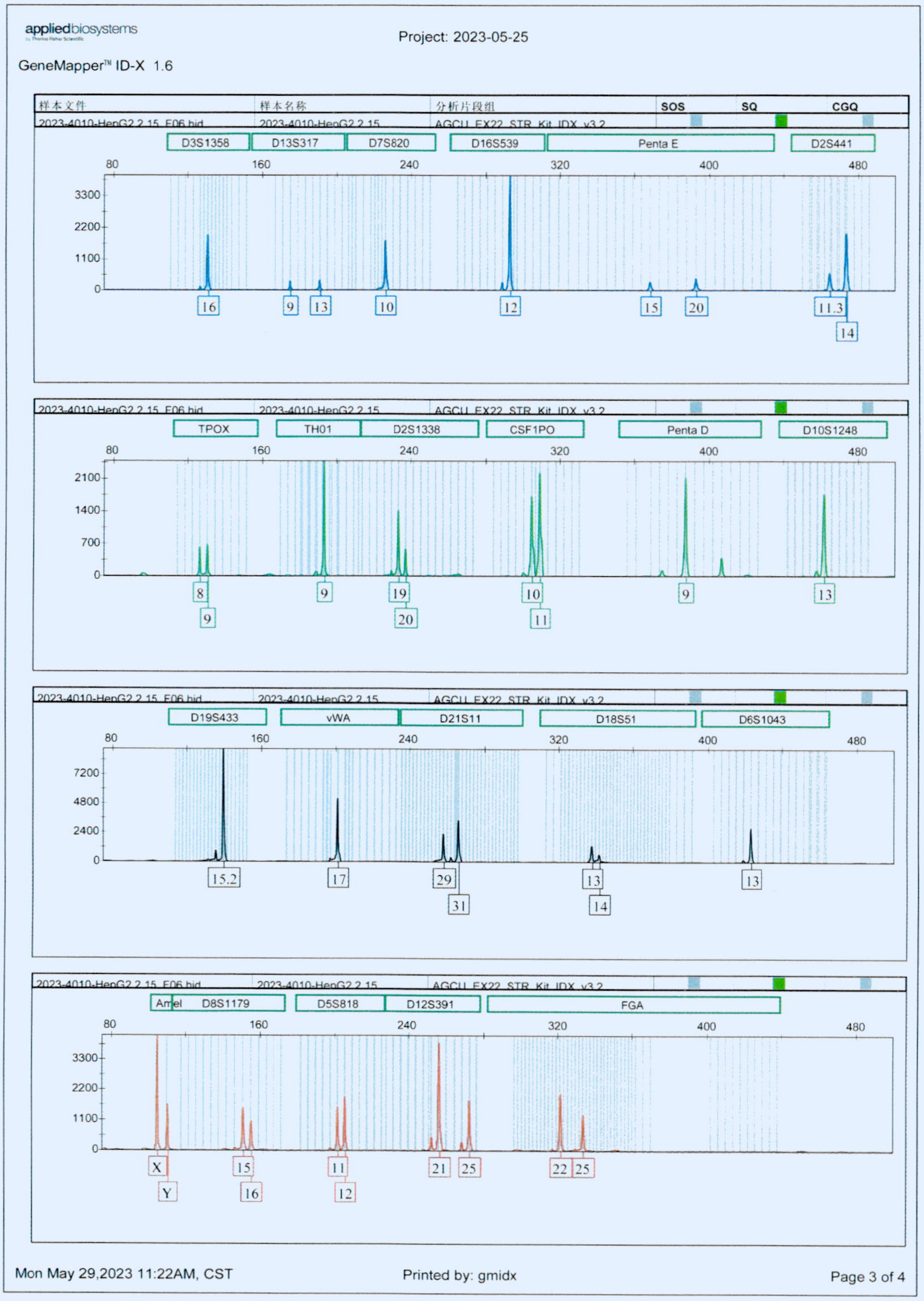

Zhejiang University
310016
Forensic Science Center

268 Kaixuan Road
ShangZhangg District,
Hangzhou, Zhejiang, China
Tel: 0571-86971976, 18668219985
